# Prevention of childhood unintentional injuries in low- and middle-income countries: A systematic review

**DOI:** 10.1371/journal.pone.0243464

**Published:** 2020-12-29

**Authors:** Anna Tupetz, Kaitlyn Friedman, Duan Zhao, Huipeng Liao, Megan Von Isenburg, Elizabeth M. Keating, Joao Ricardo Nickenig Vissoci, Catherine A. Staton

**Affiliations:** 1 Duke Global Health Institute, Durham, North Carolina, United States of America; 2 Department of Emergency Medicine, Duke University Medical Center, Durham, North Carolina, United States of America; 3 Duke Kunshan University, Kunshan, Suzhou, Jiangsu, China; 4 Division of Pediatric Emergency Medicine, Division of Public Health, University of Utah, Salt Lake City, Utah, United States of America; University of South Florida, UNITED STATES

## Abstract

Injuries are a leading cause of death and disability among children. Numerous injury prevention strategies have been successful in high-income countries, but the majority of unintentional injuries happen to children living in low- and middle-income countries (LMICs). This project aims to delineate the childhood injury prevention initiatives in LMICs. For inclusion, peer-reviewed articles needed to address unintentional injury, include children <18, assess a prevention-related intervention, contain a control group, and be published after 1988. Two pairs of reviewers evaluated articles independently to determine study eligibility. 74 articles were included. 30 studies addressed road traffic injuries, 11 drowning, 8 burns, 3 falls, 8 poisonings, and 21 an unspecified injury type. The findings show positive effects on injury outcome measures following educational interventions, the need for longer follow-up periods after the intervention, the need for effectiveness trials for behavior change, and the need for an increase in injury prevention services in LMICs. This is the first systematic review to summarize the prevention initiatives for all types of childhood unintentional injuries in LMICs. Increased attention and funding are required to go beyond educational initiatives with self-reported measures and little follow-up time to robust interventions that will reduce the global burden of unintentional injuries among children.

## Introduction

Five million deaths are attributed to injury globally every year, and 12% of these are among children [1]. Globally, injuries are a leading cause of death and disability among children [2]. Over 900,000 children under the age of 18 die every year due to unintentional injuries [2]. With this review, we identified the current state of childhood injury prevention programs in low and middle-income countries (LMICs), including seemingly effective intervention methods as well as challenges and gaps in current research efforts.

While most reports focus on injury mortality rates, morbidity is another important factor to consider when estimating the impact of injuries on the individual, the society, and the health care system. It is well established that children who have disabilities are generally more likely “to die young, or be neglected, malnourished and poor” [3]. The Child Injury Pyramid is a known concept that visualizes the enormous number of children being injured and requiring medical attention for each death reported [2]. In a sample of 250,000 people in 5 different Southeast Asian countries, UNICEF and the Alliance for Safe Children found that for each reported death in children, 12 children were admitted to the hospital or were permanently disabled, while 34 children required medical care or were unable to attend work/school due to the injury [4]. Therefore, the prevention of childhood injuries in LMICs is critical to decrease the global burden and limit the detrimental impact childhood injuries can have on individuals, their families, and health care systems [5].

The World Health Organization (WHO) released a report on child injury prevention in 2008, detailing the main injury types and ways of prevention, since we cannot assume that prevention strategies that are successful in high-income countries (HICs) will be equally effective and realizable in LMIC settings [6]. The five major categories defined by the WHO include road traffic injuries (RTI), drowning, burns, falls, and poisonings [2].

Ninety-three percent of all child mortality due to RTI occurs in LMICs [2]. By 2030, RTIs are predicted to be the fifth leading cause of death and the seventh leading cause of loss of disability-adjusted life years (DALYs) worldwide. In regard to drowning, 98% of incidents occur in LMICs, particularly in rural areas with open bodies of water [2]. Children under the age of five are at the greatest risk for drowning and drowning survivors can suffer permanent neurological damage [7, 8]. For burns, the mortality rate varies greatly between LMICs and HICs, at 4.3 per 100,000 vs. 0.4 per 100,000, respectively. Burns are the only category of unintentional injuries in which females are at higher risk than males. While understudied, reports have found higher rates of self-harm in young females as a result of domestic violence, as well as a direct form of interpersonal violence towards females, which could have been reported as unintentional injuries instead [9, 10]. The increased risk in Southeast Asia specifically is associated with open cooking equipment and low socioeconomic status [11]. In a review on burns in SSA, the leading cause of burns was scalds with 59%, followed by flames in 33%. The male-to-female ratio was almost equal, and burns disproportionately affected children below the age of 10 years with 83% of reported burns [12].

The majority of mortality caused by falls is seen in older adults, but non-fatal falls are a major cause of loss of DALYs in children under the age of 15 [2]. There is a strong association with fall mortality and socioeconomic status, as many prevention initiatives are more widely used in HICs [13, 14]. Fatal poisoning rates are more than four times higher in LMICs compared with HICs, with acute poisonings often related to fuels commonly used in households for cooking and lighting like paraffin or kerosene [2]. Poisonings as a result of domestic violence may also be reported as “unintentional” in hospital settings [15].

More than 10 years after this WHO report, much work remains to be done, particularly in LMICs. Numerous cost-effective injury prevention strategies have been proven successful in HICs, but the majority of these unintentional injuries happen to children living in LMICs [15].

While injuries are often predictable and preventable, due to many existing effective and low-cost prevention initiatives, they are not widely evaluated among children in LMICs, who are particularly vulnerable to risk factors for injury [16–19]. It is therefore necessary to increase research in and awareness of effective prevention initiatives for childhood injuries that are applicable in LMIC settings [18, 20]. This must be done to decrease the substantial economic burden on society, the individual, and health care and health insurance systems [21]. There is a great need for coordination between and among countries facing this burden to create solutions that are scalable and context appropriate.

A systematic review by Vecino-Ortiz et al. assessed effective interventions for unintentional injuries among the world’s poorest billion [22]. While this review identifies interventions to reduce mortality, our systematic review included a variety of outcomes and focused exclusively on interventions for children. Rather than assessing the poorest billion from all countries, our review examined interventions from all countries classified as LMIC, allowing for the consideration of geopolitical structures and opportunities for implementing interventions. This systematic review aggregates and summarizes the prevention initiatives for all types of childhood unintentional injuries in LMICs and is inclusive of primary research and additional injury types. With this review we will present what strategies have been proven to be most effective for 5 different injury types and different geographical locations, as well as current gaps in the knowledge of injury prevention strategies.

## Methods

### Protocol and registration

This systematic review is reported in accordance with the Preferred Reporting Items for Systematic Review and Meta-Analyses (PRISMA) Statement (S3 Appendix) and is registered in the PROSPERO database (International Prospective Register of Systematic Reviews) under the number CRD42018091453 [23].

### Eligibility criteria

Our main criteria for article inclusion were assessment of a prevention initiative for unintentional injuries in children in LMICs. LMIC status and categories of unintentional injuries (RTIs, drowning, burns, falls, and poisonings) were decided according to World Bank and WHO criteria, respectively [24, 25]. As of the 2021 fiscal year, countries with a gross national income (GNI) lower than $1,035 were classified as low-income countries (LICs), and LMICs include countries with a GNI between $1,036 and $4,045 [24]. For inclusion, articles needed to be related to unintentional injury, target or include children under the age of 18, assess a prevention-related intervention, contain a control or comparison group (including pre-post designs), and be peer-reviewed and published after 1988. 1988 was chosen as the cut-off date to capture as many as studies as possible within a reasonable timeframe (30 years). Injuries resulting from self-harm behavior were not included. Articles were excluded if they were abstracts, literature or systematic reviews, meta-analysis, unpublished theses, or commentaries.

### Information sources

We searched the electronic databases PubMed, Scopus, Embase, and Global Index Medicus (formerly Global Health Library). No exclusions were made based on the language of the article. Reference analysis was conducted manually, and citation analysis was conducted using Web of Science and Google Scholar.

### Search

B1 in S2 Appendix shows the search terms used in the electronic databases in February 2018. After initial data analysis, electronic databases were re-screened for articles published between February 2018 and April 2019 (B2 in S2 Appendix) and April 2019 and May 2020 (B3 in S2 Appendix).

### Study selection and data collection

**Fig 1:**
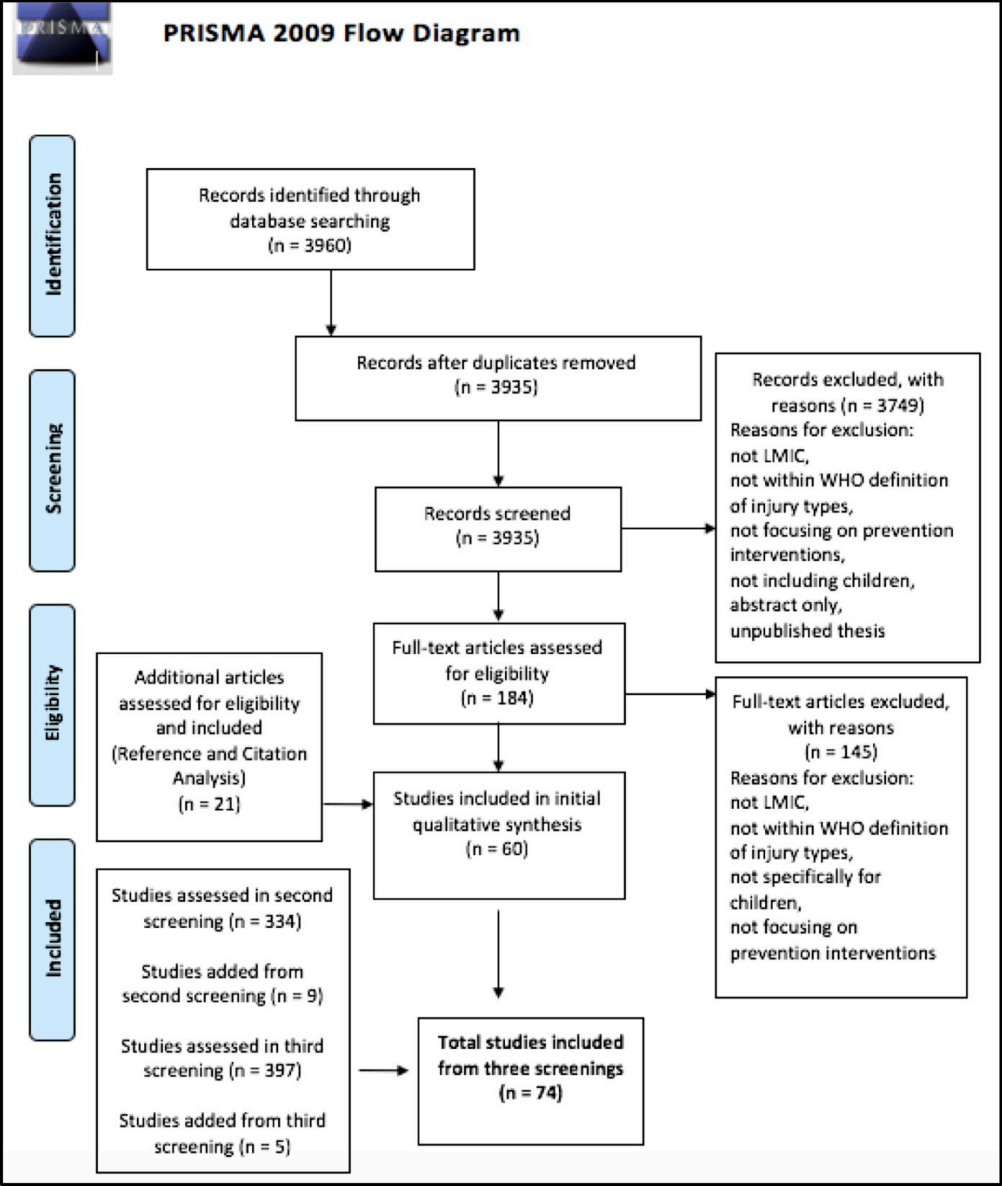
PRISMA flow diagram.

We found a total of 3960 articles in our initial search. Two reviewers independently screened the titles and abstracts. Abstracts not providing sufficient information concerning the eligibility criteria were accessed for full-text review. Two pairs of reviewers then evaluated full-text articles independently to determine study eligibility in the original study language. Reference and citation analysis were done on the articles meeting inclusion criteria. Sixty articles were included from this search (Fig 1).

We updated the same search criteria to include studies published after the initial search (between February 2018 and April 2019. This resulted in an additional 344 articles. These articles went through the same inclusion and screening process as the initial search. Nine articles were included from this search. A third search was conducted for studies published between April 2019 and May 2020, resulting in an additional 397 articles to be screened. Following the same inclusion and screening process, five articles were added from this search.

### Quality of studies

To assess data quality we used the Cochrane RoB 2 tool (A1 in S1 Appendix). The Cochrane RoB 2 tool [26] assesses risk of bias by asking questions about the study design, aim of the study, randomization process, deviation from intended interventions, missing outcome data, measurement of the outcome, and selection of reported result. The randomized control trials RCTs were also assessed using the *Consolidated Standards of Reporting Trials* (CONSORT) (A2 in S1 Appendix). However, in order to have a standardized classification of bias for all studies, we classified studies as low, moderate, or high risk of bias as outlined in the Cochrane handbook [27]. No studies were excluded from data extraction based on their assigned quality.

### Data extraction

Two pairs of reviewers conducted data extraction on articles independently, and disagreements were resolved through discussion. The data extraction included year of publication, geographic region of author, location of study, objective, study design, setting, intervention type, sample size, participant characteristics and inclusion criteria, data collection and analysis methods, reported outcomes and values, results, and main conclusions. For non-English articles, data extraction was conducted by a bilingual researcher that was a fluent or native speaker in English and the language of the article.

### Data analysis

Upon screening the articles for this review, it was concluded that a meta-analytical approach of all of the articles would not be feasible given the high level of variability in study designs. We thus conducted a qualitative metasummary. Thematic analysis was done by aggregating the main outcomes of the articles by categories of injury and type of intervention.

## Results

### Study characteristics

In total, 74 articles were included in this review (Table 1). Fifty articles were in English, 13 in Chinese, 5 in Portuguese, and 6 in Spanish (Table 1).

**Table 1:** Study characteristics.

Authors	Year	Language of Article (Location of Study)	Study Design	Risk of Bias	Intervention Type	Study Follow up time (post-test)	Targeted Population & Setting	Sample Size	Outcome Measures (Tool Used)	Author Conclusions
***Road Traffic Injuries***										
Abreu, D. R. O. M., De Souza, E. M. & Mathias, T. A. F. [28]	2018	Portugese (Brazil)	Ecological time series	Low	Legislation	N/A	State of Paraná, Brazil—residents aged 15–49	Not reported	Mortality (Mortality Information System)	“Following enactment of the Drinking and Driving Law, the data displayed variability and the trends were not significant. However, there was a decrease in overall and pedestrian mortality. The rates for motorcyclists and vehicle occupants stabilized. The results showed an impact on traffic accident mortality after enactment of the new Brazilian Traffic Code and Drinking and Driving Law, followed by an increase in the rates.”
Ahmad H, Naeem R, Feroze A, Zia N, Shakoor A, Khan UR, Mian, AI [29]	2018	English (Pakistan)	Pretest- Posttest, one group	Moderate	Education	0–2 months	Students 8–16 years old in Pakistan	410 students, 17 schools	Knowledge of prevention measures (multiple choice questionnaire in English & Urdu)	"Bilingual pictorial story books can help helped primary school children to learn about RTI prevention and may be incorporated into school curricula, possibly adaptable in different languages and communities."
Charry, JD, Ochoa, JD, Tejada, JH, Navarro-Parra, SL, Esquivel, N, & Vasques, Y [30]	2017	English (Colombia)	Pretest-Posttest, one group	Moderate	Education	Not specified	Adolescents in Colombia	160 high school seniors	Knowledge of vehicle safety devices and risks of alcohol consumption (pre and post interventional surveys on the use of vehicle safety devices and attitudes towards alcohol consumption and driving)	"In conclusion, based on our experience, a prevention-oriented model for traffic accidents proves to be effective in generating changes in adolescents' behavior regarding and attitudes towards alcohol and road safety standards. However, it is necessary to conduct a more accurate study using multivariate analysis to define specific factors influencing young population's decision-making regarding road safety behavior."
Chen, X, Yang, J, Peek-Asa, C, Chen, K, Liu, X, & Li, L [31]	2014	English (China)	Prospective experimental case -control study	Low	Education	2 months	Mothers of newborn children, in hospital China	216 (114 intervention, 102 control)	Knowledge of child safety restraint use	"This study evaluates a hospital-based education intervention to promote child safety restraint use, especially in infants. The program improved the birthing mothers’ knowledge and awareness, which could drive them to prepare CSS for their babies. This study has implications for future comprehensive intervention strategies that address specific age-related needs and promote car seat use among infants and children."
Dorigatti, AE, Jimenez, LS, Redondano, BR, Carvalho, RBD, Calderan, TRA, & Fraga, GP [32]	2014	English (Brazil)	Pretest-Posttest, one group	Moderate	Education	Not specified	High school students 14–18 in Brazil	Each hospital visit included a mean of 70 students. The complete questionnaire was answered by 1,025	Knowledge of alcohol use and safety devices (Pre and post intervention questionnaire about behavior, alcohol consumption and general knowledge about trauma and emergency)	"The emergence of prevention programs such as these enables a behavioral change in the participant population, especially when the programs are performed by a multidisciplinary team, who can discuss the subject from different points of view, each according to their area of expertise. The P.A.R.T.Y. program exists as an option to help young people identify the risks of not using safety equipment in traffic, as well as the negative effects of the combination of drinking and driving."
Ederer, DJ; Bui, TV; Parke, EM; Roehler, DR; Sidik, M; Florian, MJ; Kim, P; Sim, S; Ballesteros, M. [33]	2016	English (Cambodia)	Controlled trial (not randomized)	Low	Education and provision of safety devices	1–2 weeks; 10–12 weeks; end of school year	School children grade 1–5 in Cambodia	Nine intervention schools (with a total of 6721 students) and four control schools (with a total of 3031 students)	Increase in helmet use on motorcycles and bicycles (Observation)	"School-based helmet use programmes that combine helmet provision and road safety education might increase helmet use among children."
Erkoboni D, OZanne-Smith J, Rouxiang C, Winston FK [34]	2010	English (China)	Mixed methods, pretest-Posttest, one group	Moderate	Education	6 weeks	Parents of children 3–8 in China	n = 71 at baseline, n = 62 at 6-week follow up	Self- reported knowledge and use of child seat restraints (short survey instrument)	"This study shows the possibility of exporting US-designed prevention interventions dubbed into Mandarin without the need to alter their original context (in this case, an African American family in a US setting) into a Chinese context. Successful cultural translation involved ensuring that the behavioural antecedents targeted in the intervention (eg, barriers and benefits) were of relevance to the Chinese population."
Falavigna A, Medeiros GS, Cannabarro CT, Barazzetti DO, Marcon G, Montiero CMC, Bossardi JB, Da Silva PG, Teles AR, Velho MC, Ferrari P. [35]	2014	English (Brazil)	RCT	Low	Education	1,3, and 8 months	Primary and high school students in Brazil	535 students	Self-reported knowledge of prevention of neurotrauma and use of safety devices (Questionnaires)	"Multiple and different types of educational interventions, such as lectures, scenes from plays about trauma and its consequences, traffic and fire department intervention, and medical emergency intervention directed to preteens and adolescents from public and private schools did not modify most students’ attitudes toward injury prevention."
Falavigna, A, Teles AR, Velho MC, Medeiros GS, Canabarro CT, de Braga GL, Barazzetti DO, Vedana VM, Kleber FD [36]	2012	English (Brazil)	RCT	Low	Education	5 months	High school students in Brazil	1049 students (5 intervention n = 572, 5 control schools n = 477)	Self-reported knowledge of prevention of neuro- trauma and use of safety devices (Questionnaires)	"An educational intervention based on a single lecture improved students’ knowledge of traumatic brain and spinal cord injuries, but this type of intervention did not modify most attitudes toward injury prevention."
FOROUTAN, A., HEYDARI, S. T., KARVAR, M., MOHAMMADI, L., SARIKHANI, Y., AKBARI, M. & LANKARANI, K. B. [37]	2019	English (Iran)	pre-post intervention with control group	low	Education, provision of safety devices, community awareness, legislation/law enforcement	9 months (can you double check that?)	Motorcyclists (adolescents subgroup) in two cities (intervention and control)	396 students	pre-intervention questionnaire, ICU admission rates, hospital costs for patients who required ICU admission, rate of helmet usage, mortality and the duration of ICU care for patients admitted to Darab hospital due to motorcycle accidents (Questionnaire, ICU data)	Even a short period of intervention can have positive effects on increasing the safety of motorcycle drivers.
Frandoloso, V., da Silva, F. T., & Magnabosco, C. D. [38]	2015	Portuguese (Brazil)	Longitudinal, observational cohort study	Moderate	Education	0–9 months	Children 9–11 in Brazil	117 children	Knowledge of prevention of traumatic brain injury (Standardized questionnaires)	"The high rate of experience with TBI coupled with the significant discrepancy between habits and knowledge regarding trauma prevention stress the need for effective measures leading to their actual implementation. The intervention increased awareness about the importance of helmet usage, suggesting partial effectivity from a theoretical standpoint."
FREITAS, C., RODRIGUES, M. A., PARREIRA, P., SANTOS, A., LIMA, S., FONTES, V. S., FREITAS, J. P. A., SANTOS, J. M. J. & MOTA, E. C. H. [39]	2019	English (Brazil)	Pretest—posttest with control group	low	Education	1 month	Children from 3rd to 5th grade in two public schools in Northeastern Brazil	173 children	Knowledge, attitudes and preventive practices of traffic accidents (KAP questionnaire)	"The educational intervention increased the level of knowledge and maintained the preventive attitudes and practices on traffic accidents at the same level in 3rd-5^th^ grade students."
Hidalgo- Solórzano, E., Híjar, M., Mora-Flores, G., Treviño-Siller, S., & Inclán-Valadez, C. [40]	2008	Spanish (Mexico)	Pretest-Posttest, one group	Low	Community campaign	Not specified	Children 16–19 in Mexico	700 children	Knowledge of RTI prevention methods (Self-applied questionnaire)	“Educative interventions represent an initial strategy for changes in knowledge and population behaviours. The present study offers an appropriate methodology to measure short-term changes in knowledge about risk factors associated with a significant problem affecting Mexican youth.”
Hijar M, Perez-Nunez R, Santoyo-Castillo D, Lunnen JC, Chandran A, Celis A, Carmona- Lozano S [41]	2013	English (Mexico)	Cross-sectional	Low	Community campaign and law enforcement and education	N/A	Children 13–18 years old in Mexico	5115 total, 13–15 years old n = 617, 16–18 years n = 2252	Knowledge and attitude scores (KAS) (Self-applied questionnaire)	“Our results show a potential moderate impact, measured as self-reported attitude change, resulting from the three intervention approaches under study. Future studies should address the intensity of exposure as well as the translation of attitude change into safer behaviors. Information generated by this study could be useful for local authorities in the intervention areas to inform their activities.”
Ji, Y., Ye, Y., Lu, Y., Li, L., & Yang, G [42]	2017	English (China)	Cluster RCT	Low	Education	Not specified	Seventh grade students in China	1312 students in intervention group	Knowledge and attitudes of prevention of RTI (Questionnaire)	“Publicity and education intervention measures have certain short-term effects on the prevention of bicycle injuries among rural middle school students; we should approach intervention measures according to the characteristics of traffic injuries in different areas.”
Jin, H. Q., Yingchun Li, Zhang, S. L., & Yu, W. S. [43]	2009	Chinese (China)	Cluster RCT	Low	Education	6 months	Middle school students in China	6784 (intervention) 1266 (control)	Incidence of bicycle injuries. (Survey)	“Program on road safety education significantly improved the relative knowledge for middle school student and it exerted positive effects in road safety attitude to some extent. However, no significant effect was found in the improvement on their behavior. Education on road safety should be carried out in the early stage of childhood with newer and more effective intervention approaches.”
Li, Z.-Y.; Zhang, Y.Y.; Huang, H.T. [44]	2011	Chinese (China)	Cluster RCT	Low	Education	1 year	Students in 7th-11th grade in China	1823 (interevention) 2306 (control)	Knowledge of RTI prevention and frequency of traffic rule violations (Injury reports, injury knowledge survey)	“The intervention measures of health education, institutionalized management, strict enforcement, environmental improvement can prevent and control the occurrence of bicycle injury among middle school students.”
Liu, X., Yang, J., Cheng, F., & Li, L. [45]	2016	English (China)	Cohort study, with control group	Moderate	Education and provision of safety devices	Not specified	Parents of newborns in China	Not reported	Knowledge of RTI prevention and self-reported use of safety devices (Interview via telephone)	“Education on safety, combined with a free CSS and professional installation training, were effective at increasing newborn parents’ knowledge and use of CSS. Future studies with larger sample sizes and longer follow-up are needed to determine a long-term effect of the intervention.”
Muguku, E., Ouma, J., & Yitambe, A. [46]	2010	English (Kenya)	Retrospective pretest-posttest	Low	Law enforcement	0–12 months	Children <18 in Kenya	Not reported	Number of hospital admissions due to RTI (Hospital admission records)	"The enforcement of the Traffic Act did not have any effect on injury severity among admitted PSV crash victims. Measures to lessen the burden of road traffic injury deserve greater attention."
Mutto, M., Kobusingye, O. C., & Lett, R. R [47]	2002	English (Uganda)	Retrospective, cross-sectional, observational	Moderate	Environmental change	N/A	Children in Uganda	13,064 pedestrians	Use of pedestrian overpass and incidence of fatal and non-fatal crashes (Injury records, observation)	“The prevalence of pedestrian overpass use was low with adult males least likely to use it. Pedestrians had a high perception of risk, which did not seem to influence overpass use. Pedestrian were more likely to be injured during slow traffic flows. There were more traffic crashes, and pedestrian injuries, but fewer fatalities after the construction of the overpass.”
Nazif-munoz, J. I., Nandi, A. & Ruiz-casares, M. [48]	2018	English (Brazil)	Evaluation study with interrupted time series design	Low	Legislation	Not specified	Children who were injured or died in vehicle collisions in Brazil between 2008 and 2014	Not reported	Number of child deaths and number of children injured in traffic collisions per child population, stratified by race (Various nation wide databases and census data)	“Socially advantaged populations were more likely to consistently adopt and employ restraint devices following the reform. Countries should also consider complementary policies that facilitate an equitable distribution of safety devices that reach vulnerable populations.”
Nazif-munoz, J. I. & Nikolic, N [49]	2018	English (Serbia)	Evaluation study with interrupted time series design	Low	Legislation	N/A	Child occupants aged 0–12	Not reported	Injury incidence pre and post intervention (Road Traffic Crashes Database by Serbian Road Traffic Safety Agency)	“The case of Serbia suggests that the new law was effective in reducing injuries among children aged 0–3 in the short term and injuries among children aged 4–12 in both the short term and long term.”
Poswayo, A., Kalolo, S., Rabonovitz, K., Witte, J. & Guerrero, A [50]	2019	English (Tanzania)	Pretest-Posttest, with control group	Low	Education, environmental change	1 year	Households around 18 primary schools in Dar es Salaam	12 957 school-aged children in the baseline period and 13 555 school-aged children in the post-intervention period	Injury Rates (Survey)	“The programme demonstrated a significant reduction in paediatric RTI after its implementation, in very specific ways. This study demonstrates that for a reasonable investment, scientifically driven injury prevention programmes are feasible in resource-limited settings with high paediatric RTI rates.”
Rimal, R. N., Yilma, H., Ryskulova, N. & Geber, S [51]	2019	English (Serbia)	Pretest-Posttest, with one group	Moderate	Education	6 months	Male and female adolescents, school-based	Before the intervention (N = 1449); Follow up: (N = 1072)	Change in risk perception (In-classroom filled out surveys)	“In order to reach male adolescents, who are at highest risk for automobile crashes and who have remained the most impervious to intervention effects, our findings suggest adopting an approach that improves their injunctive norms and, subsequently, exposes them to the safe-driving intervention.”
Salvarani, C.P., Colli, B.O., & Carlotti Junior, C.G [52]	2009	English (Brazil)	Pretest-Posttest, one group	Low	Community campaign	1 year	Adolescents in Brazil	Not reported	Number and severity of road traffic accidents (hospital record data and extra-hospital data)	"The adapted Think First was systematically implemented and its impact measured for the first time in Brazil, revealing the usefulness of the program for reducing trauma and TBI severity in traffic accidents through public education and representing a standardized model of implementation in a developing country."
Setyowati, D. L., Risva, Anwar A. [53]	2019	English (Indonesia)	pretest- posttest, one group	high	Education	Not specified	High school seniors in Indonesia	25 students	Knowledge and attitude of safe riding practices for motorcycles (Questionnaire)	"The training would increase the knowledge about safety riding to the Safety Riding Ambassadors."
Treviño-Siller, S., Pacheco-Magaña, L.E., Bonilla-Fernández, P., Rueda-Neria, C., & Arenas-Monreal, L. [54]	2017	English (Mexico)	Mixed methods, pretest-posttest	Moderate	Education	5 months	Students aged 10–15 years in Mexico	219 students	Knowledge and attitude scores of prevention of RTI (Observation, questionnaire)	“Because safe practices depend not only on children and youth but on the adults and social environment surrounding them, it is essential to engage parents, teachers, and decision makers in efforts to reduce RTIs. This will improve the establishment of commitments to impact social reality through consistent changes and mobilize greater resources for creating more secure communities in matters of road safety.”
Zare, H., Niknami, S., Heidarnia, A. & Hossein Fallah, M [55]	2019	English (Iran)	RCT	Low	Education, skills-based education	6 months	Two all- male elementary schools in Mehriz City, Iran	103 students	Rates of safe street-crossing behaviors (Observation)	“The results of the present study confirmed the positive effects of an active learning-based educational program with parental involvement on promoting safe street-crossing behaviors in 7-year-old children. Parental involvement is recommended as a useful strategy to consider while designing educational programs aiming at promoting positive street-crossing behaviors among school-aged children.”
Zimmerman, K., Jinadasa, D., Maegga, B., & Guerrero, A [56]	2015	English (Tanzania)	Pretest-Posttest, with control group	Low	Skills based education and provision of safety devices	9 months	Local Communities in Tanzania	Control n = 1,343, Intervention n = 2203	Incidence of RTI (Household survey)	"The incidence of RTIs in the low-volume rural setting is unacceptably high and most commonly associated with motorcycles. The change in incidence is unreliable due to logistic restraints of the project and more research is needed to quantify the impact of various RTI prevention strategies in this setting. This study provides insight into road traffic injuries on low-volume rural roads, areas where very little research has been captured. Additionally, it provides a replicable study design for those interested in collecting similar data on low-volume rural roads."
***Drowning***										
Callaghan JA, Hyder AA, Khan R, Blum LS, Arifeen S, Baqui AH [57]	2010	English (Bangladesh)	Observational pilot study with 3 intervention arms	Moderate	Supervision and provision of safety devices	0–9 months	Households with 1–4 year old children in Bangladesh	343 to education only, 373 to door barrier, 326 to playpen; 472 households, 2694 observations	Percentage of devise usage (Observation)	“Households provided with supervision tools use them, and there are lower observations of children unprotected… Effectiveness trials are needed to establish the impact of these tools on under-five drowning-specific mortality rates."
Davoudi-Kiakalayeh, A, Mohammadi, R, Yousefzade-Chabok, S, & Jansson, B [58]	2013	English (Iran)	Observational pretest-Posttest, two groups	Low	Supervision, education, environmental change and community campaign	0–2 years	0–9 and 10–19 year old children in Iran	Not reported	Incidence of drowning case, fatal and non-fatal (forensic medicine system and death registry for fatal cases; weekly ambulance excursion reports for non-fatal cases)	Rreducing the risk of drowning is possible by raising community awareness, in partnership with relevant organizations."
Guo, Q [59]	2010	Chinese (China)	Quasi-experimental trial with control group	Low	Community campaign and education	1.5 years	Students grades 4–6 in China	3015 students	Incidence of injury rate and knowledge of injury prevention (Survey)	"The school-based health education on drowning prevention is effective to improve children's knowledge and decrease their risk behaviors"
Guo, Q., Ma, W., Xu, H., Nie, S., Xu, Y., Song, X., & Li, H [60]	2010	Chinese (China)	Pretest-posttest	Low	Education	1 year	Children in grades 3–5, 7–8, and 10–11 in China	8930 students	Rate of drownings and knowledge of drowning prevention (Pre and post intervention survey)	"Health education program could improve children’s perception on water safety and reduce their risk behaviors as well as on the incidence of non—fatal drowning in the rural areas."
Rahman, F., Bose, S., Linnan, M., Rahman, A., Mashreky, S., Haaland, B., & Finkelstein, E. [61]	2012	English (Bangladesh)	Retrospective cohort	Low	Skills-based education	4-year observation	Children aged 1–4 in Bangladesh	Anchal (daycare) n = 18 596 participants; swimming lessons. (SwimSafe), n = 79,421 participants	Mortality rates due to drowning (Demographic Surveillance System)	"Based on World Health Organization criteria, PRECISE is very cost-effective and should be considered for implementation in other areas where drowning is a significant problem."
Shen, J., Pang, S., & Schwebel, D. C. [62]	2016	English (China)	RCT	Low	Education	1 week	Third and fourth grade students in China	280 students (137 in intervention. Group)	Knowledge of prevention of drowning (Self-report questionnaires)	"The testimonial-based intervention’s efficacy appears promising, as it improved safety knowledge and simulated risk behaviors with water among rural Chinese children."
Solomon, R., Giganti, M. J., Weiner, A., & Akpinar-Elci, M. [63]	2013	English (Grenada)	Pretest-Posttest, one group	Moderate	Education	Not specified	Primary school students aged 5-12in Grenada	92 enrolled, 56 participated	Knowledge of drowning prevention (Graded assessment)	"The findings from this study suggested that implementation of such a programme is effective. With cultural modifications and outsourcing, we believe this adapted programme would be successful in Grenada and other similar settings."
Turgut, T., Yaman, M., & Turgut, A [64]	2016	English (Turkey)	Pretest-posttest, one group	Moderate	Education and skills-based education	Not specified	Children 10–14 years old in Turkey	476 children	Knowledge of prevention of drowning (series of pre-post test surveys)	"We conclude that such a water safety education programme can help increasing knowledge and safe life-saving skills of children."
Zhang, P. B., Chen, R. H., Deng, J. Y., Xu, B. R., & Hu, Y. F. [65]	2003	Chinese (China)	Cluster RCT	Low	Education	1 year	Parents of children aged 1–4 in China	370 parents	Mortality rates and knowledge of drowning prevention (Survey and community-level monitoring)	"Health education to parents is an effective intervening measure for prevention of accidental suffocation and drowning. The goal of health education should be to change inadequate behavior and dangerous environment in which unintentional injury is easily happened. The interviewing measures that not sleeping with their infants in the same beds and not trying infants in a candle with blanket, and putting up fence beside pools and rivers are feasible and practicable."
Zhu, Y., Feng, X., Li, H., Huang, Y., Chen, J., & Xu, G. [66]	2017	English (China)	RCT	Moderate	Education	No follow up	Children aged 9–17 in China	752 children from three schools in Jiangbei district; (n = 380) or control (n = 372).	Knowledge of drowning prevention (Questionnaire)	“Use of ‘geo-located’ information added value to the effectiveness of a drowning prevention poster for enhancing awareness of drowning hotspots among children of migrant workers.”
Zhu, Y. C., Hui, L. I., Huang, Y. Q., Ding, K., Zhou, Y. F., & Wang, H., et al. [67]	2016	Chinese (China)	Cluster RCT	Low	Environmental change, education and community campaign	Not specified	Children in grades 1–9 in China	7736 and 7730 students from 1st - 9th grade	Incidence rate of non-fatal drowning (Survey)	“The model of integrated drowning interventions, based on the ecological approach and initiated by Ningbo, was proven to be effective and worth popularizing.”
***Falls*, *Burns*, *Poisoning***										
Gimeniz-Paschoal SR, Pereira DM, Nascimento EM [68]	2009	English (Brazil)	Mixed methods, pretest-posttest, with control group	Moderate	Education	1 week	Families with children under 4 years old in Brazil	40 families	Knowledge of prevention of burn (Home interviews)	"It is concluded that the intervention carried out in this study favorably affected the increase of correct information declared about the subject."
Gimeniz-Paschoal, S. R., Nascimento, E. N., Pereira, D. M., & Carvalho, F. F. [69]	2007	Portuguese (Brazil)	Pretest-Posttest, one group	Moderate	Education	No follow up	Relatives of hospitalized children aged 0–15 in Brazil	n = 37	Knowledge of prevention of burn (Structured questionnaires)	"The education action showed a good informative potential, suggesting its usefulness in the hospital context. This action should be tested in other places, such as primary and secondary attention health units and educational institutions."
Heard JP, Latenser BA, Liao J [70]	2013	English (Zambia)	Pretest-Posttest, with control group	Moderate	Education	11 months	Elementary school students in Zambia	550 at first survey, 2197 at second, 312 at follow up	Knowledge of prevention of burns (10-question survey)	“This study represents one of the few reports on the effectiveness of a burn prevention program in an LMIC. Future epidemiological data will be needed from nearby healthcare facilities to determine whether this program decreased burn morbidity and mortality at the hospital level.”
Jetten P, Chamania S, van Tulder, M [71]	2011	English (India)	Pilot pretest-posttest, three groups	Moderate	Education and provision of safety devices	1·5 months	Families with children under 4 years in India	42 families, 34 received intervention	Knowledge and self-reported use of safety device (Questionnaires)	“The prevention program seems an effective method in the reduction of **burns** of young children. Additionally, most families were satisfied with the intervention and would like to use it for a longer period of time. However, a large study with multiple evaluation moments would be needed to provide evidence of the effectiveness of this prevention program.”
Kebriaee-Zadeh, J., Safaeian, L., Salami, S., Mashhadian, F., & Sadeghian, G. H. [72]	2014	English (Iran)	Pretest-Posttest, one group	Low	Education	1 month	Students 10–11 years old in Iran	520 students	Knowledge of prevention of poisonings (Questionnaire)	"The school-based educational programs provide a good opportunity to poison information centers in preventing poisoning."
Konradsen, F., Pieris, R., Weerasinghe, M., van der Hoek, W., Eddleston, M., & Dawson, A. H [73]	2007	English (Sri Lanka)	Pretest-Posttest, one group	Moderate	Provision of safety devices	7 months	Households with children in Sri Lanka	172 households at follow up	Usage of safety devices (Questionnaire)	"The farming community appreciated the storage boxes and made storage of pesticides safer, especially for children. It seems that additional, intensive promotion is needed to ensure that pesticide boxes are locked. The introduction of in-house safe storage boxes resulted in a shift of storage into the farmer's home and away from the field and this may increase the domestic risk of impulsive self-poisoning episodes. This increased risk needs attention in future safe storage promotion projects."
Krug, A., Ellis, J. B., Hay, I. T., Mokgabudi, N. F., & Robertson, J. [74]	1994	English (South Africa)	Pilot study, pretest-posttest, with control group	Low	Education and provision of safety devices	Not specified	Families with children under 5 years old in South Africa	20,000 CRCs distributed	Incidence of poisonings (Hospital and clinic records, semi-structured questionnaire)	“We recommend that paraffin be sold in CRCs, and suggestions are made for improving health education to prevent paraffin poisoning.”
Makhubalo, O., Schulman, D., Rode, H. & Cox, S. [75]	2018	English (South Africa)	Controlled Trial	High	Community Awareness and provision of safety devices	1 month	Households with children 1–76 months	50 caregivers	Acceptability of device (post intervention phone interview and questionnaire)	“All participants had informed neighbors about the Kettle Strap and burn safety. The participants were prepared to pay ZAR 44 for the complete apparatus. The Kettle Strap is an acceptable, affordable device to improve kettle safety in the home.”
Odendaal, W., van Niekerk, A., Jordaan, E., & Seedat, M. [76] *(burns, falls, poisonings)	2009	English (South Africa)	RCT	Low	Education and provision of safety devices	1 week	Households with children under 10 years old in South Africa	Baseline: 211, 91 control households, 101 intervention households analyzed	Knowledge of general safety practice and change in risk assessment index (Observations and questionnaires)	“This study confirmed that a multi-component HVP effectively reduced household hazards associated with electrical and paraffin appliances and poisoning among children in a low-income South African setting.”
Rehmani, R., & LeBlanc, J. C. [77] *(falls and poisoning)	2010	English (Pakistan)	Non-blinded randomized controlled trial design	Low	Education	6 months	Families with children 3 years and older in Pakistan	"340 families, 304 (90%) completed follow up"	Observed change in risk factors and knowledge and attitude scores of injury prevention methods (Observation and questionnaire)	"Our study demonstrates the effectiveness of an educational intervention aimed at improving the home safety practices of families with young children."
Schwebel, D. C., Swart, D., Simpson, J., Hui, S. K. A., & Hobe, P. [78]	2009	English (South Africa)	Case-control	Low	Education	4 weeks	Households with children under 18 in South Africa	206 households	Self- reported knowledge of risk of poisonings and observed safety behaviors (Assessment and home inspection)	"The intervention was successful. A train-the-trainers model might be an effective educational tool to reduce kerosene-related injury risk in low-income communities within low- and middle-income countries."
Sinha, I., Patel, A., Kim, F. S., Maccorkle, M. L., & Watkins, J. F. [79]	2011	English (India)	Pretest-Posttest	Moderate	Education	No follow up	Children aged 5–7 in India	n = 39	Knowledge of burn prevention (Administered tests)	"This study demonstrates that a comic book has value in teaching children about burn awareness. Comic books may be a cost-effective method as an outreach tool for children."
Swart, L., van Niekerk, A., Seedat, M., & Jordaan, E [80] *(burn, falls and poisoning)	2008	English (South Africa)	Cluster RCT	Low	Education and provision of safety devices	2 weeks	Households with children under 10 years old South Africa	baseline questionnaire 410 households, 189 households intervention included in analysis, 188 controls	Observation of risk factors (Household survey)	"Our findings suggest that home visits by trained lay workers who provide education, home inspection, and safety devices may contribute to child injury risk reduction in LMICs. However, the improvements in burn- and poisoning-related injury risk reduction over time between intervention and control groups were modest. Furthermore, no reduction in injury risks due to falls was noted."
***Non-specific/ All Injuries***										
Cao BL, Shi XQ, Qi YH, Hui Y, Yang HJ, Shi SP, Luo, LR, Zhang H, Wang X, Yang YP [82]	2015	English (China)	Cluster randomized trial	Low	Education	16 months	School children 8–16 in China	n = 2342, randomly divided into intervention and control	Knowledge and attitude scores (KAS) (Survey)	"The SFI multi-level education intervention could significantly increase KASs for accidental injuries, which should improve children’s prevention-related knowledge and attitudes about such injuries. Our results highlight a new intervention model of injury prevention among school-aged children."
Fonseca, E., de la Caridad, R., Mendoza Molina, A., Castillo Rivera, J. A., & Martínez Rodríguez, M. D. L. Á. [83]	2014	Spanish (Cuba)	Mixed methods, pretest-Posttest, one group	Moderate	Education	18 months	Parents of children 0–18 months in Cuba	39 families	Knowledge of potential household injuries (Observation, Questionnaire)	“When families are approached with simple and accessible instruments, and with a community group work it is possible to make favorable changes in terms of awareness of the problem in the same place where it emerged."
Hernández Sánchez, M., García Roche, R., Vinardell Espín, P., & Mercedes, R. E. [84]	2017	Spanish (Cuba)	Pretest-Posttest, one group	Moderate	Education	Not specified	Health workers and educators of adolescents in Cuba	n = 331	Knowledge of prevention of unintentional injury (Questionnaires)	“The training is useful since the knowledge about unintentional injuries and their prevention was increased rapidly, for their subsequent replication in the different areas of action.”
Kahriman, I. & Karadeniz, H [85]	2018	English (Turkey)	Pretest-Posttest, one group	Low	Education	Not specified	Mother with children 0–6	300 mothers	Awareness of prevention methods for pediatric injuries (33-item questionnaire)	“The training provided to mothers to prevent pediatric injuries was effective in improving the awareness of the mothers.”
Khatlani, K., Alonge, O., Rahman, A., Hoque, D. M. E., Bhuiyan, A. A., Agrawal, P., & Rahman, F. [86]	2017	English (Bangladesh)	Nested, matched, case-control study	High	Supervision	One-year recall period	Caregivers of children under 5 years old in Bangladesh	504 (126 cases and 378 controls).	Mortality from unintentional injuries (Questionnaire, supervision)	“Children under five experiencing death due to unintentional injuries, including drowning, had 3.3 times increased odds of being unsupervised as compared with alive children (MOR = 3.3, 95% CI: 1.6–7.0), while adjusting for children’s sex, age, socioeconomic index, and adult caregivers’ age, education, occupation, and marital status. These findings are concerning and call for concerted, multi-sectoral efforts to design community-level prevention strategies. Public awareness and promotion of appropriate adult supervision strategies are needed.”
Liu, S., Luo, J., Xiang, B., Li, J., Yin, B., Zhu, K., Du, Y. [87]	2015	Chinese (China)	Cluster RCT	Low	Education and environment-tal change	No follow up	Students in grades 3–5 and 7–8 in China	pre-prevention: n = 1828; post-prevention: n = 1768 in total	Incidence of injury (Survey)	“Educational interventions can significantly reduce the incidence of injury among rural school-age children, and improve the cognitive level of children in rural school age to reduce the incidence of injury among rural school-age children in China.”
Mock, C., Arreola-Risa, C., Trevino-Perez, R., Almazan-Saavedra, V., Zozaya-Paz, J. E., Gonzalez-Solis, R.,… Hernandez-Torre, M. H. [88]	2003	English (Mexico)	Pretest-Posttest, with control group	Moderate	Education	4–6 months	Families with children aged 0–12 in Mexico	1124 children before counselling took place and on 625 after it had been given.	Knowledge about prevention of unintentional injuries (Questionnaires)	"Brief educational interventions targeting parents' practice of childhood safety improved safe behaviours. Increased attention should be given to specific safety-related devices and to the safety of pedestrians. Educational efforts should be combined with other strategies for injury prevention, such as the use of legislation and the improvement of environmental conditions."
Muñante-Nima, N., Majuan-López, K., & Farro-Peña, G [89]	2012	Spanish (Peru)	Pretest-Posttest, one group	Moderate	Education	1 week	Children 10–12 in Peru	72 children	Knowledge of unintentional injury prevention (Questionnaire)	The average knowledge level before the educational intervention was 12,46 points, to increase after intervention to 13,72 points, which can affirm, that the educational intervention was effective.
Muniz LAMA, Gonçalves Campos C, Caetano Romano MC, Pinto Braga P. [81]	**2020**	Portugese (Brazil)	post intervention, qualitative study	moderate	Education	1 week	state school students who work	19 students	knowledge about the risks of work accidents (Questionnaire and interview)	It is concluded that this work is important for adolescents, because it created the construction of a new knowledge about the risks of work accidents to which they may be exposed and thus, be able to make decisions about the care with their health.
NING, P., CHENG, P., SCHWEBEL, D. C., YANG, Y., YU, R., DENG, J., LI, S. & HU, G. 2019. [90]	2019	English (China)	Cluster RCT	low	Education	3 and 6 months	Caregivers of preschoolers aged 3–6 years from 20 preschools in Changsha, China	2920 caregivers	Unintentional injury incidences and caregivers’ self-reported attitudes and behaviors concerning child supervision (Online care-giver report)	"The app-based intervention did not reduce unintentional injury incidence among preschoolers but significantly improved caregivers’ safety behaviors. This app-based intervention approach to improve caregiver behaviors surrounding child injury risk offers promise to be modified and ultimately disseminated broadly."
Pérez, R. R. G., Pérez, N. T., & Martinez, M. U. [91]	2017	Spanish (Cuba)	Cohort Pretest-Posttest	Low	Education	6 months	Households with children under 5 years old in Cuba	112 households	Frequency of risk factors observed (Survey)	"It is considered that the communitarian intervention was successful. It is recommended the used classification of risk of the study and keep on performing interventions with this methodology."
Rahman, A.F., Rahman, A., Mashreky, S.R., & Linnan, M. [92]	2009	English (Bangladesh)	Pretest-Posttest, with control group	Low	Education and skills-based education	3 years	Children aged 0–17 in Bangladesh	The first three upazilas were chosen as intervention areas and the rest served as a control. In each upazila approximately 40,000 households comprising of about 200,000 population was covered.	Injury mortality and morbidity and knowledge of injury prevention methods (Baseline, ISS, and endline surveys, qualitative household interviews)	"The overarching conclusion is that child injury prevention works in rural Bangladesh. For the first time, there is evidence that injury, a leading cause of child death and serious morbidity in an LMIC such as Bangladesh can be prevented with the same reductions seen in the classical child survival interventions such as immunizations, breastfeeding and micronutrient supplementation."
Silva, F. B. E., Gondim, E. C., Henrique, N. C. P., Fonseca, L. M. M. & Mello, D. F. D. [93]	2018	Portugese (Brazil)	Pretest-Posttest, with one group	High	Education	5 months	Mothers aged 16–25 with children <3 years old	20 mothers	Mother’s knowledge of health education and prevention of injury (Graded assessment)	“The acquisition of knowledge of mothers points out that educational intervention through games is a satisfactory strategy in health education on child health care. However, the results suggest the importance of continuing educational actions at various times and contexts to ensure the sustainability of knowledge and practices, contributing to the integrality of health care.”
Tan, L.Z.; Peng, A.A.; Chen, Z.; Chen, J.; Guo, D.; Zhang, B. [94]	2012	Chinese (China)	Pretest-posttest	Moderate	Education	6 months	Parents of children aged 3–6 in China	181 children with their parents	Knowledge of injury prevention (Survey)	"Health education can significantly improve cognitive and behavioral of children and their parents on unintentional injuries."
Waisman, I., Rodriguez, M. I., Malamud, B., Zabala, R., Echegaray, L., & Bornoroni, G. E. [95]	2005	Spanish (Argentina)	Pretest-posttest	Moderate	Education	5 months	Mothers of children 1 month old in Argentina	205 mothers enrolled, 144 completed survey	Knowledge of accident and injury prevention (Survey)	"1) The educational program contributed to improve the risk and accident prevention knowledge and behaviors in the studied population. 2) Changes were more significant in the group of mothers who initially had the lowest level of information. 3) The areas with greatest difficulties were surveillance behaviors and unsafe behaviors related with electrical accidents prevention and use of baby car seats."
Wang, H., Liu, Y. X., Deng, W. J., Yang, W. J., & Wang, F. [96]	2015	English (China)	Case-Control	Low	Education	1 year	Families with children in kindergarten in China	2271 children, 904 intervention and 1367 control	Injury incidence rate (Household survey)	“Injury interventions can effectively prevent and control the occurrence of injury.”
Wang, X., Zhang, H., He, H., Ma, H. [97]	2008	Chinese (China)	Cluster RCT	Low	Education, community campaign and skills-based education	1month	Kindergarten students in China	Not reported (12 kindergartens)	Incidence rate and severity of injury (Survey, medical diagnosis)	“The interventions can significantly reduce the incidence rate of unintentional injuries of before-school-age children.”
Wang, X., & Zhu, Y. [98]	2009	Chinese (China)	Cluster RCT	Low	Education	Not specified	7th -12th grade students aged 12–18 in China	1236 (intervent.), 1320 (control)	Knowledge of accidental injury prevention and rates of injury (including RTI specific outcomes) (Survey, medical diagnosis)	"Peer education plays a important role on preventing accidental injuries in the middle school students."
Xiao, Z.H. [99]	2013	Chinese (China)	Cluster RCT	Moderate	Education	1 year	Parents with children in kindergarten in China	189 children and parents (intervention), 167 (control)	Knowledge of unintentional injury prevention and injury rate (Survey)	“Health education is an effective, rapid and economic intervention to reduce the incidence of unintentional injuries in children”.
Zhao, C.-H., Qiu, H.-S., & Qiu, H.-X. [100]	2006	Chinese (China)	Cluster RCT	Low	Education	1 year; 2 years	Parents of elementary school children in China	5727 parents	Incidence of injury rate (Survey, hospital records)	"Injury prevention strategies and child and parent safety education can reduce risks of accidental injury in children."
Zhou X. [101]	2013	Chinese (China)	Pretest-posttest	Moderate	Education and skills-based education	1 year	Parents and teachers of kindergarten students aged 3–6 in China	62,922 children registered at 182 kindergartens	Incidence rate of injury (Self-administered questionnaire)	“Health education interventions to reduce the occurrence of accidental injury in children are effective and feasible. Children accidental injuries are controllable. In different regions, child-care workers should take the corresponding health education interventions to reduce the incidence of children accidental injury according to the regional situation."

Although we only included studies conducted in LMIC, the geographic regions of first authors included Argentina, Bangladesh, Brazil, China, Colombia, Cuba, Denmark, Germany, Indonesia, Iran, Kenya, Mexico, The Netherlands, Pakistan, Peru, Saudi Arabia, South Africa, Sweden, Uganda, the United Kingdom, the United Republic of Tanzania, the United States, and the West Indies. The LMIC countries in which the studies were conducted included Argentina, Bangladesh, Brazil, Cambodia, China, Colombia, Cuba, Grenada, India, Indonesia, Iran, Kenya, Mexico, Pakistan, Peru, Serbia, South Africa, Sri Lanka, Turkey, Uganda, the United Republic of Tanzania, and Zambia.

The studies were classified as having low (n = 39), moderate (n = 29), or high (n = 6) risk of bias (Table 1, S1 Appendix).

**Fig 2:**
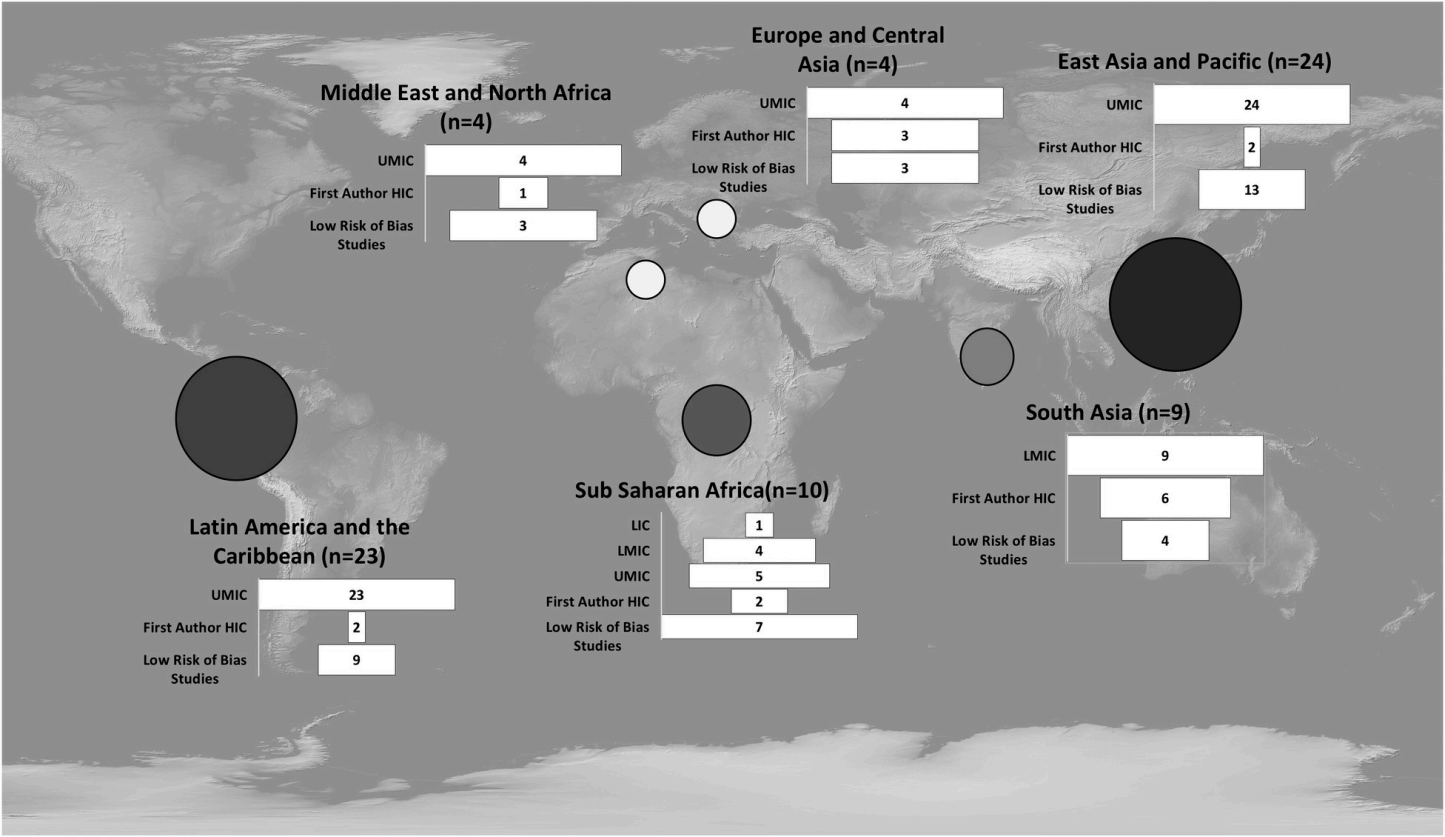
Study characteristics by location. SOURCE: Adapted from the public domain *Natural Earth* (https://www.naturalearthdata.com/downloads/10m-raster-data/10m-gray-earth/). NOTE: HIC = high income country, UMIC = upper middle income country, LMIC = lower middle income country, LIC = low income country.

The study characteristics, including the geographic regions of the interventions, the risk of bias, and the location of the first authors are shown in Fig 2.

### Qualitative summary of results

**Fig 3:**
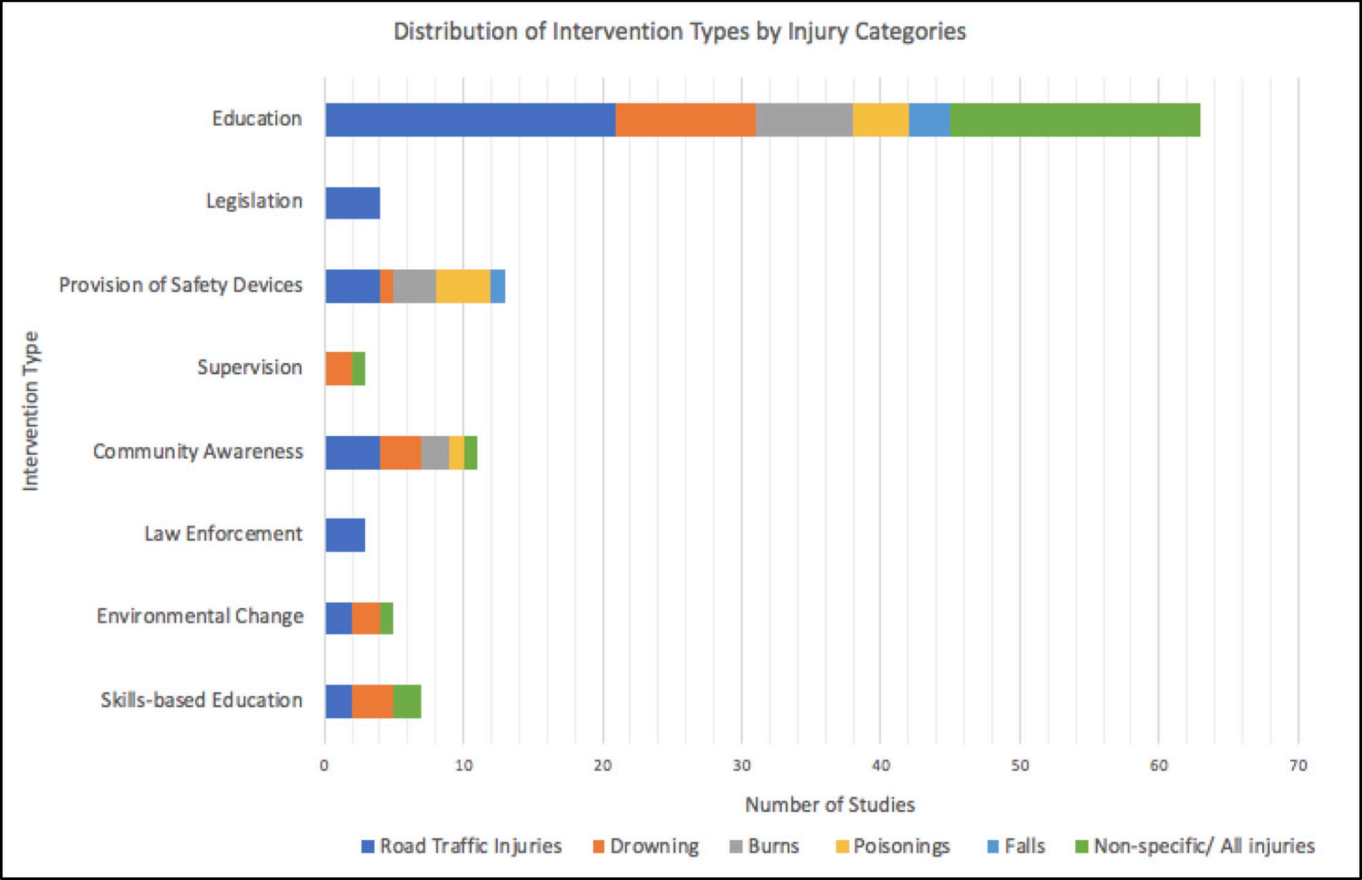
Distribution of intervention types by injury categories.

In our review, 30 studies dealt with RTIs, 11 with drowning, 8 with burns, 3 with falls, 7 with poisonings, and 21 did not specify the injury type (Fig 3). Some studies addressed multiple injury types or used more than one intervention category. A widely used framework to reduce accidents and unintentional injuries is the 5 E’s–engineering, education, encouragement, enforcement, and evaluation [1]. We built upon the 5 E framework by classifying interventions as one or more of the following categories Education interventions were classified as skills-based education (e.g. driving course, swimming lessons) and theory-based education and provision of information (e.g. lectures, videos, pamphlets without practical skill-based component). Engineering interventions. included environmental change (e.g. pedestrian overpass, water barriers) and the provision of safety devices (e.g. helmets, pesticide storage boxes). Enforcement interventions included law enforcement (e.g. roadside sobriety checks) and legislation (e.g. new laws). Finally, Encouragement interventions included community campaigns or awareness programs (e.g. television or radio messages) as well as supervision (e.g. observing adult presence).

Many studies assessed more than one intervention type. Seven studies used skills-based educational interventions, 5 changed environmental factors, 3 examined the effectiveness of law enforcement, 11 raised community awareness, 3 observed adult supervision as an intervention, 14 provided safety devices, 3 evaluated legislation, and 65 studies used an educational intervention. The follow-up time after each intervention ranged from 0 to 3 years, with most interventions (n = 42) having a follow-up period of less than 6 months, no follow-up at all, or not specified (Table 1).

A summary of the outcomes of these interventions, stratified by injury type, can be found in Tables 2–6.

**Table 2:** Summary of intervention outcomes for injury types: Road traffic injuries.

Intervention Type (Component of the 5 E Framework)	Intervention Description	Summary of Outcomes	
		Injury-Related Outcomes	Other Outcomes
*Road Traffic Injuries*			
Skills-based education (Education)	One-week driving course	Increase in RTIs perhaps due to changing climate conditions [56]	
	Practical training of safe street-crossing behaviors		Observed improvement of safe street crossing behaviors [55]
Environmental change (Engineering)	A pedestrian overpass was constructed	Fewer fatalities but more pedestrian injuries and traffic crashes [47].	
	Infrastructure enhancements designed to lower vehicle speeds and separate pedestrians from traffic	Reduced incidence of RTI [50]	
Law enforcement (Enforcement)	Additional sobriety checkpoints		Increased awareness of road safety measures [41]
	Confiscation of motorcycle for riders not wearing helmets	Decrease in hospital admissions, decrease in ICU admission due to head trauma (significant) increase in helmet use (significant), decreased mortality (not significant) (combined with other interventions) [37]	
	Traffic Act that increased arrests and surveillance	No changes in injury severity pre- and post-enforcement [46]	
Community awareness (Encouragement)	Radio messages, banners and posters	Decrease in hospital admissions, decrease in ICU admission due to head trauma (significant) increase in helmet use (significant), decreased mortality (not significant) (combined with other interventions) [37]	Increase in level of knowledge [40].
	Social marketing campaign		Reported awareness of road safety messages [41]
	Media resources, videos, t-shirts	Reduction in injury severity, mainly for traumatic brain injuries [52]	
Provision of safety devices. (Engineering)	Provision of motorcycle and bicycle helmets	Decrease in hospital admissions, decrease in ICU admission due to head trauma (significant) increase in helmet use (significant), decreased mortality (not significant) (combined with other interventions) [37]	Increased observed helmet use [33]
	Provision of child safety seats		Increased self-reported use of child safety seats [45]
	Provision of reflector vests and motorcycle helmets	Increase in RTIs perhaps due to changing climate conditions [56]	
Legislation (Enforcement)	Zero blood-alcohol limit and higher penalties for drinking and driving	Decrease in overall and pedestrian mortality [28]	
	Law requiring helmets be worn on motorcycles	Decrease in hospital admissions, decrease in ICU admission due to head trauma (significant) increase in helmet use (significant), decreased mortality (not significant) (combined with other interventions) [37]	
	Child restraint legislation	Reduction in the rate of child injuries [48]	
Education (Education)	Storybooks		Increase in knowledge of road safety [29].
	Pamphlets and videos for child safety seats		Increase in knowledge and purchase of child safety seat [31]
	Lectures	Decrease in incidence of bicycle injuries [42]; decrease in hospital admissions, decrease in ICU admission due to head trauma (significant) increase in helmet use (significant), decreased mortality (not significant) (combined with other interventions) [37]	Increase in knowledge of drink driving risk; [32] change in knowledge about brain and spinal cord injuries but no change in attitudes toward prevention; [36] increased knowledge on helmet usage only; [38] no change in reported attitudes about injury prevention; [35] increase in knowledge and safety practices; [54] change in self-reported attitude; [41] decrease in self-reported drink driving; [30] reduced incidence of RTI; [50] change in risk perception [51]
	In-school training on the importance of helmets and proper fit		Increase in observed helmet use [33]
	Promotional videos		Increase in knowledge and self-reported use of booster seats [34]
	Education materials	No change in traffic violations or accidents; [43, 44]	Change in knowledge; [43] increase in knowledge; [44] education alone did not increase use of child safety seats; [45] observed improvement of safe street crossing behaviors [55]
	Lectures, posters and guidebooks	Decrease in incidence of injury [87]	
	Peer education		Increase in knowledge of safety [53]
	Educational program using the educational therapeutic method		Increased level of knowledge and maintained the preventive attitudes and practices of traffic accidents [39]
	Seminars and videos	Decrease in injury [96]	Increase in knowledge [96]

The outcomes reported may be a summary of more than one intervention type.

**Table 3:** Summary of intervention outcomes for injury types: Drowning.

Intervention Type (Component of the 5 E Framework)	Intervention Description	Summary of Outcomes	
		Injury-Related Outcomes	Other Outcomes
*Drowning*			
Skills-based education (Education)	Swimming lessons	Reduction in relative risk of drowning death [61]	
	Lifeline throwing skills		Increase in skill level [64]
Environmental Change (Engineering)	Elimination of water reservoirs	Reduced incidence of fatal drowning [58]	
	Addition of barriers to unsafe water areas	Decreased incidence rate of non-fatal drowning [67]	
Community Awareness (Encouragement)	Messages on local TV and radio stations	Decreased risk of drowning [58]	
	Radio messages and newsletters	No significant decrease in non-fatal drowning [59]	Increase in knowledge [59]
	Media collaboration with health department	Decrease in incidence of non-fatal drowning [67]	
Supervision (Encouragement)	Observed supervision		Higher percentage of children supervised [57]
	Increased life guard and rescue stations	Decrease in risk and probability of drowning [58]	
Provision of Safety Devices (Engineering)	Provision of door barrier and playpen		Tools accepted by parents in community [57]
Education (Education)	Training of community health workers	Decrease in risk of drowning [58]	
	Education materials	No change in non-fatal drowning incidence [59]	Increase in knowledge; [59] increase in knowledge; [60] increase in perception of drowning risk [66]
	Workshops		Increase in water safety knowledge; [63] increase in water safety knowledge [64]
	Video testimonials		Improved knowledge and simulated water behaviors [62]
	Lectures	Reduction in mortality rates; [65] decrease in incidence of non-fatal drowning [67]	Change in parental knowledge [65]

The outcomes reported may be a summary of more than one intervention type.

**Table 4:** Summary of intervention outcomes for injury types: Burns.

Intervention Type (Component of the 5 E Framework)	Intervention Description	Summary of Outcomes	
		Injury-Related Outcomes	Other Outcomes
*Burns*			
Community Awareness (Encouragement)	Training community workers to deliver materials		Increase in self-reported knowledge and observed safety practices [78]
	Encouraging neighbors to share information about burn safety		All participants had informed neighbors about the kettle strap and burn safety [75]
Provision of Safety Devices (Engineering)	Provision of door barrier and playpen	Decrease in burns [71]	
	Provision of insulation tape and safe nails for electrical cords	Electrical and paraffin hazards and burns decreased [76]	
	Provision of kettle strap		Device is acceptable and affordable [75]
Education (Education)	Educational materials		Increase in knowledge; [68] increase in knowledge and belief of prevention; [69] reduction in hazards [76]
	Presentation and coloring book		Increase and decrease in knowledge for different concepts [70]
	Informative film and verbal instruction	Decrease in burns [71]	
	Comic books		Increase in knowledge [79]
	Home education	No significant decline in injury risk [80]	

The outcomes reported may be a summary of more than one intervention type.

**Table 5:** Summary of intervention outcomes for injury types: Poisonings.

Intervention Type (Component of the 5 E Framework)	Intervention Description	Summary of Outcomes	
		Injury-Related Outcomes	Other Outcomes
*Poisonings*			
Community Awareness (Encouragement)	Training community workers to deliver materials		Increase in self-reported knowledge and observed safety practices [78]
Provision of Safety Devices (Engineering)	Child-proof, lockable storage containers	Decrease in incidence of paraffin ingestion [74]	Increase in pesticides kept safe from children; [73] reduction in poison hazards; [76] no significant decrease in hazards [80]
Education (Engineering)	Seminars		Increase in knowledge [72]
	Printed materials	Reduction in paraffin ingestion [74]	Reduction in hazards [76]
	Counselling		Observed change in risk factors and knowledge and attitude scores of injury prevention methods [77]
	Home education	No significant decline in injury risk [80]	

The outcomes reported may be a summary of more than one intervention type.

**Table 6:** Summary of intervention outcomes for injury types: Non-specific/all injuries.

Intervention Type (Component of the 5 E Framework)	Intervention Description	Summary of Outcomes	
		Injury-Related Outcomes	Other Outcomes
*Non-specific/ All injuries*			
Skills-based education (Education)	Emergency response training	Incidence rate and severity of injury decreased [97]	
	First aid training	Reduction in injury rate among boys [101]	Increase in caregiver knowledge [101]
Environmental change (Engineering)	Upgrade school environment	Reduction in incidence of injury [87]	
Community Awareness (Encouragement)	Change in community regulations	Decrease in incidence of injuries [97].	
Supervision (Encouragement)	Evaluation of supervision	Unintentional injury mortality 3x higher when unsupervised—mainly for drowning [86]	
Education (Education)	Posters, letters, lectures, videos		Increased knowledge [103]
	Training of health workers		Increase in knowledge [84]
	Lectures, posters, guidebooks	Decrease in injury incidence [87]	
	Counselling		Increase in parental knowledge and safety behaviors [88]
	Lectures	Decrease of injury rate; [99] decrease in injury incidence rates [96]	Increase in knowledge, decrease in risks in household; [83] increase in knowledge; [89] increase in knowledge; [99] increase in knowledge [94]
	Communication from doctor	Decrease in accidents [91]	Decrease in risk factors for injury [91]
	Education, conversation circles, dialogical relationship framework		Increased knowledge of risk factors [81]
	Brochures		Increase in knowledge [104]
	App-based parenting education	No change in unintentional injury incidence among preschoolers [90]	Significantly improved caregivers’ safety behaviors [90]
	Guidebooks, peer education	Decrease in incidence rate and severity of injury; [97] decrease in injury rate (including RTI-specific) [98]	Increase in knowledge [98]
	Games and posters	Decrease in the rate of injury (including RTI specific) [98]	Increase in injury prevention knowledge [93, 98]
	Education of parents, teachers, and children	Decrease in injury rate among boys [101]	Increase in knowledge among parents; [101] increase in mothers’ awareness of prevention methods for pediatric injuries [85]

The outcomes reported may be a summary of more than one intervention type

For RTIs, the studies used interventions in skills-based education, environmental change, law enforcement, community awareness, provision of safety devices, legislation, and education (Table 2). The majority of these studies found an increase in road safety knowledge and self-reported safety behaviors. All studies that reported injury related outcomes (incidence, risk, severity, mortality) showed a decrease in numbers, except one study from Tanzania [56] that reported a 3% increase in RTI incidence on the intervention site with no change in incidence at the control site.

For drowning, the studies used interventions in skills-based education, environmental change, community awareness, supervision, provision of safety devices, and education (Table 3). The majority of these studies found an increase in knowledge of the prevention of drowning. Most studies found a reduction in the incidence of non-fatal and fatal drowning [58, 67, 92]. One study did not find a significant decrease in injury rates (11.1% to 11.0%), but did find a positive increase in knowledge following a community awareness program and education materials [59].

For burns, the studies used interventions in the categories of community awareness, provision of safety devices, legislation, and education (Table 4). A reduction in the incidence of burns and hospitalizations as a result of burns was found following the provision of safety devices [71, 76]. While one study did not find statistically significant intervention effects on electrical and paraffin burn–related hazard reduction, they did report a significant change in burn-related safety practices and behaviors [80]. An increase in knowledge of the prevention of burns was reported in the studies using educational interventions [68–70, 75, 76, 78, 79].

In the fall category, the studies used interventions including the provision of safety devices and education, combined with interventions about poisonings and burns. There was no significant decline in hazards or injury risk [76, 77, 80].

For poisonings, the studies used interventions in the categories of community awareness, provision of safety devices, and education (Table 5). Most studies reported an increase in observed safety behaviors [73, 76, 78], and an increase in self-reported knowledge of prevention methods [72]. Krug et al. reported a decrease in the incidence by 47.4% of poisonings by ingestion after the distribution of child restraint containers for paraffin [74]. Swart (2008), who also provided safety devices and conducted several home visits, did not find a significant intervention effect for poisoning and falls, but for burn safety practices (p-value 0.021 intervention effect -0.41 (-0.76 to -0.07)) [80].

The intervention types used for non-specified injury categories included skills-based education, environmental change, community awareness, supervision, and education (Table 6). These studies mainly reported an increase in caregiver knowledge of injury prevention methods. Nine studies reported positive outcomes directly related to injury rates, incidence, mortality, and severity [86, 91, 92, 96, 97, 99, 100, 102].

Interestingly, 2 studies conducted in LICs evaluated implemented environmental changes, as recommended by the WHO, and found varying results. While infrastructure enhancements to reduce vehicle speed and create spatial separation from pedestrians and vehicles resulted in a reduction of injuries [50] the construction of a pedestrian overpass resulted in an increase of pedestrian injuries and traffic crashes, albeit reducing fatality rates [47].

**Fig 4:**
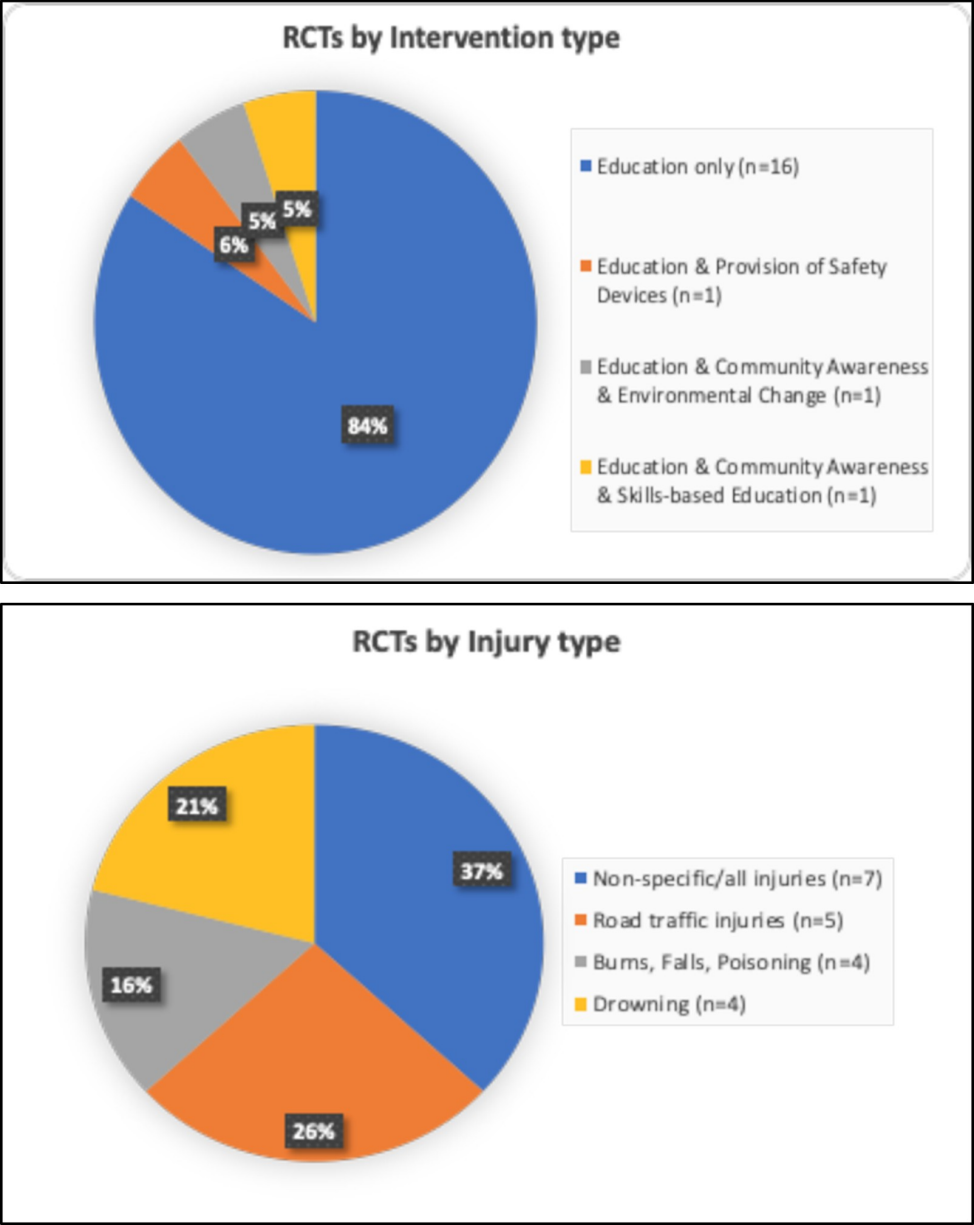
**a.** RCTs by intervention type. **b.** RCTs by injury type.

Among the 19 RCTs included, 84% (n = 16) used educational interventions, with the remaining studies using a combination of education and other interventions (Fig 4A).

Additionally, 26% (n = 5) of the RCTs addressed RTIs, 37% (n = 7) non-specific or all injury categories, 21% (n = 4) addressed drowning, and 16% (n = 4) examined burns, falls, or poisonings (Fig 4B).

## Discussion

This is the first systematic review evaluating all types of childhood unintentional injury prevention initiatives in LMICs published within the past 30 years, building on the 2008 WHO report on Child Injury Prevention. It is also the first to summarize the available evidence in English and non-English studies on LMIC childhood prevention interventions by injury, geographic location, and intervention type. This systematic review confirms that despite having the highest global burden of childhood unintentional injuries, LMICs have a disproportionately limited amount of research in this area compared to HICs [2, 18]. The findings reveal that 1) there is an unequal distribution of research regarding each injury type and a lack of injury-specific research, 2) there is an uneven geographical coverage, and 3) the general quality of the included studies was low, often due to the study design and the failure to be sensitive to or relevant for local cultures.

### Unequal distribution of injury types

The number of studies per injury type varied greatly, revealing an unequal distribution of research regarding each injury type. The highest burden of RTI in children is found in Africa, with 19.9 deaths per 100,000 people, followed by LMICs in the Eastern Mediterranean with 17.4 deaths per 100,000 people [105]. The majority of the studies focused on RTI, which may be warranted given their high burden among children in LMICs. However, we found that less than 15% of the included studies took place in SSA, representing only 3 out of the 48 SSA countries [46, 47, 50, 56]. Additionally, only one study was conducted in the Mediterranean [55], which demonstrates the need to focus future intervention programs on countries with the highest burden of RTI. The number of studies conducted for the other injury types were generally representative of the disease burden in the geographical locations. There is very little research on falls among children in LMICs, with the majority of this research targeted towards the elderly population in HIC [13]. Compared to other injury types, there is a low burden of fall injury among children in LMICs and this was consistent with the small number of studies focusing on falls included in this review. The included drowning prevention interventions were mostly conducted in Asian countries, where it is most prevalent, surpassing the highest reported injury rates for RTI (19.9 per 100,000 in Africa), with 30 per 100,000 in China, Philippines, Bangladesh, Vietnam, and Thailand. While the above findings show studies that mostly corresponded with the associated disease burden, 33% studies focused on “all” or “non-specific” injury types. The WHO recommends research specific actions to decrease the burden of childhood injuries, namely to “enhance the quality and quantity of data for child injury prevention” and “define priorities for research, and support research on the causes, consequences, costs and prevention of child injuries” [19].

### Uneven geographical coverage

The geographical distribution of the studies also reveals uneven coverage as seen in Fig 2. In terms of authorship, 16 of the 74 included articles had first authors from HIC, and 51 out of 74 had first authors from upper middle-income countries (UMICs), leaving only 7 articles with first authors from low andlower middle income countries. Therefore, conducting interventions in LMICs does not imply that local researchers were consulted or included in research design and application. 58 of the 74 (78%) included studies were conducted in 13 UMICs). However, 34 of the 58 (59%) were conducted in either Brazil or China. Latin America does not represent the highest burden of injury compared to Africa and Asia, yet is represented by 30% of the included studies, with more than half of the studies conducted in Brazil. While 22% of all UMICs are included in this review, 13% of all LMICs and only 6% of low-income countries (LICs) were the setting for childhood injury prevention program evaluations. LICs were not only the most underrepresented group, but also the least diverse group in terms of injury types and geographical locations, including only RTI interventions in SSA. While LICs face the highest injury burden [19], the limited number of interventions conducted could be due to a lack of available funding to invest in expensive prevention strategies that have been successful in HICs. However, in many LICs, the roads are shared by pedestrians, animals, cars, buses, motorcyclists, and bicyclists.

### Lack of high-quality study designs and relevance to local culture

Lastly, the quality of the included studies varied greatly, and our findings support the need for implementing high-quality interventions that are culturally sensitive, relevant, and welcomed by the local culture [106]. The insufficient funding for research on injury prevention in LMICs has resulted in few randomized evaluations and few studies with control groups or significant follow-up periods [107]. In this review, only 19 RCTs were included, and 47% (n = 35) of the 74 studies had a moderate or high risk of bias. In many studies, a self-reported survey tool was used to determine injury rates. Among the RCTs included, the majority used educational interventions and addressed all or non-specific injury categories, leading to a lack of clearly defined, injury-related outcomes. Importantly, 6 of the 9 RCTS since 2008 have been conducted in China almost exclusively investigating the impact of education on all or non-specific injuries within the school setting. We only included studies that included a comparison group to assess the impact of the prevention intervention. Rothman et al. recently conducted a review on the study designs used to assess the quality of child injury prevention interventions that were published between 2013 and 2016 [108]. Their findings also suggest a lack of high quality, hypothesis-driven study designs. The evaluation of all of the studies in this review mainly showed a positive or desired self-reported change following educational interventions, a need for longer follow-up, the need for effectiveness trials to access behavior change, and the need for an increase in injury prevention services in low- and middle-income settings. While those recommendations are critical and in line with what other organizations have called for, they do not provide a strong indication on which of those recommendations will be most important to focus on going forward. Beyond improved study designs, higher quality interventions will be sensitive to the needs of specific populations, environments, and available resources [106]. A validation study by Kohrt et al. also points out the risk of doing more harm than good by providing interventions that have not been validated in comparable settings [109]. For example, although overpasses have been effective in other settings, one study that implemented an overpass for a busy street saw an increase in RTI, due to individuals’ perceptions that the overpass increased walking distance and its low visibility created a new space for crime [47].

### Limitations

This review has certain limitations. Due to our inclusion of various methodologies, conducting a quantitative meta-analysis was not possible. There were 19 RCTs, but they reported non-similar outcomes for different injuries, and had varying study follow up times, making them not suitable for a meta-analysis. Additionally, including only a single type of study design would not have produced a thorough representation of the present literature on the prevention of unintentional injuries of children in LMICs. Our inclusion criteria included a control group and peer-review, which adds to the cost of the studies, excludes unpublished theses and non-indexed journals that are common in LMICs, and does not account for researchers falling victim to predatory journals that do not lead to publication. Grey literature was not included in our analysis, which could lead to possible exclusion of presented intervention programs, but we ensured that only data of sufficient rigor was included in this review by making peer-reviewed publication part of our inclusion criteria. In order to categorize the injury types, we have followed the major categories of injury presented by WHO. While this may have resulted in the exclusion of some other types of injury, such as animal bites or sunburns, those injury types, while important to address in specific hotspots and high-risk populations, do not account for a significant burden of injuries globally. Additionally, some of the reported injuries could have been a result of violence against children and falsely identified as unintentional, which could impact the effectiveness of the investigated prevention methods for unintentional injuries. We have included countries based on their current income status rather than at the time of the publication of the study to identify the needs and intervention possibilities for current LMICs. While this likely does not exclude any countries in the low-income category, we may have excluded a few countries that moved from UMIC to HIC over time.

### Conclusion

Childhood unintentional injury contributes to a significant amount of global mortality and morbidity. Children living in LMICs are especially vulnerable to injury. There have been numerous effective and low-cost solutions for injuries, but there is a lack of dispersal of these initiatives into the most affected settings. This requires significant political will and increased funding to go beyond educational initiatives with self-reported measures and little follow-up time, to robust research and interventions that will reduce the global burden of unintentional injuries among children. Low or non-existing funding is a significant and ongoing barrier for researchers from LMIC. International donors providing research funding should focus on LMIC, and particularly LIC, and provide assistance in writing grant proposals to allow for more rigorous study designs, thereby improving the quality of research relevant to LMIC. To significantly reduce the rates of injury and the associated negative health and social outcomes, future studies should focus on high-quality trials to assess targeted intervention strategies for areas with a high injury burden that are specifically tailored for the needs of specific cultural and geographical settings.

## Supporting information

S1 Appendix Data quality assessments. (DOCX)

S2 Appendix Search terms. (DOCX)

S3 Appendix PRISMA checklist. (DOCX)
