# Supplementary material for: Prevention of childhood unintentional injuries in low- and middle-income countries: A systematic review
Source: PLoS One. 2020 Dec 29;15(12):e0243464. doi: 10.1371/journal.pone.0243464 (PMC7771986; doi:10.1371/journal.pone.0243464)
Supplement: S1 Appendix — (DOCX) [file pone.0243464.s001.docx]

S1 Appendix. Data Quality Assessments.

A1. Cochrane RoB 2.0 Tool for All Included Articles.

| **Reference** | **Randomized Control Trial?** | **Cochrane RoB 2.0 Tool** | | | | | **Overall Bias (Low, Moderate, or High)** |
| --- | --- | --- | --- | --- | --- | --- | --- |
|  |  | **1. Bias arising from the randomization process** | **2. Bias due to deviations from intended interventions** | **3.Bias due to missing outcome data** | **4. Bias in measurement of the outcome** | **5. Bias in selection of the reported result** |  |
| Abreu DR de OM, Souza EM de, Mathias TA de F. [Impact of the Brazilian Traffic Code and the Law Against Drinking and Driving on mortality from motor vehicle accidents]. Cad Saude Publica. 2018 Aug 20;34(8):e00122117. | no | N/A | low | low | low | low | low |
| Ahmad H, Naeem R, Feroze A, Zia N, Shakoor A, Khan UR, et al. Teaching children road safety through storybooks: an approach to child health literacy in Pakistan. BMC Pediatr. 2018 Feb 7;18(1):31. | no | N/A | low | moderate | low | moderate | moderate |
| Callaghan JA, Hyder AA, Khan R, Blum LS, Arifeen S, Baqui AH. Child supervision practices for drowning prevention in rural Bangladesh: a pilot study of supervision tools. J Epidemiol Community Health. 2010 Jul;64(7):645–7. | no | low | low | moderate | low | low | moderate |
| Cao B-L, Shi X-Q, Qi Y-H, Hui Y, Yang H-J, Shi S-P, et al. Effect of a multi-level education intervention model on knowledge and attitudes of accidental injuries in rural children in Zunyi, Southwest China. Int J Environ Res Public Health. 2015 Apr 8;12(4):3903–14 | yes | moderate | low | low | low | low | low |
| Charry JD, Ochoa JD, Tejada JH, Navarro-Parra SL, Esquivel N, Vasques Y. Education in trauma: An educational alternative that promotes injury prevention. Chin J Traumatol. 2017 Oct;20(5):275–7.. | no | N/A | low | low | moderate | moderate | moderate |
| Chen X, Yang J, Peek-Asa C, Chen K, Liu X, Li L. Hospital-based program to increase child safety restraint use among birthing mothers in China. PLoS One. 2014 Aug 18;9(8):e105100. | no | low | low | moderate | low | low | low |
| Davoudi-Kiakalayeh A, Mohammadi R, Yousefzade-Chabok S, Jansson B. Evaluation of a community-based drowning prevention programme in northern Islamic Republic of Iran. East Mediterr Health J. 2013;19(7):629–37. | no | moderate | low | low | low | low | low |
| Dorigatti AE, Jimenez LS, Redondano BR, Carvalho RB de, Calderan TRA, Fraga GP. Importância de programa multiprofissional de prevenção de trauma para jovens. Revista do Colégio Brasileiro de Cirurgiões. 2014;41(4):245–50. | no | low | low | moderate | low | low | moderate |
| Ederer DJ, Bui TV, Parker EM, Roehler DR, Sidik M, Florian MJ, et al. Helmets for Kids: evaluation of a school-based helmet intervention in Cambodia. Inj Prev. 2016 Feb;22(1):52–8. | no | low | low | Low | low | moderate | low |
| Erkoboni D, Ozanne-Smith J, Rouxiang C, Winston FK. Cultural translation: acceptability and efficacy of a US-based injury prevention intervention in China. Inj Prev. 2010 Oct;16(5):296–301. | no | low | low | low | low | low | moderate |
| Falavigna A, Medeiros GS, Canabarro CT, Barazzetti DO, Marcon G, Carneiro Monteiro GM, et al. How can we teach them about neurotrauma prevention? Prospective and randomized “Pense Bem-Caxias do Sul” study with multiple interventions in preteens and adolescents. J Neurosurg Pediatr. 2014 Jul;14(1):94–100. | yes | low | low | moderate | moderate | low | moderate |
| Falavigna A, Teles AR, Velho MC, Medeiros GS, Canabarro CT, de Braga GL, et al. Impact of an injury prevention program on teenagers’ knowledge and attitudes: results of the Pense Bem-Caxias do Sul Project. J Neurosurg Pediatr. 2012 May;9(5):562–8. | yes | moderate | low | high | low | low | moderate |
| Fonseca E, de la Caridad R, Mendoza Molina A, Castillo Rivera JA, Martínez Rodríguez M de los Á. Intervención comunitaria para la prevención de accidentes en niños. Humanidades Médicas. 2014;14(2):423–41. | no | low | low | low | moderate | moderate | moderate |
| Foroutan A, Heydari ST, Karvar M, Mohammadi L, Sarikhani Y, Akbari M, et al. Results of a Campaign for Motorcycle Helmets Advocacy in a City in Southwest of Iran; A Population-Based Intervention Study. Bull Emerg Trauma. 2019 Oct;7(4):404–10. | no | NA | low | low | low | low | low |
| Frandoloso V, Magnab CD, Carvalho FTBG. O impacto de aulas expositivas (ministrada para crianças entre 09 e 11 anos) sobre o reconhecimento de situações de risco para ocorrência de TCE. Revista UNIPLAC. 2015;3(1). | no | low | low | moderate | low | low | moderate |
| Freitas CKAC, Rodrigues MA, Parreira PMSD, Santos ACFSD, Lima SVMA, Fontes VS, et al. Educational program for the promotion of knowledge, attitudes and preventive practices for children in relation to traffic accidents: experimental study. Rev Paul Pediatr. 2019 Jul 4;37(4):458–64. | no | low | low | low | low | low | low |
| Gimeniz-Paschoal SR, Pereira DM, Nascimento EN. Efect of an educative action on relatives’ knowledge about childhood burns at home. Rev Lat Am Enfermagem. 2009;17(3):341–6. | no | moderate | low | low | moderate | low | moderate |
| Gimeniz-Paschoal SR, Nascimento EN, Pereira DM, Carvalho FF. Ação educativa sobre queimaduras infantis para familiares de crianças hospitalizadas. Revista Paulista de Pediatria. 2007;25(4):331–6. | no | low | low | low | low | low | moderate |
| Guo QZ. 广东省连平县小学生溺水健康教育干预近期效果评价 - 中国优秀硕士学位论文全文数据库 [Effectevaluation of water safety education on children nonfatal drowning preventionin Lianping County, Guangdong Province]. 暨南大学. 2010; | no | low | low | low | low | low | low |
| Guo QZ, Ma W-J, Xu H-F, Nie S-P, Xu Y-J, Song X-L, et al. 农村中小学生溺水健康教育干预近期效果评价 [Evaluation on the health education program regarding prevention of non-fatal drowning among school-aged children in Lianping county, Guangdong province]. 中国流行病学杂志v. 2010;31(1):22–6. | no | N/A | low | low | moderate | low | low |
| Heard JP, Latenser BA, Liao J. Burn prevention in Zambia: a work in progress. J Burn Care Res. 2013 Dec;34(6):598–606. | no | low | low | low | low | low | moderate |
| Hernández Sánchez M, García Roche R, Vinardell Espín P, Torres Hernández M, Ravelo Elvirez M. Formación de promotores para la prevención de lesiones no intencionales en adolescentes en Cuba entre 2008 y 2012. Revista Cubana de Higiene y Epidemiología. 2017;55(1):24–33. | no | low | low | low | low | low | moderate |
| Hidalgo-Solórzano E, Híjar M, Mora-Flores G, Treviño-Siller S, Inclán-Valadez C. Accidentes de tránsito de vehículos de motor en la población joven: evaluación de una intervención educativa en Cuernavaca, Morelos. salud pública de méxico. 2008;50:s60–8. | no | low | low | moderate | low | low | low |
| Híjar M, Pérez-Núñez R, Santoyo-Castillo D, Lunnen JC, Chandran A, Celis A, et al. Attitude change in youths after being exposed to different road safety interventions in two Mexican cities. Injury. 2013 Dec;44 Suppl 4:S4–10. | no | low | low | low | low | low | low |
| Jetten P, Chamania S, van Tulder M. Evaluation of a community-based prevention program for domestic burns of young children in India. Burns. 2011 Feb;37(1):139–44. | no | low | low | high | low | low | moderate |
| Ji Y, Ye Y, Lu Y, Li L, Yang G. An Intervention to Reduce Bicycle Injuries among Middle School Students in Rural China. Int J Environ Res Public Health. 2017 Jun 26;14(7). | yes | low | low | low | low | low | low |
| Jin HQ, Li YC, Zhang SL, Yu WS. [Evaluation on the effects of education regarding road safety among middle school students]. Zhonghua Liu Xing Bing Xue Za Zhi. 2009;30(8):797–801. | yes | N/A | low | low | low | moderate | low |
| Kahriman IL, Karadeniz H. Effects of a Safety-Awareness-Promoting Program Targeting Mothers of Children Aged 0-6 Years to Prevent Pediatric Injuries in the Home Environment: Implications for Nurses. J Trauma Nurs. 2018;25(5):327–35. | no | low | low | low | low | moderate | low |
| Kebriaee-Zadeh J, Safaeian L, Salami S, Mashhadian F, Sadeghian G-H. A school-based education concerning poisoning prevention in Isfahan, Iran. J Educ Health Promot. 2014 Feb 21;3:5. | no | low | low | low | low | moderate | low |
| Khatlani K, Alonge O, Rahman A, Hoque DME, Bhuiyan A-A, Agrawal P, et al. Caregiver supervision practices and risk of childhood unintentional injury mortality in bangladesh. Int J Environ Res Public Health. 2017 May 11;14(5). | no | low | low | low | low | moderate | high |
| Konradsen F, Pieris R, Weerasinghe M, van der Hoek W, Eddleston M, Dawson AH. Community uptake of safe storage boxes to reduce self-poisoning from pesticides in rural Sri Lanka. BMC Public Health. 2007 Jan 26;7:13. | no | low | low | low | low | low | moderate |
| Krug A, Ellis JB, Hay IT, Mokgabudi NF, Robertson J. The impact of child-resistant containers on the incidence of paraffin (kerosene) ingestion in children. S Afr Med J. 1994;84(11):730–4. | no | low | low | low | low | low | low |
| Li ZY, Zhang YY, Huang HT. Effect evaluation on intervention for bicycle traffic injuries among the middle school students in Yangpu, Shanghai. Chin J Sch Health. 2011;32:1330–1. | yes | low | low | low | low | low | low |
| Liu S, Luo J, Xiang B, Li J, Yin B, Zhu K, et al. 汉川市农村学龄儿童伤害教育干预效果评价. 卫生职业教育. 2015;(2015 年 16):94–5, 96 | yes | low | low | moderate | moderate | low | moderate |
| Liu X, Yang J, Cheng F, Li L. Newborn parent based intervention to increase child safety seat use. International journal of environmental research and public health. 2016;13(8):777. | no | low | low | low | moderate | low | moderate |
| Makhubalo O, Schulman D, Rode H, Cox S. Acceptability and functionality of the “Kettle Strap”: An attempt to decrease kettle related burns in children. Burns. 2018;44(5):1361–5. | no | N/A | low | low | moderate | moderate | moderate |
| Mock C, Arreola-Risa C, Trevino-Perez R, Almazan-Saavedra V, Zozaya-Paz JE, Gonzalez-Solis R, et al. Injury prevention counselling to improve safety practices by parents in Mexico. Bull World Health Organ. 2003 Oct 14;81(8):591–8. | no | moderate | moderate | moderate | low | low | high |
| Muguku E, Ouma J, Yitambe A. Effects of enforcement of the traffic act on injury severity among patients admitted at the Rift Valley Provincial General Hospital, Nakuru. East Afr Med J. 2010;87(11). | no | low | low | low | low | low | low |
| Muñante-Nima N, Majuan-López K, Farro-Peña G. Efectividad de una intervención educativa en el nivel de conocimientos sobre prevención de riesgos físicos ante sismos en escolares de 10 a 12 años. Rev enferm herediana. 2012;5(1):42–9. | no | low | low | low | low | low | moderate |
| Muniz LAMA, Gonçalves Campos C, Caetano Romano MC, Pinto Braga P. Acidentes de trabalho: percepção do adolescente. Revenf. 2019 Jan 30;(36). | no | NA | low | low | low | moderate | moderate |
| Mutto M, Kobusingye OC, Lett RR. The effect of an overpass on pedestrian injuries on a major highway in Kampala - Uganda. Afr Health Sci. 2002 Dec;2(3):89–93. | no | low | moderate | low | moderate | low | moderate |
| Nazif-Munoz JI, Nikolic N. The effectiveness of child restraint and seat belt legislation in reducing child injuries: The case of Serbia. Traffic Inj Prev. 2018 Feb 28;19(sup1):S7–14. | no | N/A | low | low | low | low | low |
| Nazif-Muñoz JI, Nandi A, Ruiz-Casares M. Protecting only white children: the impact of child restraint legislation in Brazil. J Public Health. 2019 Jun 1;41(2):287–95. | no | N/A | low | low | low | low | low |
| Ning P, Cheng P, Schwebel DC, Yang Y, Yu R, Deng J, et al. An App-Based Intervention for Caregivers to Prevent Unintentional Injury Among Preschoolers: Cluster Randomized Controlled Trial. JMIR Mhealth Uhealth. 2019 Aug 9;7(8):e13519. | yes | low | low | low | low | low | low |
| Odendaal W, van Niekerk A, Jordaan E, Seedat M. The impact of a home visitation programme on household hazards associated with unintentional childhood injuries: a randomised controlled trial. Accident Analysis & Prevention. 2009;41(1):183–90. | yes | moderate | low | low | low | low | low |
| Pérez RRG, Pérez NT, Martinez MU. Intervención sobre factores de riesgo de accidentes y accidentes en niños menores de cinco años. Medimay. 2017;24(2):143–59. | no | low | low | low | low | low | low |
| Poswayo A, Kalolo S, Rabonovitz K, Witte J, Guerrero A. School Area Road Safety Assessment and Improvements (SARSAI) programme reduces road traffic injuries among children in Tanzania. Inj Prev. 2019;25(5):414–20. | no | N/A | low | low | low | low | low |
| Rahman A, Rahman A, Mashreky SR, Linnan M. Evaluation of PRECISE: A Comprehensive Child Injury Prevention Program in Bangladesh. The first Three Years (2006–2008) Bangladesh: Dhaka: Centre for Injury Prevention and Research, Bangladesh (CIPRB). 2009; | no | moderate | low | low | low | low | low |
| Rahman F, Bose S, Linnan M, Rahman A, Mashreky S, Haaland B, et al. Cost-effectiveness of an injury and drowning prevention program in Bangladesh. Pediatrics. 2012 Dec;130(6):e1621-8. | no | low | low | low | low | low | low |
| Rehmani R, LeBlanc JC. Home visits reduce the number of hazards for childhood home injuries in Karachi, Pakistan: a randomized controlled trial. Int J Emerg Med. 2010;3(4):333. | yes | low | low | low | moderate | low | low |
| Rimal RN, Yilma H, Ryskulova N, Geber S. Driven to succeed: Improving adolescents’ driving behaviors through a personal narrative-based psychosocial intervention in Serbia. Accid Anal Prev. 2019 Jan;122:172–80. | no | low | low | moderate | low | moderate | moderate |
| Salvarani CP, Colli BO, Carlotti Júnior CG. Impact of a program for the prevention of traffic accidents in a Southern Brazilian city: a model for implementation in a developing country. Surg Neurol. 2009 Jul;72(1):6–13; discussion 13. | no | low | low | low | low | moderate | low |
| Schwebel DC, Swart D, Simpson J, Hobe P, Hui S-KA. An intervention to reduce kerosene-related burns and poisonings in low-income South African communities. Health Psychol. 2009 Jul;28(4):493–500. | no | low | low | low | moderate | low | low |
| Setyowati DL, Risva, Anwar A. Duta safety riding: the actors of traffic accidents prevention in samarinda, east kalimantan, indonesia. Ind Jour of Publ Health Rese & Develop. 2019;10(11):1709. | no | N/A | low | low | high | high | high |
| Shen J, Pang S, Schwebel DC. Evaluation of a drowning prevention program based on testimonial videos: A randomized controlled trial. J Pediatr Psychol. 2016 Jun;41(5):555–65. | yes | low | low | low | low | low | low |
| Silva FB e, Gondim EC, Henrique NCP, Fonseca LMM, Mello DF de. Intervenção educativa com mães jovens: aquisição de saberes sobre cuidados da criança. Acta Paul Enferm (Online). 2018 Feb;31(1):32–8. | no | N/A | low | low | high | moderate | high |
| Sinha I, Patel A, Kim FS, MacCorkle ML, Watkins JF. Comic books can educate children about burn safety in developing countries. J Burn Care Res. 2011;32(4):e112–7. | no | low | low | low | low | low | low |
| Solomon R, Giganti MJ, Weiner A, Akpinar-Elci M. Water safety education among primary school children in Grenada. Int J Inj Contr Saf Promot. 2013;20(3):266–70. | no | low | low | high | low | low | moderate |
| Swart L, van Niekerk A, Seedat M, Jordaan E. Paraprofessional home visitation program to prevent childhood unintentional injuries in low-income communities: a cluster randomized controlled trial. Inj Prev. 2008 Jun;14(3):164–9. | yes | low | low | low | low | low | low |
| Tan LZ, Peng AA, Chen Z, Chen J, Guo D, Zhang B. The effect of health education to cognitive and behavioral of the kindergarten children and their parents on unintentional injuries. Mater Child Health Care Chin. 2012;27:5049–51. | no | N/A | low | moderate | moderate | high | high |
| Treviño-Siller S, Pacheco-Magaña LE, Bonilla-Fernández P, Rueda-Neria C, Arenas-Monreal L. An educational intervention in road safety among children and teenagers in Mexico. Traffic Inj Prev. 2017 Feb 17;18(2):164–70. | no | low | low | low | low | low | low |
| Turgut T, Yaman M, Turgut A. Educating children on water safety for drowning prevention. Soc Indic Res. 2016 Nov;129(2):787–801. | no | low | low | moderate | low | low | low |
| Waisman I, Rodríguez MI, Malamud B, Zabala R, Echegaray L, Bornoroni GE. Un proyecto para prevención de accidentes desde el consultorio del pediatra. Archivos argentinos de pediatría. 2005;103(1):23–30. | no | low | low | low | low | low | low |
| Wang H, Liu Y-X, Deng W-J, Yang W-J, Wang F. Case-Control Study of Injury Intervention for Preschool Children in Henggang, Shenzhen. Pediatr Emerg Care. 2015 Oct;31(10):708–10. | no | low | low | low | low | moderate | low |
| Wang X, Zhu Y. [Peer education’s effects on preventing accidental injuries in middle school students]. Wei Sheng Yan Jiu. 2009;38(4):449–51. | yes | moderate | low | moderate | low | low | moderate |
| Wang X, Zhang H, He H, Ma H. 社区 “5Es” 干预, 高危个体干预降低学龄前儿童意外损伤的效果研究. 中国儿童保健杂志. 2008;16(2):224–6. | yes | moderate | low | high | low | moderate | high |
| Xiao ZH. 健康教育对降低儿童意外伤害发生的作用的探讨 [To explore the effect of health education to reduce the occurrence of child injury effect.]. Guide Chin Med. 2013;11:795–796. | yes | low | low | moderate | low | low | low |
| Zare H, Niknami S, Heidarnia A, Hossein Fallah M. Traffic safety education for child pedestrians: A randomized controlled trial with active learning approach to develop street-crossing behaviors. Transportation Research Part F: Traffic Psychology and Behaviour. 2019 Jan;60:734–42. | no | moderate | low | moderate | moderate | low | moderate |
| Zhang PB, Chen RH, Deng JY, Xu BR, Hu YF. [Evaluation on intervening efficacy of health education on accidental suffocation and drowning of children aged 0 - 4 in countryside]. Zhonghua Er Ke Za Zhi. 2003;41(7):497–500. | yes | moderate | low | moderate | low | low | moderate |
| Zhao C-H, Qiu HS, Qiu HX. Interventions to prevent accidental injuries in children between 7 and 13 years of age. Zhongguo dang dai er ke za zhi= Chinese journal of contemporary pediatrics. 2006;8(4):331–3. | yes | moderate | low | moderate | low | low | moderate |
| Zhou X. 浙江中医药大学学报 [Intervention to Children Accidental Injury]. Journal of Zhejiang University of Traditional Chinese Medicine. 2013;5:543–5. | no | NA | low | moderate | moderate | moderate | moderate |
| Zhu YC. An effect evaluation on the model of integrated drowning interventionsfor the floating children in Ningbo City. Prev Med. 2016;28 (11):1098–102. | yes | moderate | low | low | low | low | low |
| Zhu Y, Feng X, Li H, Huang Y, Chen J, Xu G. A randomized controlled trial to evaluate the impact of a geo-specific poster compared to a general poster for effecting change in perceived threat and intention to avoid drowning “hotspots” among children of migrant workers: evidence from Ningbo, China. BMC Public Health. 2017 May 30;17(1):530. | yes | low | low | low | low | low | moderate |
| Zimmerman K, Jinadasa D, Maegga B, Guerrero A. Road traffic injury on rural roads in Tanzania: measuring the effectiveness of a road safety program. Traffic Inj Prev. 2015 Jan 28;16(5):456–60. | no | low | low | moderate | low | low | low |

A2. CONSORT Tool for RCTs

| **Number of Study (Reference)** | **CONSORT** | | | | | | | | | | | | | | | | | | |
| --- | --- | --- | --- | --- | --- | --- | --- | --- | --- | --- | --- | --- | --- | --- | --- | --- | --- | --- | --- |
|  |  | | Background and Objectives | | Trial Design | | Participants | | Interventions | Outcomes | | Sample Size | | Randomization | | | | | |
|  |  |  |  |  |  |  |  |  |  |  |  |  |  | Sequence | | Allocation | Implementation | Blinding | |
|  | 1a | 1b | 2a | 2b | 3a | 3b | 4a | 4b | 5 | 6a | 6b | 7a | 7b | 8a | 8b | 9 | 10 | 11a | 11b |
| 1 (35) | Y | Y | Y | Y | Y | N | Y | Y | Y | Y | N/A | N | N | N | Y | N | N | N | Y |
| 2(36) | N | Y | Y | Y | Y | N | Y | Y | Y | Y | N/A | N | N | N | Y | N | N | N | Y |
| 3(42) | N | Y | Y | Y | Y | Y | Y | Y | Y | Y | N/A | N | N/A | N | N | N | N | N | N/A |
| 4 (43) | N | Y | Y | Y | Y | N | N | N | Y | Y | N/A | N | N/A | N/A | N/A | N/A | N/A | N/A | N/A |
| 5 (44) | N | Y | Y | Y | Y | N | Y | Y | Y | Y | N/A | N | N/A | N/A | N/A | N | N | N | N |
| 6 (45) | N | Y | Y | N | Y | N | N | Y | N | N | N | N | N | N | Y | N | N | N | N |
| 7 (62) | Y | Y | Y | Y | Y | N | Y | Y | Y | Y | N/A | Y | N | N | Y | N | N | N | Y |
| 8 (65) | N | Y | Y | Y | Y | N | N | N | N | Y | N/A | N | N/A | N/A | N/A | N/A | N/A | N/A | N/A |
| 9 (66) | Y | Y | Y | Y | Y | N | Y | Y | Y | Y | N/A | Y | N | Y | Y | Y | Y | N | Y |
| 10 (67) | N | Y | Y | N | N | N | Y | Y | Y | Y | N | N | N | N | Y | N | N | N | N |
| 11 (74) | Y | Y | Y | Y | Y | N | Y | Y | Y | Y | N/A | Y | N | Y | Y | Y | Y | N/A | Y |
| 12 (75) | Y | Y | Y | Y | Y | N | Y | Y | Y | Y | N/A | Y | N | Y | Y | Y | Y | N/A | N |
| 13 (79) | Y | Y | Y | Y | Y | N | Y | Y | Y | Y | N/A | Y | N/A | N/A | N/A | N/A | N/A | N/A | N |
| 14 (82) | N | Y | Y | Y | N/A |  | Y | Y | Y | Y | N/A | Y | N/A | Y | Y | Y | N | N/A | N/A |
| 15 (90) | Y | Y | Y | Y | Y | N | Y | Y | Y | Y | N/A | Y | N/A | Y | Y | Y | Y | Y | Y |
| 16 (97) | N | Y | Y | N | Y | N | N | Y | Y | Y | N | N | N | N | Y | N | Y | N | N |
| 17 (98) | N | Y | Y | Y | Y | N | Y | Y | Y | Y | N/A | N | N/A | N/A | N/A | N/A | N/A | N/A | N/A |
| 18(99) | N | Y | Y | Y | Y | N | Y | Y | N | N/A | N/A | N | N/A | N/A | N/A | N/A | N/A | N/A | N/A |
| 19(100) | N | Y | Y | Y | Y | N | Y | Y | Y | Y | N/A | N | N/A | N/A | N/A | N/A | N/A | N/A | N/A |

| **Number of Study (Reference)** | **CONSORT Continued** | | | | | | | | | | | | | | | | | | | | | | | | | | | | | |
| --- | --- | --- | --- | --- | --- | --- | --- | --- | --- | --- | --- | --- | --- | --- | --- | --- | --- | --- | --- | --- | --- | --- | --- | --- | --- | --- | --- | --- | --- | --- |
|  | Statistical Methods | | Participant Flow | | Recruitment | | | Baseline Data | | Numbers Analyzed | | Outcomes and Estimation | | | Ancillary Analysis | | Harms | | Limitations | | Generalizability | | Interpretation | | Registration | | Protocol | | Funding | |
|  | 12a | 12b | 13a | 13b | | 14a | 14b | | 15 | | 16 | | 17a | 17b | | 18 | | 19 | | 20 | | 21 | | 22 | | 23 | | 24 | | 25 |
| 1 (35) | Y | Y | Y | Y | | Y | Y | | Y | | Y | | N | N | | N | | N | | Y | | Y | | Y | | Y | | Y | | Y |
| 2(36) | Y | Y | Y | Y | | Y | Y | | Y | | Y | | N | N | | N | | N | | Y | | Y | | Y | | Y | | Y | | Y |
| 3(42) | Y | N/A | Y | N | | Y | N | | N | | Y | | N | N/A | | Y | | N | | Y | | N | | Y | | N | | N | | N |
| 4 (43) | Y | N/A | N | N | | Y | N | | Y | | Y | | N | Y | | N/A | | N | | Y | | Y | | Y | | N | | N | | N |
| 5 (44) | Y | N/A | N | N/A | | Y | N/A | | N | | Y | | N | N/A | | N/A | | N/A | | N/A | | Y | | Y | | N | | N | | N |
| 6 (45) | Y | N | N | N | | Y | N | | N | | Y | | Y | N | | N | | N | | Y | | N | | Y | | Y | | N | | Y |
| 7 (62) | Y | Y | Y | Y | | Y | Y | | Y | | Y | | Y | Y | | Y | | Y | | Y | | Y | | Y | | N | | N | | Y |
| 8 (65) | Y | N/A | N | N/A | | Y | N/A | | N | | Y | | N | N | | N/A | | N/A | | N/A | | Y | | Y | | N | | N | | N |
| 9 (66) | Y | Y | Y | N | | Y | Y | | Y | | Y | | Y | N | | Y | | N | | Y | | Y | | Y | | Y | | Y | | Y |
| 10 (67) | Y | N | Y | N | | Y | N | | Y | | Y | | Y | N | | N | | N | | Y | | Y | | Y | | Y | | N | | Y |
| 11 (74) | Y | Y | Y | Y | | Y | Y | | Y | | Y | | Y | N | | Y | | N | | Y | | Y | | Y | | N | | N | | N |
| 12 (75) | Y | N | Y | Y | | Y | Y | | Y | | Y | | Y | N | | N | | N | | Y | | Y | | Y | | N | | N | | Y |
| 13 (79) | Y | Y | Y | Y | | Y | Y | | Y | | Y | | Y | Y | | Y | | N | | Y | | Y | | Y | | N | | N | | Y |
| 14 (82) | Y | N/A | Y | N | | Y | Y | | Y | | Y | | Y | Y | | Y | | N | | Y | | Y | | Y | | N | | N | | Y |
| 15 (90) | Y | Y | Y | Y | | Y | N | | Y | | Y | | Y | N | | Y | | N/A | | Y | | Y | | Y | | Y | | Y | | N |
| 16 (97) | Y | N | Y | N | | Y | N | | N | | Y | | Y | N | | N | | N | | N | | Y | | Y | | N | | N | | Y |
| 17 (98) | Y | N/A | N | N | | Y | N | | N | | Y | | N | N | | N | | N | | N | | Y | | Y | | N | | N | | N |
| 18(99) | Y | N/A | N | N/A | | Y | N/A | | N | | Y | | N | N/A | | N/A | | N/A | | N/A | | Y | | Y | | N | | N | | N |
| 19(100) | Y | N/A | N | Y | | Y | N/A | | N | | Y | | N | N/A | | N/A | | N/A | | N/A | | Y | | Y | | N | | N | | N |
