# Supplementary material for: Prevention of childhood unintentional injuries in low- and middle-income countries: A systematic review
Source: PLoS One. 2020 Dec 29;15(12):e0243464. doi: 10.1371/journal.pone.0243464 (PMC7771986; doi:10.1371/journal.pone.0243464)
Supplement: S2 Appendix — (DOCX) [file pone.0243464.s002.docx]

S2 Appendix. Search Terms.

**B1: Initial Search Terms**

| #1 | "Accidents, Traffic"[Mesh] OR (("Motor Vehicles"[Mesh:NoExp] OR "Automobiles"[Mesh] OR "Motorcycles"[Mesh] OR traffic[tiab] OR vehicle[tiab] OR vehicular[tiab] OR car[tiab] OR cars[tiab] OR automobile[tiab] OR automobiles[tiab] OR motorcycle[tiab] OR motorcycles[tiab] OR taxi[tiab] OR cab[tiab] OR road[tiab] OR pedestrian[tiab] OR pedestrians[tiab]) AND (accident[tiab] OR accidents[tiab] OR injury[tiab] OR injuries[tiab] OR "Wounds and Injuries"[Mesh] OR "injuries" [Subheading])) | 67314 |
| --- | --- | --- |
| #2 | "Accidents, Traffic/prevention and control"[Mesh] OR "Social Control, Formal"[Mesh] OR "Protective Devices"[Mesh] OR "organization and administration" [Subheading] OR Law[tiab] OR laws[tiab] OR legal[tiab] OR regulation[tiab] OR regulations[tiab] OR "minimum age"[tiab] OR (minimum[tiab] AND (drinking[tiab] OR alcohol[tiab]) AND age[tiab]) OR "seat belt"[tiab] OR "seat belts"[tiab] OR restraint[tiab] OR restraints[tiab] OR helmet[tiab] OR helmets[tiab] OR speed[tiab] OR safety[tiab] OR light[tiab] OR lights[tiab] OT license[tiab] OR licenses[tiab] OR licensing[tiab] OR prevent*[tiab] | 1238215 |
| #3 | "Drowning"[Mesh] OR drowning[tiab] OR drowned[tiab] OR drowns[tiab] | 5575 |
| #4 | "Drowning/prevention and control"[Mesh] OR "Protective Devices"[Mesh] OR "Resuscitation"[Mesh] OR Hazard[tiab] OR hazards[tiab] OR fencing[tiab] OR fence[tiab] OR fenced[tiab] OR fences[tiab] OR flotation[tiab] OR safety[tiab] OR resuscitation[tiab] OR resuscitate[tiab] OR resuscitated[tiab] | 713045 |
| #5 | "Burns"[Mesh] OR "Smoke Inhalation Injury"[Mesh] OR burn[tiab] OR burns[tiab] | 70666 |
| #6 | "Burns/prevention and control"[Mesh] OR "Fires/prevention and control"[Mesh] OR "Protective Devices"[Mesh] OR "Hot Temperature"[Mesh] OR "Health Education"[Mesh] OR "Burn Units"[Mesh] OR "smoke alarm"[tiab] OR "smoke alarms"[tiab] OR "child resistant"[tiab] OR "hot water"[tiab] OR education[tiab] OR "burn center"[tiab] OR "burn centers"[tiab] OR "burn unit"[tiab] OR "burn units"[tiab] | 716513 |
| #7 | "Accidental Falls"[Mesh] OR falls[tiab] | 48265 |
| #8 | "Accidental Falls/prevention and control"[Mesh] OR "Interior Design and Furnishings"[Mesh] OR "Infant Equipment"[Mesh] OR "Protective Devices"[Mesh] OR Furniture[tiab] OR program[tiab] OR programs[tiab] OR intervention[tiab] OR interventions[tiab] OR playground[tiab] OR play[tiab] OR window[tiab] OR windows[tiab] | 1885700 |
| #9 | "Poisoning"[Mesh] OR "poisoning" [Subheading] OR ingestion[tiab] | 246982 |
| #10 | "Poisoning/prevention and control"[Mesh] OR "Drug Packaging"[Mesh] OR "Poison Control Centers"[Mesh] OR "child resistant"[tiab] OR packaging[tiab] OR package[tiab] OR "poison control"[tiab] | 74889 |
| #11 | (#1 AND #2) OR (#3 AND #4) OR (#5 AND #6) OR (#7 AND #8) OR (#9 AND #10) | 51033 |
| #12 | "Developing Countries"[Mesh] OR "Afghanistan"[Mesh] OR "Bangladesh"[Mesh] OR "Benin"[Mesh] OR "Burkina Faso"[Mesh] OR "Burundi"[Mesh] OR "Cambodia"[Mesh] OR "Central African Republic"[Mesh] OR "Chad"[Mesh] OR "Comoros"[Mesh] OR "Democratic Republic of the Congo"[Mesh] OR "Eritrea"[Mesh] OR "Ethiopia"[Mesh] OR "Gambia"[Mesh] OR "Guinea"[Mesh] OR "Guinea-Bissau"[Mesh] OR "Haiti"[Mesh] OR "Kenya"[Mesh] OR "Democratic People's Republic of Korea"[Mesh] OR "Liberia"[Mesh] OR "Madagascar"[Mesh] OR "Malawi"[Mesh] OR "Mali"[Mesh] OR "Mozambique"[Mesh] OR "Myanmar"[Mesh] OR "Nepal"[Mesh] OR "Niger"[Mesh] OR "Rwanda"[Mesh] OR "Sierra Leone"[Mesh] OR "Somalia"[Mesh] OR "Tajikistan"[Mesh] OR "Tanzania"[Mesh] OR "Togo"[Mesh] OR "Uganda"[Mesh] OR "Zimbabwe"[Mesh] OR "Armenia"[Mesh] OR "Bhutan"[Mesh] OR "Bolivia"[Mesh] OR "Cameroon"[Mesh] OR "Cape Verde"[Mesh] OR "Congo"[Mesh] OR "Cote d'Ivoire"[Mesh] OR "Djibouti"[Mesh] OR "Egypt"[Mesh] OR "El Salvador"[Mesh] OR "Georgia (Republic)"[Mesh] OR "Ghana"[Mesh] OR "Guatemala"[Mesh] OR "Guyana"[Mesh] OR "Honduras"[Mesh] OR "Indonesia"[Mesh] OR "India"[Mesh] OR "Kosovo"[Mesh] OR "Kyrgyzstan"[Mesh] OR "Laos"[Mesh] OR "Lesotho"[Mesh] OR "Mauritania"[Mesh] OR "Micronesia"[Mesh] OR "Moldova"[Mesh] OR "Mongolia"[Mesh] OR "Morocco"[Mesh] OR "Nicaragua"[Mesh] OR "Nigeria"[Mesh] OR "Pakistan"[Mesh] OR "Papua New Guinea"[Mesh] OR "Paraguay"[Mesh] OR "Philippines"[Mesh] OR "Independent State of Samoa"[Mesh] OR "Atlantic Islands"[Mesh] OR "Senegal"[Mesh] OR "Melanesia"[Mesh] OR "Sri Lanka"[Mesh] OR "Sudan"[Mesh] OR "Swaziland"[Mesh] OR "Syria"[Mesh] OR "Timor-Leste"[Mesh] OR "Ukraine"[Mesh] OR "Uzbekistan"[Mesh] OR "Vanuatu"[Mesh] OR "Vietnam"[Mesh] OR "Middle East"[Mesh] OR "Yemen"[Mesh] OR "Zambia"[Mesh] OR "Angola"[Mesh] OR "Albania"[Mesh] OR "Algeria"[Mesh] OR "American Samoa"[Mesh] OR "Argentina"[Mesh] OR "Azerbaijan"[Mesh] OR "Republic of Belarus"[Mesh] OR "Belize"[Mesh] OR "Bosnia and Herzegovina"[Mesh] OR "Botswana"[Mesh] OR "Brazil"[Mesh] OR "Bulgaria"[Mesh] OR "China"[Mesh] OR "Colombia"[Mesh] OR "Costa Rica"[Mesh] OR "Cuba"[Mesh] OR "Dominica"[Mesh] OR "Dominican Republic"[Mesh] OR "Ecuador"[Mesh] OR "Equatorial Guinea"[Mesh] OR "Fiji"[Mesh] OR "Gabon"[Mesh] OR "Grenada"[Mesh] OR "Iran"[Mesh] OR "Iraq"[Mesh] OR "Jamaica"[Mesh] OR "Jordan"[Mesh] OR "Kazakhstan"[Mesh] OR "Lebanon"[Mesh] OR "Libya"[Mesh] OR "Macedonia (Republic)"[Mesh] OR "Malaysia"[Mesh] OR "Indian Ocean Islands"[Mesh] OR "Mexico"[Mesh] OR "Montenegro"[Mesh] OR "Namibia"[Mesh] OR "Palau"[Mesh] OR "Panama"[Mesh] OR "Peru"[Mesh] OR "Romania"[Mesh] OR "Russia"[Mesh] OR "Serbia"[Mesh] OR "Seychelles"[Mesh] OR "South Africa"[Mesh] OR "Saint Lucia"[Mesh] OR "Saint Vincent and the Grenadines"[Mesh] OR "Suriname"[Mesh] OR "Thailand"[Mesh] OR "Tonga"[Mesh] OR "Tunisia"[Mesh] OR "Turkey"[Mesh] OR "Turkmenistan"[Mesh] OR "Venezuela"[Mesh] OR "Afghanistan"[all fields] OR "Bangladesh"[all fields] OR "Benin"[all fields] OR "Burkina Faso"[all fields] OR "Burundi"[all fields] OR "Cambodia"[all fields] OR "cabo verde"[all fields] OR "Central African Republic"[all fields] OR "Chad"[all fields] OR "Comoros"[all fields] OR "Democratic Republic of the Congo"[all fields] OR "Eritrea"[all fields] OR "Ethiopia"[all fields] OR "Gambia"[all fields] OR "Guinea"[all fields] OR "Guinea-Bissau"[all fields] OR "Haiti"[all fields] OR "Kenya"[all fields] OR "Democratic People's Republic of Korea"[all fields] OR "Liberia"[all fields] OR "Madagascar"[all fields] OR "Malawi"[all fields] OR "Mali"[all fields] OR "Mozambique"[all fields] OR "Myanmar"[all fields] OR "Nepal"[all fields] OR "Niger"[all fields] OR "Rwanda"[all fields] OR "Sierra Leone"[all fields] OR "Somalia"[all fields] OR "Tajikistan"[all fields] OR "Tanzania"[all fields] OR "Togo"[all fields] OR "Uganda"[all fields] OR "Zimbabwe"[all fields] OR "Armenia"[all fields] OR "Bhutan"[all fields] OR "Bolivia"[all fields] OR "Cameroon"[all fields] OR "Cape Verde"[all fields] OR "Congo"[all fields] OR "Cote d'Ivoire"[all fields] OR "Djibouti"[all fields] OR "Egypt"[all fields] OR "El Salvador"[all fields] OR "Georgia (Republic)"[all fields] OR "Ghana"[all fields] OR "Guatemala"[all fields] OR "Guyana"[all fields] OR "Honduras"[all fields] OR "Indonesia"[all fields] OR "India"[all fields] OR “Kiribati”[all fields] OR "Kosovo"[all fields] OR "Kyrgyzstan"[all fields] OR "Kyrgyz"[all fields] OR "Laos"[all fields] OR "lao"[all fields] OR "Lesotho"[all fields] OR "Mauritania"[all fields] OR "Micronesia"[all fields] OR "Moldova"[all fields] OR "Mongolia"[all fields] OR "Morocco"[all fields] OR "Nicaragua"[all fields] OR "Nigeria"[all fields] OR "Pakistan"[all fields] OR "Papua New Guinea"[all fields] OR "Paraguay"[all fields] OR "Philippines"[all fields] OR "Independent State of Samoa"[all fields] OR "Atlantic Islands"[all fields] OR "Sao Tome"[all fields] OR Principe[all fields] OR "Senegal"[all fields] OR "Melanesia"[all fields] OR "Solomon islands"[all fields] OR "Sri Lanka"[all fields] OR "Sudan"[all fields] OR "Swaziland"[all fields] OR "Syria"[all fields] OR "East Timor"[all fields] OR "Timor leste"[all fields] OR "Ukraine"[all fields] OR "Uzbekistan"[all fields] OR "Vanuatu"[all fields] OR "Vietnam"[all fields] OR "Middle East"[all fields] OR "west bank"[all fields] OR "Gaza"[all fields] OR "Yemen"[all fields] OR "Zambia"[all fields] OR "Angola"[all fields] OR "Albania"[all fields] OR "Algeria"[all fields] OR "Argentina"[all fields] OR "Samoa"[all fields] OR "Azerbaijan"[all fields] OR "Republic of Belarus"[all fields] OR "Belize"[all fields] OR "Bosnia-Herzegovina"[all fields] OR "Botswana"[all fields] OR "Brazil"[all fields] OR "Bulgaria"[all fields] OR "China"[all fields] OR "Colombia"[all fields] OR "Costa Rica"[all fields] OR "Cuba"[all fields] OR "Dominica"[all fields] OR "Dominican Republic"[all fields] OR "Ecuador"[all fields] OR "Equatorial Guinea"[all fields] OR "Fiji"[all fields] OR "Gabon"[all fields] OR "Grenada"[all fields] OR "Iran"[all fields] OR "Iraq"[all fields] OR "Jamaica"[all fields] OR "Jordan"[all fields] OR "Kazakhstan"[all fields] OR "Lebanon"[all fields] OR "Libya"[all fields] OR "Macedonia"[all fields] OR "Malaysia"[all fields] OR "Indian Ocean Islands"[all fields] OR "Maldives"[all fields] OR “Marshall Islands”[all fields] OR "Mauritius"[all fields] OR "Mexico"[all fields] OR "Montenegro"[all fields] OR "Namibia"[all fields] OR "Palau"[all fields] OR "Panama"[all fields] OR "Peru"[all fields] OR "Romania"[all fields] OR "Russia"[all fields] OR "Russian Federation"[all fields] OR "Serbia"[all fields] OR "Seychelles"[all fields] OR "South Africa"[all fields] OR "Saint Lucia"[all fields] OR "Saint Vincent and the Grenadines"[all fields] OR "Suriname"[all fields] OR "Thailand"[all fields] OR "Tonga"[all fields] OR "Tunisia"[all fields] OR "Turkey"[all fields] OR "Turkmenistan"[all fields] OR "Tuvalu"[all fields] OR "Venezuela"[all fields] OR "low resource"[all fields] OR "under-resourced"[all fields] OR "resource poor"[all fields] OR "under-developed"[all fields] OR "underdeveloped"[all fields] OR "developing country"[all fields] OR "developing countries"[all fields] OR "developing world"[all fields] OR "third world" [all fields] OR lmic[all fields] OR (low[all fields] AND middle[all fields] AND income[all fields]) | 3717340 |
| #13 | #12 AND #13 | 8877 |
| #14 | "Pediatrics"[Mesh] OR "Adolescent"[Mesh] OR "Infant"[Mesh] OR "Child"[Mesh] OR child[tiab] OR children[tiab] OR infant[tiab] OR infants[tiab] OR preschool[tiab] OR preschooler[tiab] OR pediatric[tiab] OR teenager[tiab] OR teenagers[tiab] OR teenaged[tiab] OR teen[tiab] OR teens[tiab] OR adolescent[tiab] OR adolescents[tiab] OR adolescence[tiab] OR youth[tiab] | 3612211 |
| #15 | #13 AND #14 | 2797 |
| #16 | (randomized controlled trial[pt] OR controlled clinical trial[pt] OR randomized[tiab] OR randomised[tiab] OR randomization[tiab] OR randomisation[tiab] OR placebo[tiab] OR drug therapy[sh] OR randomly[tiab] OR trial[tiab] OR groups[tiab] OR Clinical trial[pt] OR “clinical trial”[tiab] OR “clinical trials”[tiab] OR "evaluation studies"[Publication Type] OR "evaluation studies as topic"[MeSH Terms] OR "evaluation study"[tiab] OR evaluation studies[tiab] OR "intervention study"[tiab] OR "intervention studies"[tiab] OR "case-control studies"[MeSH Terms] OR "case-control"[tiab] OR "cohort studies"[MeSH Terms] OR cohort[tiab] OR "longitudinal studies"[MeSH Terms] OR "longitudinal”[tiab] OR longitudinally[tiab] OR "prospective"[tiab] OR prospectively[tiab] OR "retrospective studies"[MeSH Terms] OR "retrospective"[tiab] OR "follow up"[tiab] OR "comparative study"[Publication Type] OR "comparative study"[tiab] OR systematic[subset] OR "meta-analysis"[Publication Type] OR "meta-analysis as topic"[MeSH Terms] OR "meta-analysis"[tiab] OR "meta-analyses"[tiab]) NOT (Editorial[ptyp] OR Letter[ptyp] OR Case Reports[ptyp] OR Comment[ptyp]) NOT (animals[mh] NOT humans[mh]) | 6341231 |
| #17 | #15 AND #16 | 1643 |

**B2. Second Search Terms.**

| #1 | "Accidents, Traffic"[Mesh] OR (("Motor Vehicles"[Mesh:NoExp] OR "Automobiles"[Mesh] OR "Motorcycles"[Mesh] OR traffic[tiab] OR vehicle[tiab] OR vehicular[tiab] OR car[tiab] OR cars[tiab] OR automobile[tiab] OR automobiles[tiab] OR motorcycle[tiab] OR motorcycles[tiab] OR taxi[tiab] OR cab[tiab] OR road[tiab] OR pedestrian[tiab] OR pedestrians[tiab]) AND (accident[tiab] OR accidents[tiab] OR injury[tiab] OR injuries[tiab] OR "Wounds and Injuries"[Mesh] OR "injuries" [Subheading])) | 71279 |
| --- | --- | --- |
| #2 | "Accidents, Traffic/prevention and control"[Mesh] OR "Social Control, Formal"[Mesh] OR "Protective Devices"[Mesh] OR "organization and administration" [Subheading] OR "Safety"[Mesh] OR Law[tiab] OR laws[tiab] OR legal[tiab] OR regulation[tiab] OR regulations[tiab] OR "minimum age"[tiab] OR (minimum[tiab] AND (drinking[tiab] OR alcohol[tiab]) AND age[tiab]) OR "seat belt"[tiab] OR "seat belts"[tiab] OR restraint[tiab] OR restraints[tiab] OR helmet[tiab] OR helmets[tiab] OR speed[tiab] OR safety[tiab] OR light[tiab] OR lights[tiab] OR license[tiab] OR licenses[tiab] OR licensing[tiab] OR prevent*[tiab] | 5152889 |
| #3 | "Drowning"[Mesh] OR drowning[tiab] OR drowned[tiab] OR drowns[tiab] | 5872 |
| #4 | "Drowning/prevention and control"[Mesh] OR "Protective Devices"[Mesh] OR "Resuscitation"[Mesh] OR Hazard[tiab] OR hazards[tiab] OR fencing[tiab] OR fence[tiab] OR fenced[tiab] OR fences[tiab] OR flotation[tiab] OR safety[tiab] OR resuscitation[tiab] OR resuscitate[tiab] OR resuscitated[tiab] | 791792 |
| #5 | "Burns"[Mesh] OR "Smoke Inhalation Injury"[Mesh] OR burn[tiab] OR burns[tiab] OR (smoke[tiab] AND inhal*[tiab]) | 77462 |
| #6 | "Burns/prevention and control"[Mesh] OR "Fires/prevention and control"[Mesh] OR "Protective Devices"[Mesh] OR "Hot Temperature"[Mesh] OR "Health Education"[Mesh] OR "Burn Units"[Mesh] OR "smoke alarm"[tiab] OR "smoke alarms"[tiab] OR "child resistant"[tiab] OR "hot water"[tiab] OR education[tiab] OR "burn center"[tiab] OR "burn centers"[tiab] OR "burn unit"[tiab] OR "burn units"[tiab] | 765817 |
| #7 | "Accidental Falls"[Mesh] OR falls[ti] OR fall[ti] | 29519 |
| #8 | "Accidental Falls/prevention and control"[Mesh] OR "Interior Design and Furnishings"[Mesh] OR "Infant Equipment"[Mesh] OR "Protective Devices"[Mesh] OR Furniture[tiab] OR program[tiab] OR programs[tiab] OR intervention[tiab] OR interventions[tiab] OR playground[tiab] OR play[tiab] OR window[tiab] OR windows[tiab] | 2061290 |
| #9 | "Poisoning"[Mesh] OR "poisoning" [Subheading] OR ingestion[tiab] OR poisoning[tiab] OR poisoned[tiab] | 276628 |
| #10 | "Poisoning/prevention and control"[Mesh] OR "Drug Packaging"[Mesh] OR "Poison Control Centers"[Mesh] OR "child resistant"[tiab] OR packaging[tiab] OR package[tiab] OR "poison control"[tiab] | 82075 |
| #11 | (#1 AND #2) OR (#3 AND #4) OR (#5 AND #6) OR (#7 AND #8) OR (#9 AND #10) | 70857 |
| #12 | "Developing Countries"[Mesh] OR "Afghanistan"[Mesh] OR "Bangladesh"[Mesh] OR "Benin"[Mesh] OR "Burkina Faso"[Mesh] OR "Burundi"[Mesh] OR "Cambodia"[Mesh] OR "Central African Republic"[Mesh] OR "Chad"[Mesh] OR "Comoros"[Mesh] OR "Democratic Republic of the Congo"[Mesh] OR "Eritrea"[Mesh] OR "Ethiopia"[Mesh] OR "Gambia"[Mesh] OR "Guinea"[Mesh] OR "Guinea-Bissau"[Mesh] OR "Haiti"[Mesh] OR "Kenya"[Mesh] OR "Democratic People's Republic of Korea"[Mesh] OR "Liberia"[Mesh] OR "Madagascar"[Mesh] OR "Malawi"[Mesh] OR "Mali"[Mesh] OR "Mozambique"[Mesh] OR "Myanmar"[Mesh] OR "Nepal"[Mesh] OR "Niger"[Mesh] OR "Rwanda"[Mesh] OR "Sierra Leone"[Mesh] OR "Somalia"[Mesh] OR "Tajikistan"[Mesh] OR "Tanzania"[Mesh] OR "Togo"[Mesh] OR "Uganda"[Mesh] OR "Zimbabwe"[Mesh] OR "Armenia"[Mesh] OR "Bhutan"[Mesh] OR "Bolivia"[Mesh] OR "Cameroon"[Mesh] OR "Cabo Verde"[Mesh] OR "Congo"[Mesh] OR "Cote d'Ivoire"[Mesh] OR "Djibouti"[Mesh] OR "Egypt"[Mesh] OR "El Salvador"[Mesh] OR "Georgia (Republic)"[Mesh] OR "Ghana"[Mesh] OR "Guatemala"[Mesh] OR "Guyana"[Mesh] OR "Honduras"[Mesh] OR "Indonesia"[Mesh] OR "India"[Mesh] OR "Kosovo"[Mesh] OR "Kyrgyzstan"[Mesh] OR "Laos"[Mesh] OR "Lesotho"[Mesh] OR "Mauritania"[Mesh] OR "Micronesia"[Mesh] OR "Moldova"[Mesh] OR "Mongolia"[Mesh] OR "Morocco"[Mesh] OR "Nicaragua"[Mesh] OR "Nigeria"[Mesh] OR "Pakistan"[Mesh] OR "Papua New Guinea"[Mesh] OR "Paraguay"[Mesh] OR "Philippines"[Mesh] OR "Independent State of Samoa"[Mesh] OR "Atlantic Islands"[Mesh] OR "Senegal"[Mesh] OR "Melanesia"[Mesh] OR "Sri Lanka"[Mesh] OR "Sudan"[Mesh] OR "Swaziland"[Mesh] OR "Syria"[Mesh] OR "Timor-Leste"[Mesh] OR "Ukraine"[Mesh] OR "Uzbekistan"[Mesh] OR "Vanuatu"[Mesh] OR "Vietnam"[Mesh] OR "Middle East"[Mesh] OR "Yemen"[Mesh] OR "Zambia"[Mesh] OR "Angola"[Mesh] OR "Albania"[Mesh] OR "Algeria"[Mesh] OR "American Samoa"[Mesh] OR "Argentina"[Mesh] OR "Azerbaijan"[Mesh] OR "Republic of Belarus"[Mesh] OR "Belize"[Mesh] OR "Bosnia and Herzegovina"[Mesh] OR "Botswana"[Mesh] OR "Brazil"[Mesh] OR "Bulgaria"[Mesh] OR "China"[Mesh] OR "Colombia"[Mesh] OR "Costa Rica"[Mesh] OR "Cuba"[Mesh] OR "Dominica"[Mesh] OR "Dominican Republic"[Mesh] OR "Ecuador"[Mesh] OR "Equatorial Guinea"[Mesh] OR "Fiji"[Mesh] OR "Gabon"[Mesh] OR "Grenada"[Mesh] OR "Iran"[Mesh] OR "Iraq"[Mesh] OR "Jamaica"[Mesh] OR "Jordan"[Mesh] OR "Kazakhstan"[Mesh] OR "Lebanon"[Mesh] OR "Libya"[Mesh] OR "Macedonia (Republic)"[Mesh] OR "Malaysia"[Mesh] OR "Indian Ocean Islands"[Mesh] OR "Mexico"[Mesh] OR "Montenegro"[Mesh] OR "Namibia"[Mesh] OR "Palau"[Mesh] OR "Panama"[Mesh] OR "Peru"[Mesh] OR "Romania"[Mesh] OR "Russia"[Mesh] OR "Serbia"[Mesh] OR "Seychelles"[Mesh] OR "South Africa"[Mesh] OR "Saint Lucia"[Mesh] OR "Saint Vincent and the Grenadines"[Mesh] OR "Suriname"[Mesh] OR "Thailand"[Mesh] OR "Tonga"[Mesh] OR "Tunisia"[Mesh] OR "Turkey"[Mesh] OR "Turkmenistan"[Mesh] OR "Venezuela"[Mesh] OR "Afghanistan"[all fields] OR "Bangladesh"[all fields] OR "Benin"[all fields] OR "Burkina Faso"[all fields] OR "Burundi"[all fields] OR "Cambodia"[all fields] OR "cabo verde"[all fields] OR "Central African Republic"[all fields] OR "Chad"[all fields] OR "Comoros"[all fields] OR "Democratic Republic of the Congo"[all fields] OR "Eritrea"[all fields] OR "Ethiopia"[all fields] OR "Gambia"[all fields] OR "Guinea"[all fields] OR "Guinea-Bissau"[all fields] OR "Haiti"[all fields] OR "Kenya"[all fields] OR "Democratic People's Republic of Korea"[all fields] OR "Liberia"[all fields] OR "Madagascar"[all fields] OR "Malawi"[all fields] OR "Mali"[all fields] OR "Mozambique"[all fields] OR "Myanmar"[all fields] OR "Nepal"[all fields] OR "Niger"[all fields] OR "Rwanda"[all fields] OR "Sierra Leone"[all fields] OR "Somalia"[all fields] OR "Tajikistan"[all fields] OR "Tanzania"[all fields] OR "Togo"[all fields] OR "Uganda"[all fields] OR "Zimbabwe"[all fields] OR "Armenia"[all fields] OR "Bhutan"[all fields] OR "Bolivia"[all fields] OR "Cameroon"[all fields] OR "Cape Verde"[all fields] OR "Congo"[all fields] OR "Cote d'Ivoire"[all fields] OR "Djibouti"[all fields] OR "Egypt"[all fields] OR "El Salvador"[all fields] OR "Georgia (Republic)"[all fields] OR "Ghana"[all fields] OR "Guatemala"[all fields] OR "Guyana"[all fields] OR "Honduras"[all fields] OR "Indonesia"[all fields] OR "India"[all fields] OR “Kiribati”[all fields] OR "Kosovo"[all fields] OR "Kyrgyzstan"[all fields] OR "Kyrgyz"[all fields] OR "Laos"[all fields] OR "lao"[all fields] OR "Lesotho"[all fields] OR "Mauritania"[all fields] OR "Micronesia"[all fields] OR "Moldova"[all fields] OR "Mongolia"[all fields] OR "Morocco"[all fields] OR "Nicaragua"[all fields] OR "Nigeria"[all fields] OR "Pakistan"[all fields] OR "Papua New Guinea"[all fields] OR "Paraguay"[all fields] OR "Philippines"[all fields] OR "Independent State of Samoa"[all fields] OR "Atlantic Islands"[all fields] OR "Sao Tome"[all fields] OR Principe[all fields] OR "Senegal"[all fields] OR "Melanesia"[all fields] OR "Solomon islands"[all fields] OR "Sri Lanka"[all fields] OR "Sudan"[all fields] OR "Swaziland"[all fields] OR "Syria"[all fields] OR "East Timor"[all fields] OR "Timor leste"[all fields] OR "Ukraine"[all fields] OR "Uzbekistan"[all fields] OR "Vanuatu"[all fields] OR "Vietnam"[all fields] OR "Middle East"[all fields] OR "west bank"[all fields] OR "Gaza"[all fields] OR "Yemen"[all fields] OR "Zambia"[all fields] OR "Angola"[all fields] OR "Albania"[all fields] OR "Algeria"[all fields] OR "Argentina"[all fields] OR "Samoa"[all fields] OR "Azerbaijan"[all fields] OR "Republic of Belarus"[all fields] OR "Belize"[all fields] OR "Bosnia-Herzegovina"[all fields] OR "Botswana"[all fields] OR "Brazil"[all fields] OR "Bulgaria"[all fields] OR "China"[all fields] OR "Colombia"[all fields] OR "Costa Rica"[all fields] OR "Cuba"[all fields] OR "Dominica"[all fields] OR "Dominican Republic"[all fields] OR "Ecuador"[all fields] OR "Equatorial Guinea"[all fields] OR "Fiji"[all fields] OR "Gabon"[all fields] OR "Grenada"[all fields] OR "Iran"[all fields] OR "Iraq"[all fields] OR "Jamaica"[all fields] OR "Jordan"[all fields] OR "Kazakhstan"[all fields] OR "Lebanon"[all fields] OR "Libya"[all fields] OR "Macedonia"[all fields] OR "Malaysia"[all fields] OR "Indian Ocean Islands"[all fields] OR "Maldives"[all fields] OR “Marshall Islands”[all fields] OR "Mauritius"[all fields] OR "Mexico"[all fields] OR "Montenegro"[all fields] OR "Namibia"[all fields] OR "Palau"[all fields] OR "Panama"[all fields] OR "Peru"[all fields] OR "Romania"[all fields] OR "Russia"[all fields] OR "Russian Federation"[all fields] OR "Serbia"[all fields] OR "Seychelles"[all fields] OR "South Africa"[all fields] OR "Saint Lucia"[all fields] OR "Saint Vincent and the Grenadines"[all fields] OR "Suriname"[all fields] OR "Thailand"[all fields] OR "Tonga"[all fields] OR "Tunisia"[all fields] OR "Turkey"[all fields] OR "Turkmenistan"[all fields] OR "Tuvalu"[all fields] OR "Venezuela"[all fields] OR "low resource"[all fields] OR "under-resourced"[all fields] OR "resource poor"[all fields] OR "under-developed"[all fields] OR "underdeveloped"[all fields] OR "developing country"[all fields] OR "developing countries"[all fields] OR "developing world"[all fields] OR "third world" [all fields] OR lmic[all fields] OR (low[all fields] AND middle[all fields] AND income[all fields]) | 4221012 |
| #13 | #11 AND #12 | 12566 |
| #14 | "Pediatrics"[Mesh] OR "Adolescent"[Mesh] OR "Infant"[Mesh] OR "Child"[Mesh] OR Infancy[tiab] OR newborn[tiab] OR newborns[tiab] OR neonatal[tiab] OR neonate[tiab] OR baby[tiab] OR babies[tiab] OR toddler[tiab] OR toddlers[tiab] OR child[tiab] OR children[tiab] OR infant[tiab] OR infants[tiab] OR preschool[tiab] OR preschooler[tiab] OR pediatric[tiab] OR teenager[tiab] OR teenagers[tiab] OR teenaged[tiab] OR teen[tiab] OR teens[tiab] OR adolescent[tiab] OR adolescents[tiab] OR adolescence[tiab] OR youth[tiab] OR boy[tiab] OR boys[tiab] OR boyhood[tiab] OR girl[tiab] OR girls[tiab] OR girlhood[tiab] OR kid[tiab] OR kids[tiab] OR schoolchild[tiab] OR "school child"[tiab] OR "school age"[tiab] OR paediatric[tiab] OR paediatrics[tiab] | 3964181 |
| #15 | #13 AND #14 | 3876 |
| #16 | (randomized controlled trial[pt] OR controlled clinical trial[pt] OR randomized[tiab] OR randomised[tiab] OR randomization[tiab] OR randomisation[tiab] OR placebo[tiab] OR drug therapy[sh] OR randomly[tiab] OR trial[tiab] OR groups[tiab] OR Clinical trial[pt] OR “clinical trial”[tiab] OR “clinical trials”[tiab] OR "evaluation studies"[Publication Type] OR "evaluation studies as topic"[MeSH Terms] OR "evaluation study"[tiab] OR evaluation studies[tiab] OR "intervention study"[tiab] OR "intervention studies"[tiab] OR "case-control studies"[MeSH Terms] OR "case-control"[tiab] OR "cohort studies"[MeSH Terms] OR cohort[tiab] OR "longitudinal studies"[MeSH Terms] OR "longitudinal”[tiab] OR longitudinally[tiab] OR "prospective"[tiab] OR prospectively[tiab] OR "retrospective studies"[MeSH Terms] OR "retrospective"[tiab] OR "follow up"[tiab] OR "comparative study"[Publication Type] OR "comparative study"[tiab] OR systematic[subset] OR "meta-analysis"[Publication Type] OR "meta-analysis as topic"[MeSH Terms] OR "meta-analysis"[tiab] OR "meta-analyses"[tiab]) NOT (Editorial[ptyp] OR Letter[ptyp] OR Case Reports[ptyp] OR Comment[ptyp]) NOT (animals[mh] NOT humans[mh]) | 6704588 |
| #17 | #15 AND #16 | 2205 |
| #18 | "2018/01/01"[Date - Entrez] : "3000"[Date - Entrez] AND #17 | 111 |

Embase:

|  |  |  |
| --- | --- | --- |
| #1 | 'traffic accident'/exp OR (('motor vehicle'/exp OR traffic:ab,ti OR vehicle:ab,ti OR vehicular:ab,ti OR car:ab,ti OR cars:ab,ti OR automobile:ab,ti OR automobiles:ab,ti OR motorcycle:ab,ti OR motorcycles:ab,ti OR taxi:ab,ti OR cab:ab,ti OR road:ab,ti OR pedestrian:ab,ti OR pedestrians:ab,ti) AND (accident:ab,ti OR accidents:ab,ti OR injury:ab,ti OR injuries:ab,ti OR 'injury'/exp)) | 103055 |
| #2 | 'legal aspect'/exp OR 'helmet'/exp OR 'traffic safety'/exp OR 'driver licence'/exp OR Law:ab,ti OR laws:ab,ti OR legal:ab,ti OR regulation:ab,ti OR regulations:ab,ti OR "minimum age":ab,ti OR (minimum:ab,ti AND (drinking:ab,ti OR alcohol:ab,ti) AND age:ab,ti) OR "seat belt":ab,ti OR "seat belts":ab,ti OR restraint:ab,ti OR restraints:ab,ti OR helmet:ab,ti OR helmets:ab,ti OR speed:ab,ti OR safety:ab,ti OR light:ab,ti OR lights:ab,ti OR license:ab,ti OR licenses:ab,ti OR licensing:ab,ti OR prevent*:ab,ti | 5115543 |
| #3 | 'drowning'/exp OR drowning:ab,ti OR drowned:ab,ti OR drowns:ab,ti | 8268 |
| #4 | 'drowning'/exp/dm_pc OR 'protective equipment'/exp OR 'resuscitation'/exp OR Hazard:ab,ti OR hazards:ab,ti OR fencing:ab,ti OR fence:ab,ti OR fenced:ab,ti OR fences:ab,ti OR flotation:ab,ti OR safety:ab,ti OR resuscitation:ab,ti OR resuscitate:ab,ti OR resuscitated:ab,ti | 1143962 |
| #5 | 'burn'/exp OR burn:ab,ti OR burns:ab,ti OR (smoke:ab,ti AND inhal*:ab,ti) | 106715 |
| #6 | 'burn'/exp/dm_pc OR 'fire protection'/exp OR 'protective equipment'/exp OR 'health promotion'/exp OR 'burn unit'/exp OR "smoke alarm":ab,ti OR "smoke alarms":ab,ti OR "child resistant":ab,ti OR "hot water":ab,ti OR education:ab,ti OR "burn center":ab,ti OR "burn centers":ab,ti OR "burn unit":ab,ti OR "burn units":ab,ti | 693895 |
| #7 | 'falling'/exp OR falls:ti OR fall:ti | 47873 |
| #8 | 'furniture'/exp OR 'infant equipment'/exp OR 'protective equipment'/exp OR Furniture:ab,ti OR program:ab,ti OR programs:ab,ti OR intervention:ab,ti OR interventions:ab,ti OR playground:ab,ti OR play:ab,ti OR window:ab,ti OR windows:ab,ti | 2743993 |
| #9 | 'intoxication'/exp OR ingestion:ab,ti OR poisoning:ab,ti OR poisoned:ab,ti | 517954 |
| #10 | 'intoxication'/exp/dm_pc OR 'drug packaging'/exp OR 'poison center'/exp OR "child resistant":ab,ti OR packaging:ab,ti OR package:ab,ti OR "poison control":ab,ti | 97059 |
| #11 | (#1 AND #2) OR (#3 AND #4) OR (#5 AND #6) OR (#7 AND #8) OR (#9 AND #10) | 75196 |
| #12 | 'developing country'/exp OR 'Afghanistan'/exp OR 'Bangladesh'/exp OR 'Benin'/exp OR 'Burkina Faso'/exp OR 'Burundi'/exp OR 'Cambodia'/exp OR 'Central African Republic'/exp OR 'Chad'/exp OR 'Comoros'/exp OR 'Democratic Republic Congo'/exp OR 'Congo'/exp OR 'Eritrea'/exp OR 'Ethiopia'/exp OR 'Gambia'/exp OR 'Guinea'/exp OR 'Guinea-Bissau'/exp OR 'Haiti'/exp OR 'Kenya'/exp OR 'North Korea'/exp OR 'Liberia'/exp OR 'Madagascar'/exp OR 'Malawi'/exp OR 'Mozambique'/exp OR 'Myanmar'/exp OR 'Nepal'/exp OR 'Niger'/exp OR 'Nigeria'/exp OR 'Rwanda'/exp OR 'Sierra Leone'/exp OR 'Somalia'/exp OR 'Tajikistan'/exp OR 'Tanzania'/exp OR 'Togo'/exp OR 'Uganda'/exp OR 'Zimbabwe'/exp OR 'Armenia'/exp OR 'Bhutan'/exp OR 'Bolivia'/exp OR 'Cameroon'/exp OR 'Cape Verde'/exp OR 'Cote d`Ivoire'/exp OR 'Djibouti'/exp OR 'Egypt'/exp OR 'El Salvador'/exp OR 'Georgia (republic)'/exp OR 'Ghana'/exp OR 'Guatemala'/exp OR 'Guyana'/exp OR 'Honduras'/exp OR 'Indonesia'/exp OR 'India'/exp OR 'Kosovo'/exp OR 'Kyrgyzstan'/exp OR 'Laos'/exp OR 'Lesotho'/exp OR 'Mauritania'/exp OR 'Federated States of Micronesia'/exp OR 'Moldova'/exp OR 'Mongolia'/exp OR 'Nicaragua'/exp OR 'Pakistan'/exp OR 'Papua New Guinea'/exp OR 'Philippines'/exp OR 'Samoa'/exp OR 'Sao Tome and Principe'/exp OR 'Senegal'/exp OR 'Solomon Islands'/exp OR 'Sri Lanka'/exp OR 'Sudan'/exp OR 'Swaziland'/exp OR 'Syrian Arab Republic'/exp OR 'Timor-Leste'/exp OR 'Ukraine'/exp OR 'Uzbekistan'/exp OR 'Vanuatu'/exp OR 'Viet Nam'/exp OR 'Yemen'/exp OR 'Zambia'/exp OR 'Angola'/exp OR 'Albania'/exp OR 'Algeria'/exp OR 'American Samoa'/exp OR 'Argentina'/exp OR 'Azerbaijan'/exp OR 'Belarus'/exp OR 'Belize'/exp OR 'Bosnia and Herzegovina'/exp OR 'Botswana'/exp OR 'Brazil'/exp OR 'Bulgaria'/exp OR 'China'/exp OR 'Colombia'/exp OR 'Costa Rica'/exp OR 'Cuba'/exp OR 'Dominica'/exp OR 'Dominican Republic'/exp OR 'Ecuador'/exp OR 'Equatorial Guinea'/exp OR 'Fiji'/exp OR 'Gabon'/exp OR 'Grenada'/exp OR 'Iran'/exp OR 'Iraq'/exp OR 'Jamaica'/exp OR 'Jordan'/exp OR 'Kazakhstan'/exp OR 'Lebanon'/exp OR 'Libyan Arab Jamahiriya'/exp OR 'Macedonia (republic)'/exp OR 'Malaysia'/exp OR 'Maldives'/exp OR 'Mexico'/exp OR 'Montenegro (republic)'/exp OR 'Namibia'/exp OR 'Palau'/exp OR 'Panama'/exp OR 'Peru'/exp OR 'Romania'/exp OR 'Russian Federation'/exp OR 'Serbia'/exp OR 'Seychelles'/exp OR 'South Africa'/exp OR 'Saint Lucia'/exp OR 'Saint Vincent and the Grenadines'/exp OR 'Suriname'/exp OR 'Thailand'/exp OR 'Tonga'/exp OR 'Tunisia'/exp OR 'Turkey (republic)'/exp OR 'Turkmenistan'/exp OR 'Venezuela'/exp OR 'Afghanistan':ab,ti,ca OR 'Bangladesh':ab,ti,ca OR 'Benin':ab,ti,ca OR 'Burkina Faso':ab,ti,ca OR 'Burundi':ab,ti,ca OR 'Cambodia':ab,ti,ca OR 'cabo verde':ab,ti,ca OR 'Central African Republic':ab,ti,ca OR 'Chad':ab,ti,ca OR 'Comoros':ab,ti,ca OR 'Congo':ab,ti,ca OR 'Eritrea':ab,ti,ca OR 'Ethiopia':ab,ti,ca OR 'Gambia':ab,ti,ca OR 'Guinea':ab,ti,ca OR 'Haiti':ab,ti,ca OR 'Kenya':ab,ti,ca OR 'Korea':ab,ti,ca OR 'Liberia':ab,ti,ca OR 'Madagascar':ab,ti,ca OR 'Malawi':ab,ti,ca OR 'Mali':ab,ti,ca OR 'Mozambique':ab,ti,ca OR 'Myanmar':ab,ti,ca OR 'Nepal':ab,ti,ca OR 'Niger':ab,ti,ca OR 'Rwanda':ab,ti,ca OR 'Sierra Leone':ab,ti,ca OR 'Somalia':ab,ti,ca OR 'Tajikistan':ab,ti,ca OR 'Tanzania':ab,ti,ca OR 'Togo':ab,ti,ca OR 'Uganda':ab,ti,ca OR 'Zimbabwe':ab,ti,ca OR 'Armenia':ab,ti,ca OR 'Bhutan':ab,ti,ca OR 'Bolivia':ab,ti,ca OR 'Cameroon':ab,ti,ca OR 'Cape Verde':ab,ti,ca OR 'Congo':ab,ti,ca OR 'Cote dIvoire':ab,ti,ca OR 'ivory coast':ab,ti,ca OR 'Djibouti':ab,ti,ca OR 'Egypt':ab,ti,ca OR 'El Salvador':ab,ti,ca OR 'Georgia':ab,ti,ca OR 'Ghana':ab,ti,ca OR 'Guatemala':ab,ti,ca OR 'Guyana':ab,ti,ca OR 'Honduras':ab,ti,ca OR 'Indonesia':ab,ti,ca OR 'India':ab,ti,ca OR 'Kiribati':ab,ti,ca OR 'Kosovo':ab,ti,ca OR 'Kyrgyzstan':ab,ti,ca OR 'Kyrgyz':ab,ti,ca OR 'Laos':ab,ti,ca OR 'lao':ab,ti,ca OR 'Lesotho':ab,ti,ca OR 'Mauritania':ab,ti,ca OR 'Micronesia':ab,ti,ca OR 'Moldova':ab,ti,ca OR 'Mongolia':ab,ti,ca OR 'Morocco':ab,ti,ca OR 'Nicaragua':ab,ti,ca OR 'Nigeria':ab,ti,ca OR 'Pakistan':ab,ti,ca OR 'Papua New Guinea':ab,ti,ca OR 'Paraguay':ab,ti,ca OR 'Philippines':ab,ti,ca OR 'Samoa':ab,ti,ca OR 'Atlantic Islands':ab,ti,ca OR 'Sao Tome':ab,ti,ca OR Principe:ab,ti,ca OR 'Senegal':ab,ti,ca OR 'Melanesia':ab,ti,ca OR 'Solomon islands':ab,ti,ca OR 'Sri Lanka':ab,ti,ca OR 'Sudan':ab,ti,ca OR 'Swaziland':ab,ti,ca OR 'Syria':ab,ti,ca OR 'East Timor':ab,ti,ca OR 'Timor leste':ab,ti,ca OR 'Ukraine':ab,ti,ca OR 'Uzbekistan':ab,ti,ca OR 'Vanuatu':ab,ti,ca OR 'Vietnam':ab,ti,ca OR 'Middle East':ab,ti,ca OR 'west bank':ab,ti,ca OR 'Gaza':ab,ti,ca OR 'Yemen':ab,ti,ca OR 'Zambia':ab,ti,ca OR 'Angola':ab,ti,ca OR 'Albania':ab,ti,ca OR 'Algeria':ab,ti,ca OR 'Argentina':ab,ti,ca OR 'Samoa':ab,ti,ca OR 'Azerbaijan':ab,ti,ca OR 'Republic of Belarus':ab,ti,ca OR 'Belize':ab,ti,ca OR Bosnia:ab,ti,ca OR Herzegovina:ab,ti,ca OR 'Botswana':ab,ti,ca OR 'Brazil':ab,ti,ca OR 'Bulgaria':ab,ti,ca OR 'China':ab,ti,ca OR 'Colombia':ab,ti,ca OR 'Costa Rica':ab,ti,ca OR 'Cuba':ab,ti,ca OR 'Dominica':ab,ti,ca OR 'Dominican Republic':ab,ti,ca OR 'Ecuador':ab,ti,ca OR 'Equatorial Guinea':ab,ti,ca OR 'Fiji':ab,ti,ca OR 'Gabon':ab,ti,ca OR 'Grenada':ab,ti,ca OR 'Iran':ab,ti,ca OR 'Iraq':ab,ti,ca OR 'Jamaica':ab,ti,ca OR 'Jordan':ab,ti,ca OR 'Kazakhstan':ab,ti,ca OR 'Lebanon':ab,ti,ca OR 'Libya':ab,ti,ca OR 'Macedonia':ab,ti,ca OR 'Malaysia':ab,ti,ca OR 'Indian Ocean Islands':ab,ti,ca OR 'Maldives':ab,ti,ca OR 'Marshall Islands':ab,ti,ca OR 'Mauritius':ab,ti,ca OR 'Mexico':ab,ti,ca OR 'Montenegro':ab,ti,ca OR 'Namibia':ab,ti,ca OR 'Palau':ab,ti,ca OR 'Panama':ab,ti,ca OR 'Peru':ab,ti,ca OR 'Romania':ab,ti,ca OR 'Russia':ab,ti,ca OR 'Russian Federation':ab,ti,ca OR 'Serbia':ab,ti,ca OR 'Seychelles':ab,ti,ca OR 'South Africa':ab,ti,ca OR 'Saint Lucia':ab,ti,ca OR 'Saint Vincent and the Grenadines':ab,ti,ca OR 'Suriname':ab,ti,ca OR 'Thailand':ab,ti,ca OR 'Tonga':ab,ti,ca OR 'Tunisia':ab,ti,ca OR 'Turkey':ab,ti,ca OR 'Turkmenistan':ab,ti,ca OR 'Tuvalu':ab,ti,ca OR 'Venezuela':ab,ti,ca OR 'low resource':ab,ti OR 'under resourced':ab,ti OR 'resource poor':ab,ti OR 'under developed':ab,ti OR 'underdeveloped':ab,ti OR 'developing country':ab,ti OR 'developing countries':ab,ti OR 'developing world':ab,ti OR 'third world':ab,ti OR lmic:ab,ti OR (low:ab,ti AND middle:ab,ti AND income:ab,ti) | 5059977 |
| #13 | #11 AND #12 | 11957 |
| #14 | [infant]/lim OR [child]/lim OR [adolescent]/lim OR Infancy:ab,ti OR newborn:ab,ti OR newborns:ab,ti OR neonatal:ab,ti OR neonate:ab,ti OR baby:ab,ti OR babies:ab,ti OR toddler:ab,ti OR toddlers:ab,ti OR child:ab,ti OR children:ab,ti OR infant:ab,ti OR infants:ab,ti OR preschool:ab,ti OR preschooler:ab,ti OR pediatric:ab,ti OR teenager:ab,ti OR teenagers:ab,ti OR teenaged:ab,ti OR teen:ab,ti OR teens:ab,ti OR adolescent:ab,ti OR adolescents:ab,ti OR adolescence:ab,ti OR youth:ab,ti OR boy:ab,ti OR boys:ab,ti OR boyhood:ab,ti OR girl:ab,ti OR girls:ab,ti OR girlhood:ab,ti OR kid:ab,ti OR kids:ab,ti OR schoolchild:ab,ti OR "school child":ab,ti OR "school age":ab,ti OR paediatric:ab,ti OR paediatrics:ab,ti | 4160718 |
| #15 | #13 AND #14 | 3811 |
| #16 | ('randomized controlled trial'/exp OR 'crossover procedure'/exp OR 'double blind procedure'/exp OR 'single blind procedure'/exp OR random*:ab,ti OR factorial*:ab,ti OR crossover*:ab,ti OR (cross NEAR/1 over*):ab,ti OR placebo*:ab,ti OR (doubl* NEAR/1 blind*):ab,ti OR (singl* NEAR/1 blind*):ab,ti OR assign*:ab,ti OR allocat*:ab,ti OR volunteer*:ab,ti OR 'clinical study'/exp OR ‘clinical trial’:ti,ab OR ‘clinical trials’:ti,ab OR 'controlled study'/exp OR 'evaluation'/exp OR ‘evaluation study’:ab,ti OR ‘evaluation studies’:ab,ti OR ‘intervention study’:ab,ti OR ‘intervention studies’:ab,ti OR ‘case control’:ab,ti OR 'cohort analysis'/exp OR cohort:ab,ti OR longitudinal*:ab,ti OR prospective:ab,ti OR prospectively:ab,ti OR retrospective:ab,ti OR 'follow up'/exp OR ‘follow up’:ab,ti OR 'comparative effectiveness'/exp OR 'comparative study'/exp OR ‘comparative study’:ab,ti OR ‘comparative studies’:ab,ti OR 'evidence based medicine'/exp OR ‘systematic review’:ab,ti OR ‘meta-analysis’:ab,ti OR ‘meta-analyses’:ab,ti) NOT ('case report'/exp OR 'case study'/exp OR 'editorial'/exp OR 'letter'/exp OR 'note'/exp OR [conference abstract]/lim) | 11195917 |
| #17 | #15 AND #16 | 2361 |
| #18 | #17 AND [embase]/lim NOT [medline]/lim | 660 |
| #19 | #18 AND [1-1-2018]/sd NOT [20-4-2018]/sd | 26 |

Scopus:

|  |  |  |
| --- | --- | --- |
| #1 | TITLE-ABS-KEY("traffic accident'" OR (("motor vehicle" OR traffic OR vehicle OR vehicular OR car OR cars OR automobile OR automobiles OR motorcycle OR motorcycles OR taxi OR cab OR road OR pedestrian OR pedestrians) AND (accident OR accidents OR injury OR injuries))) | 152244 |
| #2 | TITLE-ABS-KEY(Law OR laws OR legal OR regulation OR regulations OR "minimum age" OR (minimum AND (drinking OR alcohol) AND age) OR "seat belt" OR "seat belts" OR restraint OR restraints OR helmet OR helmets OR speed OR safety OR light OR lights OR license OR licenses OR licensing OR prevent*) | 10062205 |
| #3 | TITLE-ABS-KEY(drowning OR drowned OR drowns) | 12833 |
| #4 | TITLE-ABS-KEY(Hazard OR hazards OR fencing OR fence OR fenced OR fences OR flotation OR safety OR resuscitation OR resuscitate OR resuscitated) | 1943054 |
| #5 | TITLE-ABS-KEY(burn OR burns OR (smoke AND inhal*)) | 144287 |
| #6 | TITLE-ABS-KEY("smoke alarm" OR "smoke alarms" OR "child resistant" OR "hot water" OR education OR "burn center" OR "burn centers" OR "burn unit" OR "burn units") | 1928961 |
| #7 | TITLE(falls OR fall) | 49420 |
| #8 | TITLE-ABS-KEY(Furniture OR program OR programs OR intervention OR interventions OR playground OR play OR window OR windows) | 5430372 |
| #9 | TITLE-ABS-KEY(ingestion OR poisoning OR poisoned) | 253226 |
| #10 | TITLE-ABS-KEY("child resistant" OR packaging OR package OR "poison control") | 420011 |
| #11 | (#1 AND #2) OR (#3 AND #4) OR (#5 AND #6) OR (#7 AND #8) OR (#9 AND #10) | 100008 |
| #12 | TITLE-ABS-KEY("Afghanistan" OR "Bangladesh" OR "Benin" OR "Burkina Faso" OR "Burundi" OR "Cambodia" OR "cabo verde" OR "Central African Republic" OR "Chad" OR "Comoros" OR "Congo" OR "Eritrea" OR "Ethiopia" OR "Gambia" OR "Guinea" OR "Haiti" OR "Kenya" OR "Korea" OR "Liberia" OR "Madagascar" OR "Malawi" OR "Mali" OR "Mozambique" OR "Myanmar" OR "Nepal" OR "Niger" OR "Rwanda" OR "Sierra Leone" OR "Somalia" OR "Tajikistan" OR "Tanzania" OR "Togo" OR "Uganda" OR "Zimbabwe" OR "Armenia" OR "Bhutan" OR "Bolivia" OR "Cameroon" OR "Cape Verde" OR "Congo" OR "Cote dIvoire" OR "ivory coast" OR "Djibouti" OR "Egypt" OR "El Salvador" OR "Georgia" OR "Ghana" OR "Guatemala" OR "Guyana" OR "Honduras" OR "Indonesia" OR "India" OR "Kiribati" OR "Kosovo" OR "Kyrgyzstan" OR "Kyrgyz" OR "Laos" OR "lao" OR "Lesotho" OR "Mauritania" OR "Micronesia" OR "Moldova" OR "Mongolia" OR "Morocco" OR "Nicaragua" OR "Nigeria" OR "Pakistan" OR "Papua New Guinea" OR "Paraguay" OR "Philippines" OR "Samoa" OR "Atlantic Islands" OR "Sao Tome" OR  Principe OR "Senegal" OR "Melanesia" OR "Solomon islands" OR "Sri Lanka" OR "Sudan" OR "Swaziland" OR "Syria" OR "East Timor" OR "Timor leste" OR "Ukraine" OR "Uzbekistan" OR "Vanuatu" OR "Vietnam" OR "Middle East" OR "west bank" OR "Gaza" OR "Yemen" OR "Zambia" OR "Angola" OR "Albania" OR "Algeria" OR "Argentina" OR "Samoa" OR "Azerbaijan" OR "Republic of Belarus" OR "Belize" OR Bosnia OR Herzegovina OR "Botswana" OR "Brazil" OR "Bulgaria" OR "China" OR "Colombia" OR "Costa Rica" OR "Cuba" OR "Dominica" OR "Dominican Republic" OR "Ecuador" OR "Equatorial Guinea" OR "Fiji" OR "Gabon" OR "Grenada" OR "Iran" OR "Iraq" OR "Jamaica" OR "Jordan" OR "Kazakhstan" OR "Lebanon" OR "Libya" OR "Macedonia" OR "Malaysia" OR "Indian Ocean Islands" OR "Maldives" OR "Marshall Islands" OR "Mauritius" OR "Mexico" OR "Montenegro" OR "Namibia" OR "Palau" OR "Panama" OR "Peru" OR "Romania" OR "Russia" OR "Russian Federation" OR "Serbia" OR "Seychelles" OR "South Africa" OR "Saint Lucia" OR "Saint Vincent and the Grenadines" OR "Suriname" OR "Thailand" OR "Tonga" OR "Tunisia" OR "Turkey" OR "Turkmenistan" OR "Tuvalu" OR "Venezuela" OR "low resource" OR "under resourced" OR "resource poor" OR "under developed" OR "underdeveloped" OR "developing country" OR "developing countries" OR "developing world" OR "third world" OR lmic OR (low AND middle AND income)) OR AFFILCOUNTRY("Afghanistan" OR "Bangladesh" OR "Benin" OR "Burkina Faso" OR "Burundi" OR "Cambodia" OR "cabo verde" OR "Central African Republic" OR "Chad" OR "Comoros" OR "Congo" OR "Eritrea" OR "Ethiopia" OR "Gambia" OR "Guinea" OR "Haiti" OR "Kenya" OR "Korea" OR "Liberia" OR "Madagascar" OR "Malawi" OR "Mali" OR "Mozambique" OR "Myanmar" OR "Nepal" OR "Niger" OR "Rwanda" OR "Sierra Leone" OR "Somalia" OR "Tajikistan" OR "Tanzania" OR "Togo" OR "Uganda" OR "Zimbabwe" OR "Armenia" OR "Bhutan" OR "Bolivia" OR "Cameroon" OR "Cape Verde" OR "Congo" OR "Cote dIvoire" OR "ivory coast" OR "Djibouti" OR "Egypt" OR "El Salvador" OR "Georgia" OR "Ghana" OR "Guatemala" OR "Guyana" OR "Honduras" OR "Indonesia" OR "India" OR "Kiribati" OR "Kosovo" OR "Kyrgyzstan" OR "Kyrgyz" OR "Laos" OR "lao" OR "Lesotho" OR "Mauritania" OR "Micronesia" OR "Moldova" OR "Mongolia" OR "Morocco" OR "Nicaragua" OR "Nigeria" OR "Pakistan" OR "Papua New Guinea" OR "Paraguay" OR "Philippines" OR "Samoa" OR "Atlantic Islands" OR "Sao Tome" OR  Principe OR "Senegal" OR "Melanesia" OR "Solomon islands" OR "Sri Lanka" OR "Sudan" OR "Swaziland" OR "Syria" OR "East Timor" OR "Timor leste" OR "Ukraine" OR "Uzbekistan" OR "Vanuatu" OR "Vietnam" OR "Middle East" OR "west bank" OR "Gaza" OR "Yemen" OR "Zambia" OR "Angola" OR "Albania" OR "Algeria" OR "Argentina" OR "Samoa" OR "Azerbaijan" OR "Republic of Belarus" OR "Belize" OR Bosnia OR Herzegovina OR "Botswana" OR "Brazil" OR "Bulgaria" OR "China" OR "Colombia" OR "Costa Rica" OR "Cuba" OR "Dominica" OR "Dominican Republic" OR "Ecuador" OR "Equatorial Guinea" OR "Fiji" OR "Gabon" OR "Grenada" OR "Iran" OR "Iraq" OR "Jamaica" OR "Jordan" OR "Kazakhstan" OR "Lebanon" OR "Libya" OR "Macedonia" OR "Malaysia" OR "Indian Ocean Islands" OR "Maldives" OR "Marshall Islands" OR "Mauritius" OR "Mexico" OR "Montenegro" OR "Namibia" OR "Palau" OR "Panama" OR "Peru" OR "Romania" OR "Russia" OR "Russian Federation" OR "Serbia" OR "Seychelles" OR "South Africa" OR "Saint Lucia" OR "Saint Vincent and the Grenadines" OR "Suriname" OR "Thailand" OR "Tonga" OR "Tunisia" OR "Turkey" OR "Turkmenistan" OR "Tuvalu" OR "Venezuela" OR "low resource" OR "under resourced" OR "resource poor" OR "under developed" OR "underdeveloped" OR "developing country" OR "developing countries" OR "developing world" OR "third world" OR lmic OR (low AND middle AND income)) | 18193263 |
| #13 | #11 AND #12 | 23882 |
| #14 | TITLE-ABS-KEY(Infancy OR newborn OR newborns OR neonatal OR neonate OR baby OR babies OR toddler OR toddlers OR child OR children OR infant OR infants OR preschool OR preschooler OR pediatric OR teenager OR teenagers OR teenaged OR teen OR teens OR adolescent OR adolescents OR adolescence OR youth OR boy OR boys OR boyhood OR girl OR girls OR girlhood OR kid OR kids OR schoolchild OR "school child" OR "school age" OR paediatric OR paediatrics) | 5067977 |
| #15 | #13 AND #14 | 4311 |
| #16 | TITLE-ABS-KEY( ((random OR randomly OR randomized OR factorial OR crossover) AND (trial OR study)) OR "clinical study" OR "clinical trial" OR "clinical trials" OR "controlled study" OR "evaluation study" OR "evaluation studies" OR "intervention study" OR "intervention studies" OR "case control" OR "cohort analysis" OR cohort OR longitudinal OR longitudinally OR prospective OR prospectively OR retrospective OR "follow up" OR "comparative effectiveness" OR "comparative study" OR "comparative studies" OR "systematic review" OR "meta-analysis" OR "meta-analyses") | 11945103 |
| #17 | #15 AND #16 | 2602 |
| #18 | #17, exclude conference papers and letters, limit to 2018 and 2019 | 178 |

Global Health Library

(tw:((injury OR accident OR accidents OR drowning OR burns OR poisoning) AND prevent* AND (child OR children OR pediatric OR paediatric OR youth) AND (program OR intervention OR study OR trial)))

Limit to:

LILACS, WPRIM, IMEMR, IMSEAR, WHOLIS, AIM

= 40 citations

**B3: Third Search Terms**

PubMed

|  |  |  |
| --- | --- | --- |
| #1 | "Accidents, Traffic"[Mesh] OR (("Motor Vehicles"[Mesh:NoExp] OR "Automobiles"[Mesh] OR "Motorcycles"[Mesh] OR traffic[tiab] OR vehicle[tiab] OR vehicular[tiab] OR car[tiab] OR cars[tiab] OR automobile[tiab] OR automobiles[tiab] OR motorcycle[tiab] OR motorcycles[tiab] OR taxi[tiab] OR cab[tiab] OR road[tiab] OR pedestrian[tiab] OR pedestrians[tiab]) AND (accident[tiab] OR accidents[tiab] OR injury[tiab] OR injuries[tiab] OR "Wounds and Injuries"[Mesh] OR "injuries" [Subheading])) | 75,158 |
| #2 | "Accidents, Traffic/prevention and control"[Mesh] OR "Social Control, Formal"[Mesh] OR "Protective Devices"[Mesh] OR "organization and administration" [Subheading] OR "Safety"[Mesh] OR Law[tiab] OR laws[tiab] OR legal[tiab] OR regulation[tiab] OR regulations[tiab] OR "minimum age"[tiab] OR (minimum[tiab] AND (drinking[tiab] OR alcohol[tiab]) AND age[tiab]) OR "seat belt"[tiab] OR "seat belts"[tiab] OR restraint[tiab] OR restraints[tiab] OR helmet[tiab] OR helmets[tiab] OR speed[tiab] OR safety[tiab] OR light[tiab] OR lights[tiab] OR license[tiab] OR licenses[tiab] OR licensing[tiab] OR prevent*[tiab] | 5,579,477 |
| #3 | "Drowning"[Mesh] OR drowning[tiab] OR drowned[tiab] OR drowns[tiab] | 6,151 |
| #4 | "Drowning/prevention and control"[Mesh] OR "Protective Devices"[Mesh] OR "Resuscitation"[Mesh] OR Hazard[tiab] OR hazards[tiab] OR fencing[tiab] OR fence[tiab] OR fenced[tiab] OR fences[tiab] OR flotation[tiab] OR safety[tiab] OR resuscitation[tiab] OR resuscitate[tiab] OR resuscitated[tiab] | 873,089 |
| #5 | "Burns"[Mesh] OR "Smoke Inhalation Injury"[Mesh] OR burn[tiab] OR burns[tiab] OR (smoke[tiab] AND inhal*[tiab]) | 80,758 |
| #6 | "Burns/prevention and control"[Mesh] OR "Fires/prevention and control"[Mesh] OR "Protective Devices"[Mesh] OR "Hot Temperature"[Mesh] OR "Health Education"[Mesh] OR "Burn Units"[Mesh] OR "smoke alarm"[tiab] OR "smoke alarms"[tiab] OR "child resistant"[tiab] OR "hot water"[tiab] OR education[tiab] OR "burn center"[tiab] OR "burn centers"[tiab] OR "burn unit"[tiab] OR "burn units"[tiab] | 816,067 |
| #7 | "Accidental Falls"[Mesh] OR falls[ti] OR fall[ti] | 31,858 |
| #8 | "Accidental Falls/prevention and control"[Mesh] OR "Interior Design and Furnishings"[Mesh] OR "Infant Equipment"[Mesh] OR "Protective Devices"[Mesh] OR Furniture[tiab] OR program[tiab] OR programs[tiab] OR intervention[tiab] OR interventions[tiab] OR playground[tiab] OR play[tiab] OR window[tiab] OR windows[tiab] | 2,238,712 |
| #9 | "Poisoning"[Mesh] OR "poisoning" [Subheading] OR ingestion[tiab] OR poisoning[tiab] OR poisoned[tiab] | 286,311 |
| #10 | "Poisoning/prevention and control"[Mesh] OR "Drug Packaging"[Mesh] OR "Poison Control Centers"[Mesh] OR "child resistant"[tiab] OR packaging[tiab] OR package[tiab] OR "poison control"[tiab] | 89,608 |
| #11 | (#1 AND #2) OR (#3 AND #4) OR (#5 AND #6) OR (#7 AND #8) OR (#9 AND #10) | 75411 |
| #12 | "Developing Countries"[Mesh] OR "Afghanistan"[Mesh] OR "Bangladesh"[Mesh] OR "Benin"[Mesh] OR "Burkina Faso"[Mesh] OR "Burundi"[Mesh] OR "Cambodia"[Mesh] OR "Central African Republic"[Mesh] OR "Chad"[Mesh] OR "Comoros"[Mesh] OR "Democratic Republic of the Congo"[Mesh] OR "Eritrea"[Mesh] OR "Ethiopia"[Mesh] OR "Gambia"[Mesh] OR "Guinea"[Mesh] OR "Guinea-Bissau"[Mesh] OR "Haiti"[Mesh] OR "Kenya"[Mesh] OR "Democratic People's Republic of Korea"[Mesh] OR "Liberia"[Mesh] OR "Madagascar"[Mesh] OR "Malawi"[Mesh] OR "Mali"[Mesh] OR "Mozambique"[Mesh] OR "Myanmar"[Mesh] OR "Nepal"[Mesh] OR "Niger"[Mesh] OR "Rwanda"[Mesh] OR "Sierra Leone"[Mesh] OR "Somalia"[Mesh] OR "Tajikistan"[Mesh] OR "Tanzania"[Mesh] OR "Togo"[Mesh] OR "Uganda"[Mesh] OR "Zimbabwe"[Mesh] OR "Armenia"[Mesh] OR "Bhutan"[Mesh] OR "Bolivia"[Mesh] OR "Cameroon"[Mesh] OR "Cabo Verde"[Mesh] OR "Congo"[Mesh] OR "Cote d'Ivoire"[Mesh] OR "Djibouti"[Mesh] OR "Egypt"[Mesh] OR "El Salvador"[Mesh] OR "Georgia (Republic)"[Mesh] OR "Ghana"[Mesh] OR "Guatemala"[Mesh] OR "Guyana"[Mesh] OR "Honduras"[Mesh] OR "Indonesia"[Mesh] OR "India"[Mesh] OR "Kosovo"[Mesh] OR "Kyrgyzstan"[Mesh] OR "Laos"[Mesh] OR "Lesotho"[Mesh] OR "Mauritania"[Mesh] OR "Micronesia"[Mesh] OR "Moldova"[Mesh] OR "Mongolia"[Mesh] OR "Morocco"[Mesh] OR "Nicaragua"[Mesh] OR "Nigeria"[Mesh] OR "Pakistan"[Mesh] OR "Papua New Guinea"[Mesh] OR "Paraguay"[Mesh] OR "Philippines"[Mesh] OR "Independent State of Samoa"[Mesh] OR "Atlantic Islands"[Mesh] OR "Senegal"[Mesh] OR "Melanesia"[Mesh] OR "Sri Lanka"[Mesh] OR "Sudan"[Mesh] OR "Eswatini"[Mesh] OR "Syria"[Mesh] OR "Timor-Leste"[Mesh] OR "Ukraine"[Mesh] OR "Uzbekistan"[Mesh] OR "Vanuatu"[Mesh] OR "Vietnam"[Mesh] OR "Middle East"[Mesh] OR "Yemen"[Mesh] OR "Zambia"[Mesh] OR "Angola"[Mesh] OR "Albania"[Mesh] OR "Algeria"[Mesh] OR "American Samoa"[Mesh] OR "Argentina"[Mesh] OR "Azerbaijan"[Mesh] OR "Republic of Belarus"[Mesh] OR "Belize"[Mesh] OR "Bosnia and Herzegovina"[Mesh] OR "Botswana"[Mesh] OR "Brazil"[Mesh] OR "Bulgaria"[Mesh] OR "China"[Mesh] OR "Colombia"[Mesh] OR "Costa Rica"[Mesh] OR "Cuba"[Mesh] OR "Dominica"[Mesh] OR "Dominican Republic"[Mesh] OR "Ecuador"[Mesh] OR "Equatorial Guinea"[Mesh] OR "Fiji"[Mesh] OR "Gabon"[Mesh] OR "Grenada"[Mesh] OR "Iran"[Mesh] OR "Iraq"[Mesh] OR "Jamaica"[Mesh] OR "Jordan"[Mesh] OR "Kazakhstan"[Mesh] OR "Lebanon"[Mesh] OR "Libya"[Mesh] OR "Republic of North Macedonia"[Mesh] OR "Malaysia"[Mesh] OR "Indian Ocean Islands"[Mesh] OR "Mexico"[Mesh] OR "Montenegro"[Mesh] OR "Namibia"[Mesh] OR "Palau"[Mesh] OR "Panama"[Mesh] OR "Peru"[Mesh] OR "Romania"[Mesh] OR "Russia"[Mesh] OR "Serbia"[Mesh] OR "Seychelles"[Mesh] OR "South Africa"[Mesh] OR "Saint Lucia"[Mesh] OR "Saint Vincent and the Grenadines"[Mesh] OR "Suriname"[Mesh] OR "Thailand"[Mesh] OR "Tonga"[Mesh] OR "Tunisia"[Mesh] OR "Turkey"[Mesh] OR "Turkmenistan"[Mesh] OR "Venezuela"[Mesh] OR "Afghanistan"[all fields] OR "Bangladesh"[all fields] OR "Benin"[all fields] OR "Burkina Faso"[all fields] OR "Burundi"[all fields] OR "Cambodia"[all fields] OR "cabo verde"[all fields] OR "Central African Republic"[all fields] OR "Chad"[all fields] OR "Comoros"[all fields] OR "Democratic Republic of the Congo"[all fields] OR "Eritrea"[all fields] OR "Eswatini"[all fields] OR "Ethiopia"[all fields] OR "Gambia"[all fields] OR "Guinea"[all fields] OR "Guinea-Bissau"[all fields] OR "Haiti"[all fields] OR "Kenya"[all fields] OR "Democratic People's Republic of Korea"[all fields] OR "Liberia"[all fields] OR "Madagascar"[all fields] OR "Malawi"[all fields] OR "Mali"[all fields] OR "Mozambique"[all fields] OR "Myanmar"[all fields] OR "Nepal"[all fields] OR "Niger"[all fields] OR "Rwanda"[all fields] OR "Sierra Leone"[all fields] OR "Somalia"[all fields] OR "Tajikistan"[all fields] OR "Tanzania"[all fields] OR "Togo"[all fields] OR "Uganda"[all fields] OR "Zimbabwe"[all fields] OR "Armenia"[all fields] OR "Bhutan"[all fields] OR "Bolivia"[all fields] OR "Cameroon"[all fields] OR "Cape Verde"[all fields] OR "Congo"[all fields] OR "Cote d'Ivoire"[all fields] OR "Djibouti"[all fields] OR "Egypt"[all fields] OR "El Salvador"[all fields] OR "Georgia (Republic)"[all fields] OR "Ghana"[all fields] OR "Guatemala"[all fields] OR "Guyana"[all fields] OR "Honduras"[all fields] OR "Indonesia"[all fields] OR "India"[all fields] OR “Kiribati”[all fields] OR "Kosovo"[all fields] OR "Kyrgyzstan"[all fields] OR "Kyrgyz"[all fields] OR "Laos"[all fields] OR "lao"[all fields] OR "Lesotho"[all fields] OR "Mauritania"[all fields] OR "Micronesia"[all fields] OR "Moldova"[all fields] OR "Mongolia"[all fields] OR "Morocco"[all fields] OR "Nicaragua"[all fields] OR "Nigeria"[all fields] OR "Pakistan"[all fields] OR "Papua New Guinea"[all fields] OR "Paraguay"[all fields] OR "Philippines"[all fields] OR "Independent State of Samoa"[all fields] OR "Atlantic Islands"[all fields] OR "Sao Tome"[all fields] OR Principe[all fields] OR "Senegal"[all fields] OR "Melanesia"[all fields] OR "Solomon islands"[all fields] OR "Sri Lanka"[all fields] OR "Sudan"[all fields] OR "Swaziland"[all fields] OR "Syria"[all fields] OR "East Timor"[all fields] OR "Timor leste"[all fields] OR "Ukraine"[all fields] OR "Uzbekistan"[all fields] OR "Vanuatu"[all fields] OR "Vietnam"[all fields] OR "Middle East"[all fields] OR "west bank"[all fields] OR "Gaza"[all fields] OR "Yemen"[all fields] OR "Zambia"[all fields] OR "Angola"[all fields] OR "Albania"[all fields] OR "Algeria"[all fields] OR "Argentina"[all fields] OR "Samoa"[all fields] OR "Azerbaijan"[all fields] OR "Republic of Belarus"[all fields] OR "Belize"[all fields] OR "Bosnia-Herzegovina"[all fields] OR "Botswana"[all fields] OR "Brazil"[all fields] OR "Bulgaria"[all fields] OR "China"[all fields] OR "Colombia"[all fields] OR "Costa Rica"[all fields] OR "Cuba"[all fields] OR "Dominica"[all fields] OR "Dominican Republic"[all fields] OR "Ecuador"[all fields] OR "Equatorial Guinea"[all fields] OR "Fiji"[all fields] OR "Gabon"[all fields] OR "Grenada"[all fields] OR "Iran"[all fields] OR "Iraq"[all fields] OR "Jamaica"[all fields] OR "Jordan"[all fields] OR "Kazakhstan"[all fields] OR "Lebanon"[all fields] OR "Libya"[all fields] OR "Macedonia"[all fields] OR "Malaysia"[all fields] OR "Indian Ocean Islands"[all fields] OR "Maldives"[all fields] OR “Marshall Islands”[all fields] OR "Mauritius"[all fields] OR "Mexico"[all fields] OR "Montenegro"[all fields] OR "Namibia"[all fields] OR "Palau"[all fields] OR "Panama"[all fields] OR "Peru"[all fields] OR "Romania"[all fields] OR "Russia"[all fields] OR "Russian Federation"[all fields] OR "Serbia"[all fields] OR "Seychelles"[all fields] OR "South Africa"[all fields] OR "Saint Lucia"[all fields] OR "Saint Vincent and the Grenadines"[all fields] OR "Suriname"[all fields] OR "Thailand"[all fields] OR "Tonga"[all fields] OR "Tunisia"[all fields] OR "Turkey"[all fields] OR "Turkmenistan"[all fields] OR "Tuvalu"[all fields] OR "Venezuela"[all fields] OR "low resource"[all fields] OR "under-resourced"[all fields] OR "resource poor"[all fields] OR "under-developed"[all fields] OR "underdeveloped"[all fields] OR "developing country"[all fields] OR "developing countries"[all fields] OR "developing world"[all fields] OR "third world" [all fields] OR lmic[all fields] OR (low[all fields] AND middle[all fields] AND income[all fields]) | 4,767,649 |
| #13 | #11 AND #12 | 14,065 |
| #14 | "Pediatrics"[Mesh] OR "Adolescent"[Mesh] OR "Infant"[Mesh] OR "Child"[Mesh] OR Infancy[tiab] OR newborn[tiab] OR newborns[tiab] OR neonatal[tiab] OR neonate[tiab] OR baby[tiab] OR babies[tiab] OR toddler[tiab] OR toddlers[tiab] OR child[tiab] OR children[tiab] OR infant[tiab] OR infants[tiab] OR preschool[tiab] OR preschooler[tiab] OR pediatric[tiab] OR teenager[tiab] OR teenagers[tiab] OR teenaged[tiab] OR teen[tiab] OR teens[tiab] OR adolescent[tiab] OR adolescents[tiab] OR adolescence[tiab] OR youth[tiab] OR boy[tiab] OR boys[tiab] OR boyhood[tiab] OR girl[tiab] OR girls[tiab] OR girlhood[tiab] OR kid[tiab] OR kids[tiab] OR schoolchild[tiab] OR "school child"[tiab] OR "school age"[tiab] OR paediatric[tiab] OR paediatrics[tiab] | 4,157,868 |
| #15 | #13 AND #14 | 4,222 |
| #16 | (randomized controlled trial[pt] OR controlled clinical trial[pt] OR randomized[tiab] OR randomised[tiab] OR randomization[tiab] OR randomisation[tiab] OR placebo[tiab] OR drug therapy[sh] OR randomly[tiab] OR trial[tiab] OR groups[tiab] OR Clinical trial[pt] OR “clinical trial”[tiab] OR “clinical trials”[tiab] OR "evaluation studies"[Publication Type] OR "evaluation studies as topic"[MeSH Terms] OR "evaluation study"[tiab] OR evaluation studies[tiab] OR "intervention study"[tiab] OR "intervention studies"[tiab] OR "case-control studies"[MeSH Terms] OR "case-control"[tiab] OR "cohort studies"[MeSH Terms] OR cohort[tiab] OR "longitudinal studies"[MeSH Terms] OR "longitudinal”[tiab] OR longitudinally[tiab] OR "prospective"[tiab] OR prospectively[tiab] OR "retrospective studies"[MeSH Terms] OR "retrospective"[tiab] OR "follow up"[tiab] OR "comparative study"[Publication Type] OR "comparative study"[tiab] OR systematic[subset] OR "meta-analysis"[Publication Type] OR "meta-analysis as topic"[MeSH Terms] OR "meta-analysis"[tiab] OR "meta-analyses"[tiab]) NOT (Editorial[ptyp] OR Letter[ptyp] OR Case Reports[ptyp] OR Comment[ptyp]) NOT (animals[mh] NOT humans[mh]) | 7,085,254 |
| #17 | #15 AND #16 | 2,374 |
| #18 | "2019/04/01"[Date - Entrez] : "3000"[Date - Entrez] AND #17 | 116 |

Embase:

| #1 | 'traffic accident'/exp OR (('motor vehicle'/exp OR traffic:ab,ti OR vehicle:ab,ti OR vehicular:ab,ti OR car:ab,ti OR cars:ab,ti OR automobile:ab,ti OR automobiles:ab,ti OR motorcycle:ab,ti OR motorcycles:ab,ti OR taxi:ab,ti OR cab:ab,ti OR road:ab,ti OR pedestrian:ab,ti OR pedestrians:ab,ti) AND (accident:ab,ti OR accidents:ab,ti OR injury:ab,ti OR injuries:ab,ti OR 'injury'/exp)) | [109,618](https://www-embase-com.proxy.lib.duke.edu/) |
| --- | --- | --- |
| #2 | 'legal aspect'/exp OR 'helmet'/exp OR 'traffic safety'/exp OR 'driver licence'/exp OR Law:ab,ti OR laws:ab,ti OR legal:ab,ti OR regulation:ab,ti OR regulations:ab,ti OR "minimum age":ab,ti OR (minimum:ab,ti AND (drinking:ab,ti OR alcohol:ab,ti) AND age:ab,ti) OR "seat belt":ab,ti OR "seat belts":ab,ti OR restraint:ab,ti OR restraints:ab,ti OR helmet:ab,ti OR helmets:ab,ti OR speed:ab,ti OR safety:ab,ti OR light:ab,ti OR lights:ab,ti OR license:ab,ti OR licenses:ab,ti OR licensing:ab,ti OR prevent*:ab,ti | [5,541,744](https://www-embase-com.proxy.lib.duke.edu/) |
| #3 | 'drowning'/exp OR drowning:ab,ti OR drowned:ab,ti OR drowns:ab,ti | [8,708](https://www-embase-com.proxy.lib.duke.edu/) |
| #4 | 'drowning'/exp/dm_pc OR 'protective equipment'/exp OR 'resuscitation'/exp OR Hazard:ab,ti OR hazards:ab,ti OR fencing:ab,ti OR fence:ab,ti OR fenced:ab,ti OR fences:ab,ti OR flotation:ab,ti OR safety:ab,ti OR resuscitation:ab,ti OR resuscitate:ab,ti OR resuscitated:ab,ti | [1,276,273](https://www-embase-com.proxy.lib.duke.edu/) |
| #5 | 'burn'/exp OR burn:ab,ti OR burns:ab,ti OR (smoke:ab,ti AND inhal*:ab,ti) | [113,096](https://www-embase-com.proxy.lib.duke.edu/) |
| #6 | 'burn'/exp/dm_pc OR 'fire protection'/exp OR 'protective equipment'/exp OR 'health promotion'/exp OR 'burn unit'/exp OR "smoke alarm":ab,ti OR "smoke alarms":ab,ti OR "child resistant":ab,ti OR "hot water":ab,ti OR education:ab,ti OR "burn center":ab,ti OR "burn centers":ab,ti OR "burn unit":ab,ti OR "burn units":ab,ti | [755,001](https://www-embase-com.proxy.lib.duke.edu/) |
| #7 | 'falling'/exp OR falls:ti OR fall:ti | [52,046](https://www-embase-com.proxy.lib.duke.edu/) |
| #8 | 'furniture'/exp OR 'infant equipment'/exp OR 'protective equipment'/exp OR Furniture:ab,ti OR program:ab,ti OR programs:ab,ti OR intervention:ab,ti OR interventions:ab,ti OR playground:ab,ti OR play:ab,ti OR window:ab,ti OR windows:ab,ti | [3,010,115](https://www-embase-com.proxy.lib.duke.edu/) |
| #9 | 'intoxication'/exp OR ingestion:ab,ti OR poisoning:ab,ti OR poisoned:ab,ti | [538,660](https://www-embase-com.proxy.lib.duke.edu/) |
| #10 | 'intoxication'/exp/dm_pc OR 'drug packaging'/exp OR 'poison center'/exp OR "child resistant":ab,ti OR packaging:ab,ti OR package:ab,ti OR "poison control":ab,ti | [107,395](https://www-embase-com.proxy.lib.duke.edu/) |
| #11 | (#1 AND #2) OR (#3 AND #4) OR (#5 AND #6) OR (#7 AND #8) OR (#9 AND #10) | [80,632](https://www-embase-com.proxy.lib.duke.edu/) |
| #12 | 'developing country'/exp OR 'Afghanistan'/exp OR 'Bangladesh'/exp OR 'Benin'/exp OR 'Burkina Faso'/exp OR 'Burundi'/exp OR 'Cambodia'/exp OR 'Central African Republic'/exp OR 'Chad'/exp OR 'Comoros'/exp OR 'Democratic Republic Congo'/exp OR 'Congo'/exp OR 'Eritrea'/exp OR 'Ethiopia'/exp OR 'Gambia'/exp OR 'Guinea'/exp OR 'Guinea-Bissau'/exp OR 'Haiti'/exp OR 'Kenya'/exp OR 'North Korea'/exp OR 'Liberia'/exp OR 'Madagascar'/exp OR 'Malawi'/exp OR 'Mozambique'/exp OR 'Myanmar'/exp OR 'Nepal'/exp OR 'Niger'/exp OR 'Nigeria'/exp OR 'Rwanda'/exp OR 'Sierra Leone'/exp OR 'Somalia'/exp OR 'Tajikistan'/exp OR 'Tanzania'/exp OR 'Togo'/exp OR 'Uganda'/exp OR 'Zimbabwe'/exp OR 'Armenia'/exp OR 'Bhutan'/exp OR 'Bolivia'/exp OR 'Cameroon'/exp OR 'Cape Verde'/exp OR 'Cote d`Ivoire'/exp OR 'Djibouti'/exp OR 'Egypt'/exp OR 'El Salvador'/exp OR 'Georgia (republic)'/exp OR 'Ghana'/exp OR 'Guatemala'/exp OR 'Guyana'/exp OR 'Honduras'/exp OR 'Indonesia'/exp OR 'India'/exp OR 'Kosovo'/exp OR 'Kyrgyzstan'/exp OR 'Laos'/exp OR 'Lesotho'/exp OR 'Mauritania'/exp OR 'Federated States of Micronesia'/exp OR 'Moldova'/exp OR 'Mongolia'/exp OR 'Nicaragua'/exp OR 'Pakistan'/exp OR 'Papua New Guinea'/exp OR 'Philippines'/exp OR 'Samoa'/exp OR 'Sao Tome and Principe'/exp OR 'Senegal'/exp OR 'Solomon Islands'/exp OR 'Sri Lanka'/exp OR 'Sudan'/exp OR 'Swaziland'/exp OR 'Syrian Arab Republic'/exp OR 'Timor-Leste'/exp OR 'Ukraine'/exp OR 'Uzbekistan'/exp OR 'Vanuatu'/exp OR 'Viet Nam'/exp OR 'Yemen'/exp OR 'Zambia'/exp OR 'Angola'/exp OR 'Albania'/exp OR 'Algeria'/exp OR 'American Samoa'/exp OR 'Argentina'/exp OR 'Azerbaijan'/exp OR 'Belarus'/exp OR 'Belize'/exp OR 'Bosnia and Herzegovina'/exp OR 'Botswana'/exp OR 'Brazil'/exp OR 'Bulgaria'/exp OR 'China'/exp OR 'Colombia'/exp OR 'Costa Rica'/exp OR 'Cuba'/exp OR 'Dominica'/exp OR 'Dominican Republic'/exp OR 'Ecuador'/exp OR 'Equatorial Guinea'/exp OR 'Fiji'/exp OR 'Gabon'/exp OR 'Grenada'/exp OR 'Iran'/exp OR 'Iraq'/exp OR 'Jamaica'/exp OR 'Jordan'/exp OR 'Kazakhstan'/exp OR 'Lebanon'/exp OR 'Libyan Arab Jamahiriya'/exp OR 'Macedonia (republic)'/exp OR 'Malaysia'/exp OR 'Maldives'/exp OR 'Mexico'/exp OR 'Montenegro (republic)'/exp OR 'Namibia'/exp OR 'Palau'/exp OR 'Panama'/exp OR 'Peru'/exp OR 'Romania'/exp OR 'Russian Federation'/exp OR 'Serbia'/exp OR 'Seychelles'/exp OR 'South Africa'/exp OR 'Saint Lucia'/exp OR 'Saint Vincent and the Grenadines'/exp OR 'Suriname'/exp OR 'Thailand'/exp OR 'Tonga'/exp OR 'Tunisia'/exp OR 'Turkey (republic)'/exp OR 'Turkmenistan'/exp OR 'Venezuela'/exp OR 'Afghanistan':ab,ti,ca OR 'Bangladesh':ab,ti,ca OR 'Benin':ab,ti,ca OR 'Burkina Faso':ab,ti,ca OR 'Burundi':ab,ti,ca OR 'Cambodia':ab,ti,ca OR 'cabo verde':ab,ti,ca OR 'Central African Republic':ab,ti,ca OR 'Chad':ab,ti,ca OR 'Comoros':ab,ti,ca OR 'Congo':ab,ti,ca OR 'Eritrea':ab,ti,ca OR 'Ethiopia':ab,ti,ca OR 'Gambia':ab,ti,ca OR 'Guinea':ab,ti,ca OR 'Haiti':ab,ti,ca OR 'Kenya':ab,ti,ca OR 'Korea':ab,ti,ca OR 'Liberia':ab,ti,ca OR 'Madagascar':ab,ti,ca OR 'Malawi':ab,ti,ca OR 'Mali':ab,ti,ca OR 'Mozambique':ab,ti,ca OR 'Myanmar':ab,ti,ca OR 'Nepal':ab,ti,ca OR 'Niger':ab,ti,ca OR 'Rwanda':ab,ti,ca OR 'Sierra Leone':ab,ti,ca OR 'Somalia':ab,ti,ca OR 'Tajikistan':ab,ti,ca OR 'Tanzania':ab,ti,ca OR 'Togo':ab,ti,ca OR 'Uganda':ab,ti,ca OR 'Zimbabwe':ab,ti,ca OR 'Armenia':ab,ti,ca OR 'Bhutan':ab,ti,ca OR 'Bolivia':ab,ti,ca OR 'Cameroon':ab,ti,ca OR 'Cape Verde':ab,ti,ca OR 'Congo':ab,ti,ca OR 'Cote dIvoire':ab,ti,ca OR 'ivory coast':ab,ti,ca OR 'Djibouti':ab,ti,ca OR 'Egypt':ab,ti,ca OR 'El Salvador':ab,ti,ca OR 'Georgia':ab,ti,ca OR 'Ghana':ab,ti,ca OR 'Guatemala':ab,ti,ca OR 'Guyana':ab,ti,ca OR 'Honduras':ab,ti,ca OR 'Indonesia':ab,ti,ca OR 'India':ab,ti,ca OR 'Kiribati':ab,ti,ca OR 'Kosovo':ab,ti,ca OR 'Kyrgyzstan':ab,ti,ca OR 'Kyrgyz':ab,ti,ca OR 'Laos':ab,ti,ca OR 'lao':ab,ti,ca OR 'Lesotho':ab,ti,ca OR 'Mauritania':ab,ti,ca OR 'Micronesia':ab,ti,ca OR 'Moldova':ab,ti,ca OR 'Mongolia':ab,ti,ca OR 'Morocco':ab,ti,ca OR 'Nicaragua':ab,ti,ca OR 'Nigeria':ab,ti,ca OR 'Pakistan':ab,ti,ca OR 'Papua New Guinea':ab,ti,ca OR 'Paraguay':ab,ti,ca OR 'Philippines':ab,ti,ca OR 'Samoa':ab,ti,ca OR 'Atlantic Islands':ab,ti,ca OR 'Sao Tome':ab,ti,ca OR Principe:ab,ti,ca OR 'Senegal':ab,ti,ca OR 'Melanesia':ab,ti,ca OR 'Solomon islands':ab,ti,ca OR 'Sri Lanka':ab,ti,ca OR 'Sudan':ab,ti,ca OR 'Swaziland':ab,ti,ca OR 'Syria':ab,ti,ca OR 'East Timor':ab,ti,ca OR 'Timor leste':ab,ti,ca OR 'Ukraine':ab,ti,ca OR 'Uzbekistan':ab,ti,ca OR 'Vanuatu':ab,ti,ca OR 'Vietnam':ab,ti,ca OR 'Middle East':ab,ti,ca OR 'west bank':ab,ti,ca OR 'Gaza':ab,ti,ca OR 'Yemen':ab,ti,ca OR 'Zambia':ab,ti,ca OR 'Angola':ab,ti,ca OR 'Albania':ab,ti,ca OR 'Algeria':ab,ti,ca OR 'Argentina':ab,ti,ca OR 'Samoa':ab,ti,ca OR 'Azerbaijan':ab,ti,ca OR 'Republic of Belarus':ab,ti,ca OR 'Belize':ab,ti,ca OR Bosnia:ab,ti,ca OR Herzegovina:ab,ti,ca OR 'Botswana':ab,ti,ca OR 'Brazil':ab,ti,ca OR 'Bulgaria':ab,ti,ca OR 'China':ab,ti,ca OR 'Colombia':ab,ti,ca OR 'Costa Rica':ab,ti,ca OR 'Cuba':ab,ti,ca OR 'Dominica':ab,ti,ca OR 'Dominican Republic':ab,ti,ca OR 'Ecuador':ab,ti,ca OR 'Equatorial Guinea':ab,ti,ca OR 'Fiji':ab,ti,ca OR 'Gabon':ab,ti,ca OR 'Grenada':ab,ti,ca OR 'Iran':ab,ti,ca OR 'Iraq':ab,ti,ca OR 'Jamaica':ab,ti,ca OR 'Jordan':ab,ti,ca OR 'Kazakhstan':ab,ti,ca OR 'Lebanon':ab,ti,ca OR 'Libya':ab,ti,ca OR 'Macedonia':ab,ti,ca OR 'Malaysia':ab,ti,ca OR 'Indian Ocean Islands':ab,ti,ca OR 'Maldives':ab,ti,ca OR 'Marshall Islands':ab,ti,ca OR 'Mauritius':ab,ti,ca OR 'Mexico':ab,ti,ca OR 'Montenegro':ab,ti,ca OR 'Namibia':ab,ti,ca OR 'Palau':ab,ti,ca OR 'Panama':ab,ti,ca OR 'Peru':ab,ti,ca OR 'Romania':ab,ti,ca OR 'Russia':ab,ti,ca OR 'Russian Federation':ab,ti,ca OR 'Serbia':ab,ti,ca OR 'Seychelles':ab,ti,ca OR 'South Africa':ab,ti,ca OR 'Saint Lucia':ab,ti,ca OR 'Saint Vincent and the Grenadines':ab,ti,ca OR 'Suriname':ab,ti,ca OR 'Thailand':ab,ti,ca OR 'Tonga':ab,ti,ca OR 'Tunisia':ab,ti,ca OR 'Turkey':ab,ti,ca OR 'Turkmenistan':ab,ti,ca OR 'Tuvalu':ab,ti,ca OR 'Venezuela':ab,ti,ca OR 'low resource':ab,ti OR 'under resourced':ab,ti OR 'resource poor':ab,ti OR 'under developed':ab,ti OR 'underdeveloped':ab,ti OR 'developing country':ab,ti OR 'developing countries':ab,ti OR 'developing world':ab,ti OR 'third world':ab,ti OR lmic:ab,ti OR (low:ab,ti AND middle:ab,ti AND income:ab,ti) | [5,576,471](https://www-embase-com.proxy.lib.duke.edu/) |
| #13 | #11 AND #12 | [13,349](https://www-embase-com.proxy.lib.duke.edu/) |
| #14 | [infant]/lim OR [child]/lim OR [adolescent]/lim OR Infancy:ab,ti OR newborn:ab,ti OR newborns:ab,ti OR neonatal:ab,ti OR neonate:ab,ti OR baby:ab,ti OR babies:ab,ti OR toddler:ab,ti OR toddlers:ab,ti OR child:ab,ti OR children:ab,ti OR infant:ab,ti OR infants:ab,ti OR preschool:ab,ti OR preschooler:ab,ti OR pediatric:ab,ti OR teenager:ab,ti OR teenagers:ab,ti OR teenaged:ab,ti OR teen:ab,ti OR teens:ab,ti OR adolescent:ab,ti OR adolescents:ab,ti OR adolescence:ab,ti OR youth:ab,ti OR boy:ab,ti OR boys:ab,ti OR boyhood:ab,ti OR girl:ab,ti OR girls:ab,ti OR girlhood:ab,ti OR kid:ab,ti OR kids:ab,ti OR schoolchild:ab,ti OR "school child":ab,ti OR "school age":ab,ti OR paediatric:ab,ti OR paediatrics:ab,ti | [4,398,513](https://www-embase-com.proxy.lib.duke.edu/) |
| #15 | #13 AND #14 | [4,144](https://www-embase-com.proxy.lib.duke.edu/) |
| #16 | ('randomized controlled trial'/exp OR 'crossover procedure'/exp OR 'double blind procedure'/exp OR 'single blind procedure'/exp OR random*:ab,ti OR factorial*:ab,ti OR crossover*:ab,ti OR (cross NEAR/1 over*):ab,ti OR placebo*:ab,ti OR (doubl* NEAR/1 blind*):ab,ti OR (singl* NEAR/1 blind*):ab,ti OR assign*:ab,ti OR allocat*:ab,ti OR volunteer*:ab,ti OR 'clinical study'/exp OR ‘clinical trial’:ti,ab OR ‘clinical trials’:ti,ab OR 'controlled study'/exp OR 'evaluation'/exp OR ‘evaluation study’:ab,ti OR ‘evaluation studies’:ab,ti OR ‘intervention study’:ab,ti OR ‘intervention studies’:ab,ti OR ‘case control’:ab,ti OR 'cohort analysis'/exp OR cohort:ab,ti OR longitudinal*:ab,ti OR prospective:ab,ti OR prospectively:ab,ti OR retrospective:ab,ti OR 'follow up'/exp OR ‘follow up’:ab,ti OR 'comparative effectiveness'/exp OR 'comparative study'/exp OR ‘comparative study’:ab,ti OR ‘comparative studies’:ab,ti OR 'evidence based medicine'/exp OR ‘systematic review’:ab,ti OR ‘meta-analysis’:ab,ti OR ‘meta-analyses’:ab,ti) NOT ('case report'/exp OR 'case study'/exp OR 'editorial'/exp OR 'letter'/exp OR 'note'/exp OR [conference abstract]/lim) | [12,033,410](https://www-embase-com.proxy.lib.duke.edu/) |
| #17 | #15 AND #16 | [2,562](https://www-embase-com.proxy.lib.duke.edu/) |
| #18 | #17 AND [embase]/lim NOT [medline]/lim | [726](https://www-embase-com.proxy.lib.duke.edu/) |
| #19 | #18 AND [1-4-2019]/sd NOT [3-6-2020]/sd | [84](https://www-embase-com.proxy.lib.duke.edu/) |

Scopus

| #1 | TITLE-ABS-KEY("traffic accident'" OR (("motor vehicle" OR traffic OR vehicle OR vehicular OR car OR cars OR automobile OR automobiles OR motorcycle OR motorcycles OR taxi OR cab OR road OR pedestrian OR pedestrians) AND (accident OR accidents OR injury OR injuries))) | 164,436 |
| --- | --- | --- |
| #2 | TITLE-ABS-KEY(Law OR laws OR legal OR regulation OR regulations OR "minimum age" OR (minimum AND (drinking OR alcohol) AND age) OR "seat belt" OR "seat belts" OR restraint OR restraints OR helmet OR helmets OR speed OR safety OR light OR lights OR license OR licenses OR licensing OR prevent*) | 10,925,879 |
| #3 | TITLE-ABS-KEY(drowning OR drowned OR drowns) | 13,584 |
| #4 | TITLE-ABS-KEY(Hazard OR hazards OR fencing OR fence OR fenced OR fences OR flotation OR safety OR resuscitation OR resuscitate OR resuscitated) | 2,129,555 |
| #5 | TITLE-ABS-KEY(burn OR burns OR (smoke AND inhal*)) | 151,526 |
| #6 | TITLE-ABS-KEY("smoke alarm" OR "smoke alarms" OR "child resistant" OR "hot water" OR education OR "burn center" OR "burn centers" OR "burn unit" OR "burn units") | 2,097,140 |
| #7 | TITLE(falls OR fall) | 52,364 |
| #8 | TITLE-ABS-KEY(Furniture OR program OR programs OR intervention OR interventions OR playground OR play OR window OR windows) | 5,887,840 |
| #9 | TITLE-ABS-KEY(ingestion OR poisoning OR poisoned) | 263,686 |
| #10 | TITLE-ABS-KEY("child resistant" OR packaging OR package OR "poison control") | 450,509 |
| #11 | (#1 AND #2) OR (#3 AND #4) OR (#5 AND #6) OR (#7 AND #8) OR (#9 AND #10) | 108,824 |
| #12 | TITLE-ABS-KEY("Afghanistan" OR "Bangladesh" OR "Benin" OR "Burkina Faso" OR "Burundi" OR "Cambodia" OR "cabo verde" OR "Central African Republic" OR "Chad" OR "Comoros" OR "Congo" OR "Eritrea" OR "Ethiopia" OR "Gambia" OR "Guinea" OR "Haiti" OR "Kenya" OR "Korea" OR "Liberia" OR "Madagascar" OR "Malawi" OR "Mali" OR "Mozambique" OR "Myanmar" OR "Nepal" OR "Niger" OR "Rwanda" OR "Sierra Leone" OR "Somalia" OR "Tajikistan" OR "Tanzania" OR "Togo" OR "Uganda" OR "Zimbabwe" OR "Armenia" OR "Bhutan" OR "Bolivia" OR "Cameroon" OR "Cape Verde" OR "Congo" OR "Cote dIvoire" OR "ivory coast" OR "Djibouti" OR "Egypt" OR "El Salvador" OR "Georgia" OR "Ghana" OR "Guatemala" OR "Guyana" OR "Honduras" OR "Indonesia" OR "India" OR "Kiribati" OR "Kosovo" OR "Kyrgyzstan" OR "Kyrgyz" OR "Laos" OR "lao" OR "Lesotho" OR "Mauritania" OR "Micronesia" OR "Moldova" OR "Mongolia" OR "Morocco" OR "Nicaragua" OR "Nigeria" OR "Pakistan" OR "Papua New Guinea" OR "Paraguay" OR "Philippines" OR "Samoa" OR "Atlantic Islands" OR "Sao Tome" OR Principe OR "Senegal" OR "Melanesia" OR "Solomon islands" OR "Sri Lanka" OR "Sudan" OR "Swaziland" OR "Syria" OR "East Timor" OR "Timor leste" OR "Ukraine" OR "Uzbekistan" OR "Vanuatu" OR "Vietnam" OR "Middle East" OR "west bank" OR "Gaza" OR "Yemen" OR "Zambia" OR "Angola" OR "Albania" OR "Algeria" OR "Argentina" OR "Samoa" OR "Azerbaijan" OR "Republic of Belarus" OR "Belize" OR Bosnia OR Herzegovina OR "Botswana" OR "Brazil" OR "Bulgaria" OR "China" OR "Colombia" OR "Costa Rica" OR "Cuba" OR "Dominica" OR "Dominican Republic" OR "Ecuador" OR "Equatorial Guinea" OR "Fiji" OR "Gabon" OR "Grenada" OR "Iran" OR "Iraq" OR "Jamaica" OR "Jordan" OR "Kazakhstan" OR "Lebanon" OR "Libya" OR "Macedonia" OR "Malaysia" OR "Indian Ocean Islands" OR "Maldives" OR "Marshall Islands" OR "Mauritius" OR "Mexico" OR "Montenegro" OR "Namibia" OR "Palau" OR "Panama" OR "Peru" OR "Romania" OR "Russia" OR "Russian Federation" OR "Serbia" OR "Seychelles" OR "South Africa" OR "Saint Lucia" OR "Saint Vincent and the Grenadines" OR "Suriname" OR "Thailand" OR "Tonga" OR "Tunisia" OR "Turkey" OR "Turkmenistan" OR "Tuvalu" OR "Venezuela" OR "low resource" OR "under resourced" OR "resource poor" OR "under developed" OR "underdeveloped" OR "developing country" OR "developing countries" OR "developing world" OR "third world" OR lmic OR (low AND middle AND income)) OR AFFILCOUNTRY("Afghanistan" OR "Bangladesh" OR "Benin" OR "Burkina Faso" OR "Burundi" OR "Cambodia" OR "cabo verde" OR "Central African Republic" OR "Chad" OR "Comoros" OR "Congo" OR "Eritrea" OR "Ethiopia" OR "Gambia" OR "Guinea" OR "Haiti" OR "Kenya" OR "Korea" OR "Liberia" OR "Madagascar" OR "Malawi" OR "Mali" OR "Mozambique" OR "Myanmar" OR "Nepal" OR "Niger" OR "Rwanda" OR "Sierra Leone" OR "Somalia" OR "Tajikistan" OR "Tanzania" OR "Togo" OR "Uganda" OR "Zimbabwe" OR "Armenia" OR "Bhutan" OR "Bolivia" OR "Cameroon" OR "Cape Verde" OR "Congo" OR "Cote dIvoire" OR "ivory coast" OR "Djibouti" OR "Egypt" OR "El Salvador" OR "Georgia" OR "Ghana" OR "Guatemala" OR "Guyana" OR "Honduras" OR "Indonesia" OR "India" OR "Kiribati" OR "Kosovo" OR "Kyrgyzstan" OR "Kyrgyz" OR "Laos" OR "lao" OR "Lesotho" OR "Mauritania" OR "Micronesia" OR "Moldova" OR "Mongolia" OR "Morocco" OR "Nicaragua" OR "Nigeria" OR "Pakistan" OR "Papua New Guinea" OR "Paraguay" OR "Philippines" OR "Samoa" OR "Atlantic Islands" OR "Sao Tome" OR Principe OR "Senegal" OR "Melanesia" OR "Solomon islands" OR "Sri Lanka" OR "Sudan" OR "Swaziland" OR "Syria" OR "East Timor" OR "Timor leste" OR "Ukraine" OR "Uzbekistan" OR "Vanuatu" OR "Vietnam" OR "Middle East" OR "west bank" OR "Gaza" OR "Yemen" OR "Zambia" OR "Angola" OR "Albania" OR "Algeria" OR "Argentina" OR "Samoa" OR "Azerbaijan" OR "Republic of Belarus" OR "Belize" OR Bosnia OR Herzegovina OR "Botswana" OR "Brazil" OR "Bulgaria" OR "China" OR "Colombia" OR "Costa Rica" OR "Cuba" OR "Dominica" OR "Dominican Republic" OR "Ecuador" OR "Equatorial Guinea" OR "Fiji" OR "Gabon" OR "Grenada" OR "Iran" OR "Iraq" OR "Jamaica" OR "Jordan" OR "Kazakhstan" OR "Lebanon" OR "Libya" OR "Macedonia" OR "Malaysia" OR "Indian Ocean Islands" OR "Maldives" OR "Marshall Islands" OR "Mauritius" OR "Mexico" OR "Montenegro" OR "Namibia" OR "Palau" OR "Panama" OR "Peru" OR "Romania" OR "Russia" OR "Russian Federation" OR "Serbia" OR "Seychelles" OR "South Africa" OR "Saint Lucia" OR "Saint Vincent and the Grenadines" OR "Suriname" OR "Thailand" OR "Tonga" OR "Tunisia" OR "Turkey" OR "Turkmenistan" OR "Tuvalu" OR "Venezuela" OR "low resource" OR "under resourced" OR "resource poor" OR "under developed" OR "underdeveloped" OR "developing country" OR "developing countries" OR "developing world" OR "third world" OR lmic OR (low AND middle AND income)) | 20,233,039 |
| #13 | #11 AND #12 | 28,171 |
| #14 | TITLE-ABS-KEY(Infancy OR newborn OR newborns OR neonatal OR neonate OR baby OR babies OR toddler OR toddlers OR child OR children OR infant OR infants OR preschool OR preschooler OR pediatric OR teenager OR teenagers OR teenaged OR teen OR teens OR adolescent OR adolescents OR adolescence OR youth OR boy OR boys OR boyhood OR girl OR girls OR girlhood OR kid OR kids OR schoolchild OR "school child" OR "school age" OR paediatric OR paediatrics) | 5,338,696 |
| #15 | #13 AND #14 | 4,728 |
| #16 | TITLE-ABS-KEY( ((random OR randomly OR randomized OR factorial OR crossover) AND (trial OR study)) OR "clinical study" OR "clinical trial" OR "clinical trials" OR "controlled study" OR "evaluation study" OR "evaluation studies" OR "intervention study" OR "intervention studies" OR "case control" OR "cohort analysis" OR cohort OR longitudinal OR longitudinally OR prospective OR prospectively OR retrospective OR "follow up" OR "comparative effectiveness" OR "comparative study" OR "comparative studies" OR "systematic review" OR "meta-analysis" OR "meta-analyses") | 12,807,113 |
| #17 | #15 AND #16 | 2,847 |
| #18 | #17, exclude conference papers and letters, limit to 2019 and 2020 | 248 |

Global Index Medicus

(injury OR accident OR accidents OR drowning OR burns OR poisoning) AND prevent* AND (child OR children OR pediatric OR paediatric OR youth) AND (program OR intervention OR study OR trial)

Limit to 2019 - 2020

= 62 citations
